# Stereoselective Construction of β-chiral Homoallyl Functionalities by Substrate- and Reagent-Controlled Iterative 1,2-Metallate Rearrangements

**DOI:** 10.1021/acs.orglett.3c02935

**Published:** 2023-11-09

**Authors:** Elvira Linne, Markus Kalesse

**Affiliations:** † Institute of Organic Chemistry (OCI), Gottfried Wilhelm Leibniz Universität Hannover, 30167 Hannover, Germany; § Centre of Biomolecular Drug Research (BMWZ), Gottfried Wilhelm Leibniz Universität Hannover, 30167 Hannover, Germany

## Abstract

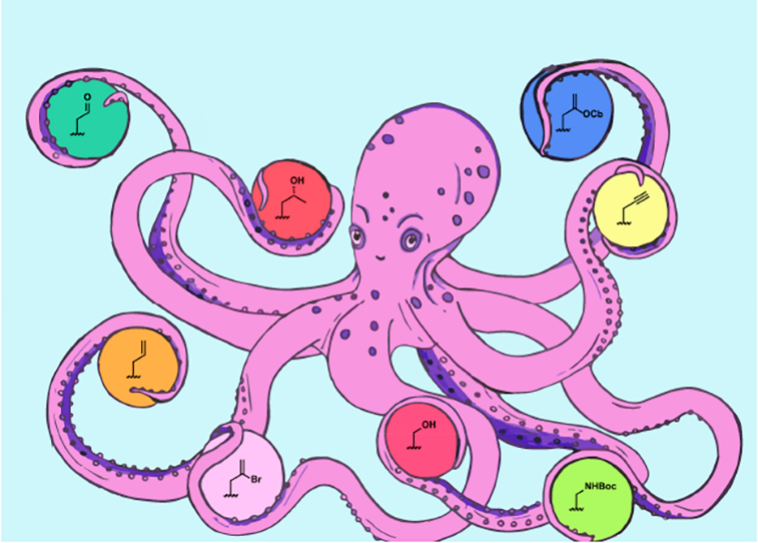

Homoallylic alcohols possessing chiral β-centers are considered highly valuable in the synthesis of polyketide-based natural products. Therefore, there is a significant demand for new methods that allow for their stereoselective access. In pursuit of this objective, we have successfully combined our substrate-controlled protocol of Hoppe–Matteson–Aggarwal chemistry with iterative 1,2-metallate rearrangements. Notably, this approach has proven effective in introducing not only primary alcohols but also other functional groups such as alkynes and protected amines.

Complex primary homoallylic functionalities bearing chiral β-centers are highly significant structural motifs found in bioactive natural products. Notable examples include the homoallylic alcohol present in the antibiotic macrodiolide luminamicin (**1**)^1^ and the homoallylic amine found in cytotoxic cyclamenol B (**2**)^2^ (Figure 1).

**Figure 1:**
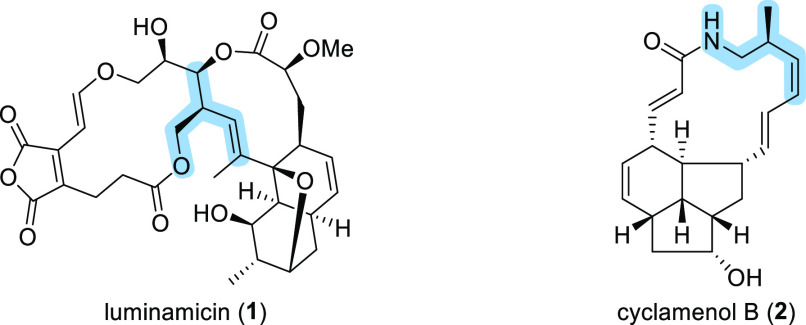
Homoallylic functionalities in bioactive compounds.

The construction of these functional group clusters typically involves aldol,^3^ olefination,^4^ or organometal reactions.^1b,5^ Recently, lithiation and borylation chemistry, pioneered by Hoppe, Matteson, and Aggarwal, has emerged as a successful strategy for the total synthesis of natural products.^6^ In our own efforts toward the total synthesis of chondrochloren A, we applied substrate control in lithiation–borylation chemistry for the first time within the context of a complex natural product synthesis.^7^ To investigate the origins of substrate- and reagent-controlled induction in 1,2-metallate rearrangements, we examined the behavior of 2,4,6-triisopropylbenzoyl- (TIB) and *N,N*-diisopropyl carbamoyl- (Cb) derived diketides using vinyl boronic esters (vbe). Our observations revealed that the TIB group and branched vbe favored the formation of Felkin products, while the Cb group favored the formation of *anti*-Felkin products (Scheme 1a).^8^ To expand upon this methodology, the secondary allylic boronic esters were subjected to Matteson homologation to explore the scope of the synthesis of β-chiral homoallylic compounds (Scheme 1b).

**Scheme 1:**
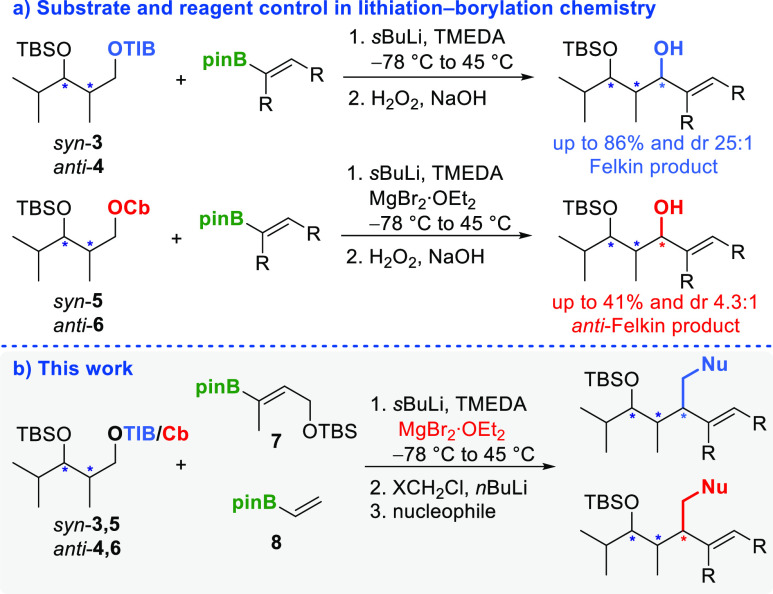
Lithiation–Borylation of Diketides

**Table 1:**
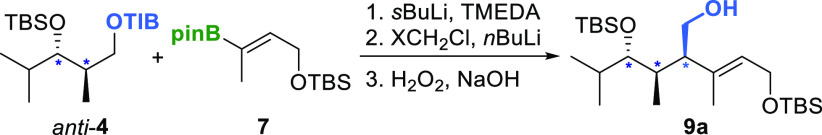
Optimization of Reaction Conditions^a^

entry	homologation reagent	temperature	yield^b^
1	BrCH_2_Cl	–78 °C to rt	26%
2	ICH_2_Cl^c^	–78 °C to rt	55%
3	ICH_2_Cl	–95 °C to rt	62%^d^
4	ICH_2_Cl	–95 °C to rt^e^	73%^f^
5	ICH_2_Cl	–95 to 45 °C^e^	63%

a Reaction conditions: **4** (0.45 mmol), **7** (0.30 mmol), *s*BuLi0.42mmol), TMEDA (0.45 mmol), Et_2_O, −78 to 45 °C, o/n. Homologation reagent (1.20 mmol), *n*BuLi (0.99 mmol), Et_2_O, temperature, o/n. H_2_O_2_, NaOH, THF, – 20 °C to rt, 0.5 h.

b Isolated yields o3s.

c THF instead of Et_2_O.

d Shorter reaction time (1 h vs o/n) led to decreased yields (40%).

e Addition of *n*BuLi at –95 °C and ate-complex formation at −78 °C.

f 1.00 mmol scale.

**Table 2:**
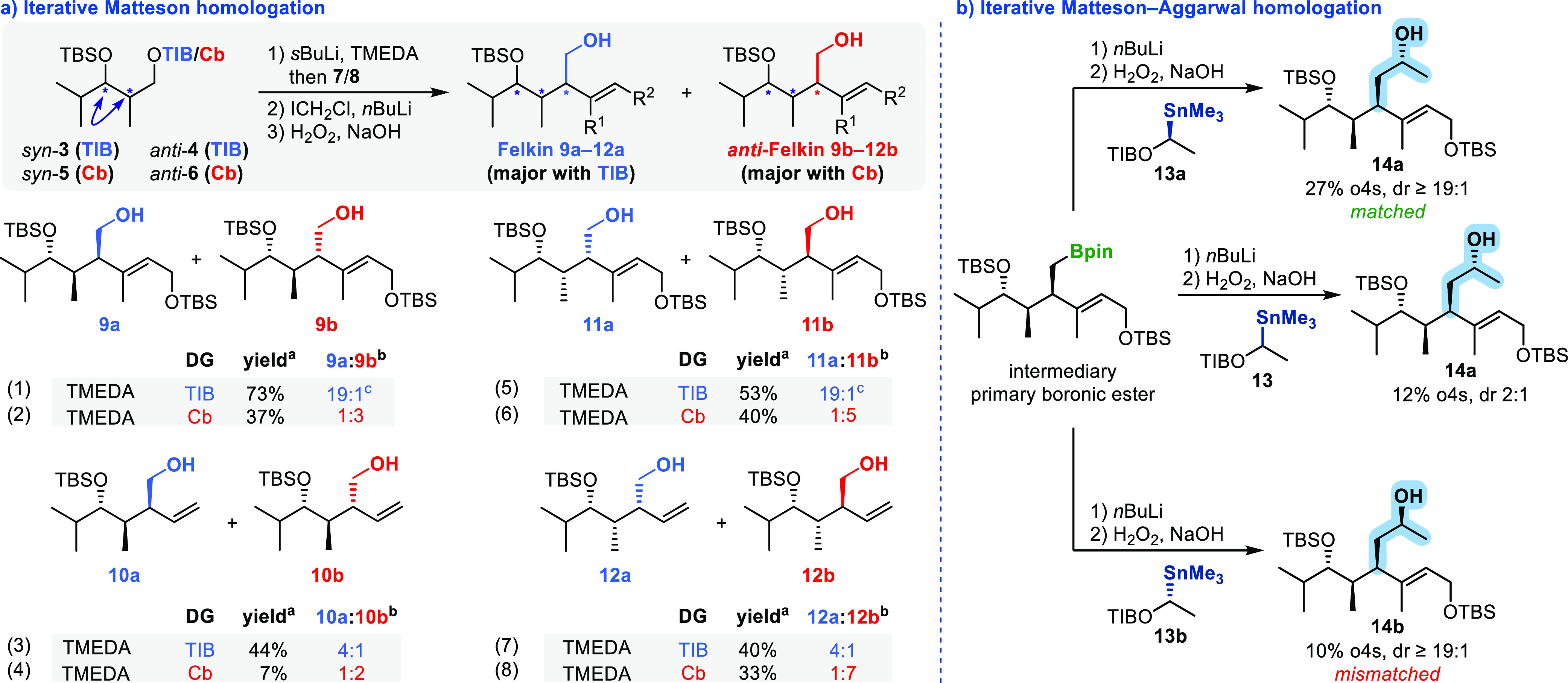
Substrate and Reagent-Induced Homologation Sequence Utilizing *syn*- and *anti*-Configured Diketides for HMA Rearrangement: a) Iterative Matteson homologation and b) Iterative Matteson–Aggarwal Homologation

a Isolated yields over three steps. For general conditions, see Table 1; for detailed information, see SI.

b dr determined by ^1^H NMR. The absolute configuration is determined by the configuration of secondary alcohols.^8^

c Attributed to NMR-accuracy.

Our investigation commenced with *syn*- and *anti*-diketides (Scheme 1b) derived from isobutyraldehyde, as the isopropyl branch resembles polyketidal frameworks. Throughout this study, we employed branched vbe **7** and unsubstituted vbe **8** (Scheme 1b) to represent the range of selectivities that can be expected. We explained the obtained lower selectivities for unbranched vbe **8** via DFT calculation-based mechanistic analysis suggesting that unbranched vbe **8** cannot differentiate between the two hemispheres in the borylation step compared to the larger vbe **7**.^8^ In the context of complex natural product syntheses, the results observed with branched vbe **7** are more representative, since several natural products feature branched double bonds.^7,8^ Therefore, the combination of TIB ester **4** and vbe **7** was chosen to assess the potential of iterative homologation. For the initial substrate and reagent-controlled lithiation–borylation chemistry, we used of an excess of TIB ester (1.5 equiv) and achiral diamine TMEDA (1.5 equiv).^7,8^

Through an examination of various Matteson homologation conditions,^9^ it was determined that the combination of chloroiodomethane and *n*-butyllithium in Et_2_O gave the best and most consistent results (Table 1, entries 3–5). The formation of the ate-complex at cryogenic temperatures (−95 to −78 °C) for a duration of 3 h, followed by the 1,2-metallate rearrangement at room temperature, resulted in the highest yield (73% over three steps (o3s), entry 4). Alternatively, increasing the temperature during the 1,2-metallate rearrangement led to a decrease in yield (entry 5).

These conditions were applied to TIB esters **3** and **4**, as well as to carbamates **5** and **6** (Table 2a). To evaluate the impact of an additional iterative homologation on yield and selectivity, a comparison was made with the secondary alcohols obtained in our previous work (see SI).^8^ By utilizing branched vbe **7** and the anion derived from *anti*-TIB ester **4**, followed by iterative Matteson homologation, Felkin product **9a** was generated with excellent yield and selectivity (73% o3s, ≥19:1, Table 2, entry 1). Interestingly, there was no significant reduction in either yield (69% o2s) or selectivity (≥19:1) compared to isolation of the secondary alcohol.^8^ As expected, the use of carbamate **6** in this sequence resulted in the formation of the opposite diastereoisomer **9b**, with a lower yield and selectivity (37% o3s, 3:1, Table 2, entry 2). As observed in our previous study,^8^ the low yields obtained from the combination of carbamates and vbe **8** can probably be attributed to a combination of inferior leaving group quality (Cb) and poor migration ability of the unsubstituted vinyl residue.^10^ Notably, the yield dropped significantly (37% vs 60%), while the diastereomeric ratio (3:1 vs 2.4:1) remained comparable to that of the secondary alcohol.

This trend was not observed when unsubstituted vbe **8** was used. In comparison to the secondary alcohol, both the yield (7% vs 11%) and selectivity (2:1 vs 2.3:1) of the β-chiral primary alcohol **10b** were comparable (Table 2, entry 4). However, when TIB ester **4** and unsubstituted vbe **8** were combined, the selectivity slightly increased (4:1 vs 2–3:1), but the yield decreased (44% vs 50–63%) (Table 2, entry 3). This can be attributed to the decomposition of the intermediary *anti*-Felkin boronic ester and/or the preferential conversion of the Felkin intermediate. On the other hand, when *syn*-TIB ester **3** and branched vbe **7** were used in the iterative sequence, β-chiral homoallylic alcohol **11a** was obtained with good yield and excellent selectivity (53% o3s, ≥19:1, Table 2, entry 5). In comparison to the diastereomeric ratio observed for the secondary alcohol (10:1), an increased selectivity (≥19:1) was achieved, but the yield decreased (53% vs 79%). Once again, this decrease in yield and concomitant increase in selectivity can be attributed to the decomposition of the *anti*-Felkin boronic ester and/or the preferential conversion of the Felkin intermediate. With the utilization of carbamate **5** and vbe **7**, the iterative Matteson homologation process gave the *anti*-Felkin product **11b** in moderate yield and selectivity (40% o3s, 5:1, Table 2, entry 6) falling within the observed range for the secondary alcohol (5:1 vs 4.3:1, 40% vs 38%). Furthermore, when employing unsubstituted vbe **8**, *syn*-TIB ester **3** exhibited higher selectivity (4:1 vs 2.1–3.1) and decreased yield (40% vs 50%) for Felkin product **12a** compared to the results obtained for the secondary alcohol (Table 2, entry 7). These deviations can be attributed to a preferred conversion of the intermediary Felkin product and/or the decomposition of the *anti*-Felkin intermediate. By utilizing *syn*-carbamate **5** and unsubstituted vbe **8** in the iterative Matteson homologation sequence, β-chiral homoallylic alcohol **12b** was obtained in a moderate yield and selectivity (33% o3s, 7:1, Table 2, entry 8). It is worth noting that both the yield (33% vs 31%) and selectivity (7:1 vs 8.1:1) of the β-chiral primary alcohol were comparable to those observed for the secondary alcohol.

To further expand upon this methodology, the impact of the existing stereocenter on subsequent lithiation–borylation chemistry was investigated. In this context, the β-chiral homoallylboronic ester obtained as an intermediate was subjected to Aggarwal homologations^11^ with stannanes **13** (Table 2b). When utilizing (−)-sparteine-derived stannane **13a**, a moderate yield of 27% over four steps was achieved for the formation of secondary alcohol **14a**, accompanied by excellent diastereoselectivity (≥19:1). Similarly, diastereoisomer **14b** was prepared using (+)-sparteine-derived stannane **13b**, also exhibiting excellent diastereoselectivity (≥19:1). However, in this case, the yield significantly decreased to 10% over four steps, indicating a mismatched situation for the (+)-sparteine-derived stannane. The reaction of the formed chiral homoallylboronic ester with racemic TMEDA-derived stannane **13**, which slightly favored the formation of secondary alcohol **14a** (2:1), confirmed this hypothesis.

Using the reliable and effective Hoppe–Matteson–Aggarwal (HMA) rearrangement homologation sequence, we conducted an examination of the installation of other functional groups. Our main focus was on introducing an amine group to form chiral homoallylic amines, similar to those found the in cyclamenols.^2,4^ The optimization process involved *anti*-TIB ester **4** and sterically demanding vbe **7** (Table 3). We investigated the use of literature-known amination reagents, methoxyamine (**15**)^9d,12^ and the aminoazanium of DABCO (**16**).^13^ To facilitate isolation, we trapped the primary amines by Boc anhydride. In case of Morken amination^9d,12^ (Table 3, entries 1–3), using lithiated methoxyamine resulted in low yields (8–12% o3s), even with different bases (*n*BuLi vs *t*BuLi) and equivalents (3.0 equiv vs 5.0 equiv). On the other hand, the aminoazanium of DABCO (**16**)^13^ provided the desired product **17** in significantly higher yield (24%), albeit under harsh conditions (Table 3, entry 4). By reducing the equivalents of the amination reagent and base, we were able to further increase the yield, leading to the formation of complex β-chiral homoallylic amines in synthetically useful yields (Table 3, entry 5).

**Table 3:**
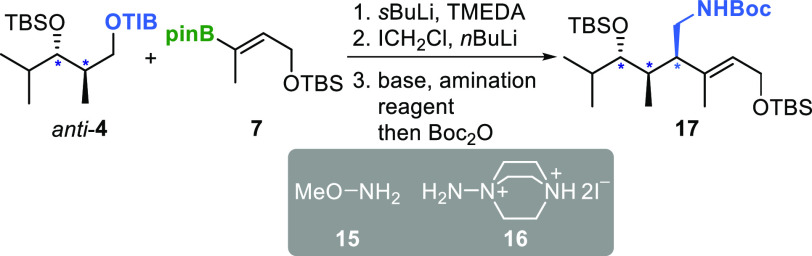
Optimization of Reaction Conditions for Implementation of Protected Amine Functionalities^a^^,^^b^^,^^c^

entry	amination reagent	base	yield^d^
1	**15** (3.0 equiv)	*n*BuLi (3.0 equiv)	8%
2	**15** (3.0 equiv)	*t*BuLi (3.0 equiv)	12%
3	**15** (5.0 equiv)	*t*BuLi (5.0 equiv)	14%
4	**16** (2.0 equiv)	KO*t*Bu (3.4 equiv)	24%
5	**16** (1.0 equiv)	KO*t*Bu (2.4 equiv)	30%

a Reaction conditions: **4** (0.30 mmol), **7** (0.20 mmol), *s*BuLi (0.28 mmol), TMEDA (0.30 mmol), Et_2_O, −78 to 45 °C, o/n. ICH_2_Cl (0.80 mmol), *n*BuLi (0.66 mmol), Et_2_O, –95 to −78 °C to rt, o/n.

b Amination using MeONH_2_: **15** (0.60/1.00 mmol), base (0.60/1.00 mmol), THF, −78 to 60 °C, o/n then Boc_2_O (0.40 mmol), rt, 2 h.

c Amination using DABCO–NH_2_: **16** (0.20/0.40 mmol), KO*t*Bu (0.48/0.68 mmol), THF, 80 °C, 2 h then Boc_2_O (0.40 mmol), rt, 2 h.

d Isolated yields o3s.

In addition to the incorporation of alcohol and amine functionalities, we also explored the introduction of other valuable functional groups (Scheme 2). The aldehyde functionality was introduced through the homologation of the β-chiral homoallylboronic ester intermediate using dichloromethyllithium, followed by oxidation. This resulted in the formation of the complex γ,δ-unsaturated aldehyde **18**, with a moderate yield of 25% over three steps.^14^

**Scheme 2:**
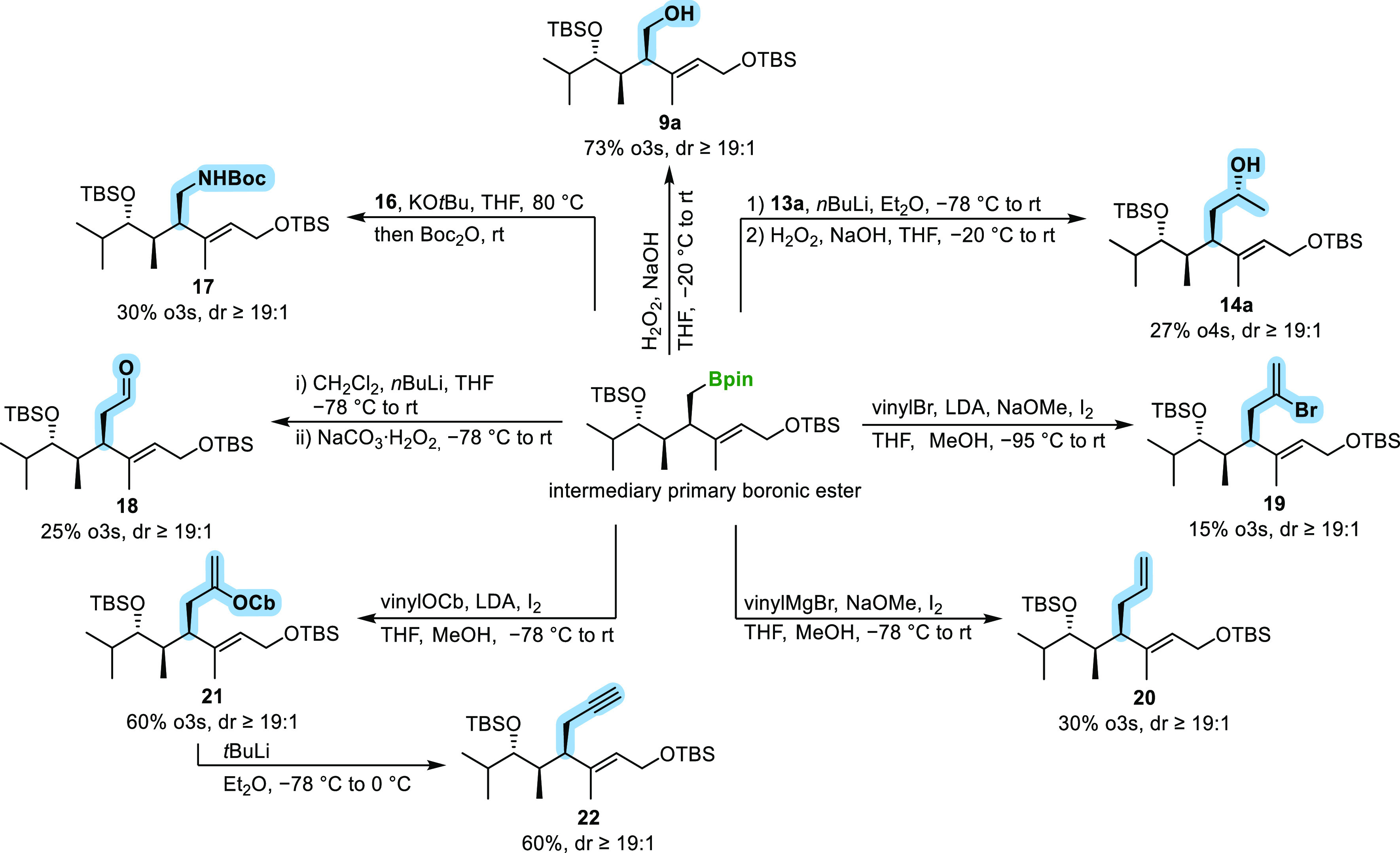
Overview of the Functional Groups Introduced through the Substrate and Reagent-Induced HMA Rearrangement Homologation Sequence of *anti*-Configured Diketide **4**.

To introduce various vinyl groups, we employed different variants of Zweifel olefination.^9c,15−17^ By utilizing vinylmagnesium bromide, we were able to generate 1,5-diene **20** in a yield of 30% over three steps.^9c,16^ Furthermore, we successfully introduced the challenging vinyl bromide unit, which serves as a potential linker for cross coupling reactions, with a yield of 17% over three steps using lithiated vinyl bromide.^9c,16,17^ The addition of lithiated vinyl carbamate led to the formation of vinyl carbamate **21**, in a good yield of 60% over three steps.^9c,16,17^ Further interconversion of **21** through an elimination reaction with *t*-butyllithium resulted in the formation of alkyne **22**, a precursor for cyclization reactions,^18^ in a yield of 60%.^16^

In conclusion, our study has demonstrated the effectiveness of the substrate- and reagent-induced HMA rearrangement homologation sequence as a valuable tool for synthesizing complex β-chiral homoallylic alcohols while preserving stereoinformation. Furthermore, we have developed an optimized protocol for introducing an amine group, resulting in the formation of β-chiral homoallylic amines, a common motif found in natural products, which differs from the current literature.^9d,12,13^ Additionally, we have successfully introduced other valuable functional groups, such as a 1,5-diene and a 1,5-enyne. By incorporating an Aggarwal homologation into our iterative sequence, we observed the influence of remote stereocenters on the stereochemical outcome. We anticipate that this highly stereoselective methodology will find further applications in natural product synthesis, and we look forward to reporting on these developments in due course.

## Data Availability

The data underlying this study are available in the published article and its Supporting Information.

## Supporting Information Available

Experimental procedures, characterization data, NMR spectra (PDF)
