# Supplementary material for: Stereoselective Construction of β-chiral Homoallyl Functionalities by Substrate- and Reagent-Controlled Iterative 1,2-Metallate Rearrangements
Source: Org Lett. 2023 Nov 9;25(46):8210–4. doi: 10.1021/acs.orglett.3c02935 (PMC10683368; doi:10.1021/acs.orglett.3c02935)
Supplement: Supplementary file 1 — ol3c02935_si_001.pdf [file ol3c02935_si_001.pdf]

Supporting Information

Stereoselective Construction of β-chiral Homoallyl Functionalities by Substrate- and Reagent-Controlled Iterative 1,2-Metallate Rearrangements

Elvira Linne and Markus Kalesse*

Institute of Organic Chemistry, Gottfried Wilhelm Leibniz Universität Hannover, Schneiderberg 1B, 30167 Hannover (Germany)

Corresponding Author

*markus.kalesse@oci.uni-hannover.de

Table of Contents

1. Comparison with Secondary Allylic Alcohols..S2

2. General Considerations...S3

3. Optimization of Reaction Conditions...S5

4. Experimental Procedures and Characterization Data.............................S6

4-1. Hoppe–Matteson–Aggarwal rearrangement Matteson homologation sequence.............................S6

4-2. Hoppe–Matteson–Aggarwal rearrangement Matteson–Aggarwal homologation sequence...........S13

4-3. Introduction of various functional groups...S16

5. References..S23

6. Spectra..S24

1. Comparison with Secondary Allylic Alcohols

In Table 1 a comparison of yields and selectivities with the secondary allylic alcohols from our previous work[1] is shown. As expected from the reaction mechanism, it was observed that the achieved selectivities were nearly equal to those of the secondary allylic alcohols. A comparison of the yields shows that with unchanged selectivities, the iterative Matteson homologation proceeds almost quantitatively.

Table 1. Comparison of yields and selectivities of obtained secondary allylic alcohols[1] with β-chiral homoallylic alcohols.

a) Secondary allylic alcohols

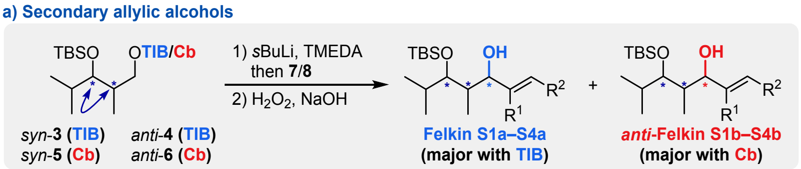

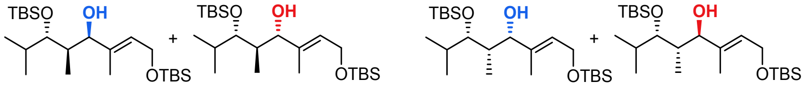

S1a + S1b; S3a + S3b

		DG	yield[a]	S1a:S1b[b]
(1)	TMEDA	TIB	69%	19:1[c]
(2)	TMEDA	Cb	60%	1:2

		DG	yield[a]	S3a:S3b[b]
(5)	TMEDA	TIB	79%	10:1
(6)	TMEDA	Cb	38%	1:4

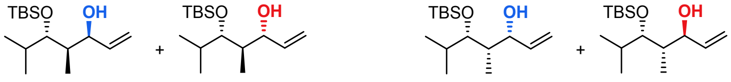

S2a + S2b; S4a + S4b

		DG	yield[a]	S2a:S2b[b]
(3)	TMEDA	TIB	50–63%	2–3:1
(4)	TMEDA	Cb	11%	1:2

		DG	yield[a]	S4a:S4b[b]
(7)	TMEDA	TIB	50%	2–3:1
(8)	TMEDA	Cb	31%	1:8

b) Iterative Matteson homologation

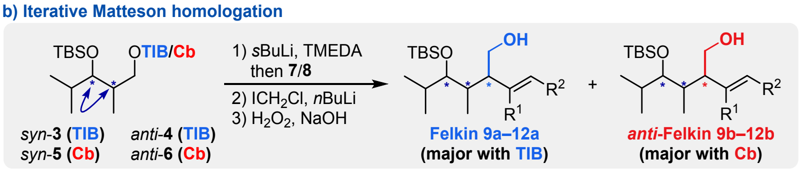

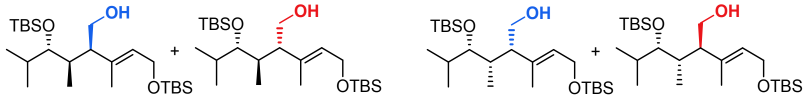

9a + 9b; 11a + 11b

		DG	yield[a]	9a:9b[b]
(9)	TMEDA	TIB	73%	19:1[c]
(10)	TMEDA	Cb	37%	1:3

		DG	yield[a]	11a:11b[b]
(13)	TMEDA	TIB	53%	19:1[c]
(14)	TMEDA	Cb	40%	1:5

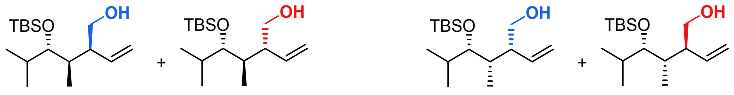

10a + 10b; 12a + 12b

		DG	yield[a]	10a:10b[b]
(11)	TMEDA	TIB	44%	4:1
(12)	TMEDA	Cb	7%	1:2

		DG	yield[a]	12a:12b[b]
(15)	TMEDA	TIB	40%	4:1
(16)	TMEDA	Cb	33%	1:7

[a]Isolated yields over two resp. three steps. [b]dr determined by ^1H NMR. [c]Attributed to NMR-accuracy.

2. General Considerations

Unless otherwise noted all reactions were carried out under an argon atmosphere using a Drierite™ gas-drying unit. The used glassware was flame dried under high vacuum. Air- and moisture-sensitive liquids and solutions were transferred via syringe flushed with argon prior to use. All reagents were purchased from commercial suppliers and used without further purification unless otherwise noted. Vinylboronic acid pinacol ester (**8**) was bought from Sigma Aldrich and Alfa Aesar and was distilled prior to use. **(+)-Sparteine** was purchased from Chem-Impex and **(−)-sparteine** was bought from TCI. Both were distilled under high vacuum and stored under argon at −25 °C. Stated temperatures, except room temperature, refer to bath temperatures (heating was conducted using oil baths).

Dry solvents Dichloromethane and all amine bases were distilled under an inert atmosphere over calcium hydride. Tetrahydrofuran, diethyl ether and methanol were purchased from Acros Organics over molecular sieves and under inert atmosphere.

Thin layer chromatography All reactions were stirred magnetically and monitored using pre-coated TLC sheets ALUGRAM® Xtra SIL G/UV_{254} (0.2 mm, silica gel, F_{254}, aluminum-backed, MACHEREY-NAGEL) with detection by UV light (λ = 254 nm) and/or by staining with acidic vanillin stain.

Flash column chromatography was performed using silica gel (0.04-0.063 mm, 240-400 mesh) obtained from MACHEREY-NAGEL. The applied petroleum ether fraction had a bp of 40-60 °C. The eluent is given in volume ratios (v/v).

^{1}H-NMR experiments were recorded in C_6D_6 using either a DPX 400 (Bruker), an AMX 400 (Bruker) or an Ascend 400 Avance III HD (Bruker). The spectra were calibrated using the residual solvent peak: $\delta(C_6D_6)$ = 7.16 ppm. Chemical shift δ is given in parts per million (ppm), coupling constant J in hertz (Hz) and multiplicity as follows: s, singlet; d, doublet; t, triplet; q, quadruplet; p, pentet; sex, sextet; sep, septet; m, multiplet; m_c, centered multiplet; brs, broad signal; or combination of these acronyms. NMR spectra were processed using TopSpin (Bruker).

^{13}C-NMR experiments were recorded in C_6D_6 using either a DPX 400 (Bruker), an AMX 400 (Bruker) or an Ascend 400 Avance III HD (Bruker). The spectra were calibrated using the residual solvent peak: $\delta(C_6D_6)$ = 128.06 ppm. Chemical shift δ is given in parts per million (ppm). NMR spectra were processed using TopSpin (Bruker).

High Resolution Mass Spectra (HRMS) were obtained either using a Q-Tof Premier (Waters), a LCT Premier (Waters) or a GC-system Agilent 6890 coupled with an Agilent 5973. Both the masses found, and the masses calculated are given.

Optical rotation $[\alpha]_D^{20}$ were measured either on a P3000 polarimeter (A. Krüss Optronic, $\lambda = 589$ nm) or a Perkin-Elmer 341 ($\lambda = 589$ nm). The sample concentration (in g/100 mL) is given with every single experiment.

3. Optimization of Reaction Conditions

Hoppe–Matteson–Aggarwal rearrangement Matteson homologation sequence

To a stirred solution of literature known TIB ester **4** (0.45 mmol, 1.5 equiv)[1] and TMEDA (0.45 mmol, 1.5 equiv) in Et_2O (0.2 M) at −78 °C was added *s*BuLi (1.3 M in hexanes, 0.42 mmol, 1.4 equiv). The reaction mixture was stirred for 5 h at that temperature before a solution of literature known vinyl boronic ester **7** (0.30 mmol, 1.0 equiv)[1,2] in Et_2O (0.5 M) was added. After stirring for further 3 h at −78 °C, the reaction mixture was warmed to 45 °C and stirred overnight. The reaction mixture was cooled to rt, sat. aq. NH_4Cl was added, and the biphasic mixture was stirred for 15 min. The phases were separated, the organic layer was washed with sat. aq. NH_4Cl (3x) and the combined aqueous phases were extracted with MTBE (3x). The combined organic phases were dried over Na_2SO_4, concentrated *in vacuo* and the crude material was purified by a short flash column chromatography (to remove TIBOH).

The crude product was dissolved in Et_2O (0.2 M), treated with the homologation reagent (1.20 mmol, 4.0 equiv) and cooled to the given temperature. *n*BuLi (1.6 M in hexanes, 0.99 mmol, 3.3 equiv) was added dropwise (0.05 mL/min) to the reaction mixture which was then stirred for 3 h at the given temperature, before being warmed to the given temperature and stirred overnight. The obtained reaction suspension was filtered over a plug of silica (Et_2O) and the filtrate was concentrated under reduced pressure.

The residue was dissolved in THF (0.2 M) and cooled to −20 °C. A premixed, ice-cooled solution of NaOH (2.0 M, 7.92 mmol, 26.4 equiv)/H_2O_2 (30%, 4.65 mmol, 15.5 equiv) was added dropwise. The reaction mixture was stirred at rt before being quenched by the slow addition of sat. aq. $Na_2S_2O_3$ at 0 °C after TLC showed full conversion (mostly 0.5 h). The solution was diluted with MTBE, the phases were separated, and the aqueous phase was extracted with MTBE (3x). The combined organic layers were dried over Na_2SO_4 and concentrated *in vacuo*. The crude product was purified by flash column chromatography to afford β-chiral homoallylic alcohol **9a**.

Table 2. Optimization of the Reaction Conditions[a]

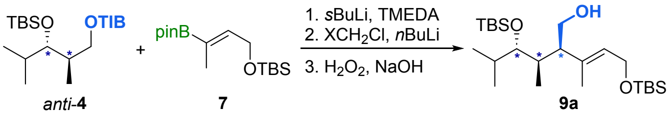

entry	homologation reagent	temperature	yield[b]
1	$BrCH_2Cl$	–78 °C to rt	26%
2	ICH_2Cl [c]	–78 °C to rt	55%
3	ICH_2Cl	–95 °C to rt	62%[d]
4	ICH_2Cl	–95 °C to rt[e]	73%[f]
5	ICH_2Cl	–95 °C to 45 °C[e]	63%

[a]Reaction conditions: **4** (0.45 mmol), **7** (0.30 mmol), *s*BuLi (0.42 mmol), TMEDA (0.45 mmol), Et_2O, –78 °C to 45 °C, o/n. Homologation reagent (1.20 mmol), *n*BuLi (0.99 mmol), Et_2O, temperature, o/n. H_2O_2, NaOH, THF, –20 °C to rt, 0.5 h. [b]Isolated yields over three steps. [c]THF was used instead of Et_2O. [d]Shorter reaction time (1 h vs o/n) led to diminishing yields (40%). [e]Addition of *n*BuLi at –95 °C and ate-complex formation at –78 °C. [f]1.00 mmol scale.

4. Experimental Procedures and Characterization Data

4-1. Hoppe–Matteson–Aggarwal rearrangement Matteson homologation sequence

General Procedure 1 (GP1): TIB esters

To a stirred solution of TIB ester (1.5 equiv) and TMEDA (1.5 equiv) in Et_2O (0.2 M) at −78 °C was added *s*BuLi (1.3 M in hexanes, 1.4 equiv). The reaction mixture was stirred for 5 h at that temperature before a solution of vinyl boronic ester (1.0 equiv) in Et_2O (0.5 M) was added. After stirring for further 3 h at −78 °C, the reaction mixture was warmed to 45 °C and stirred overnight. The reaction mixture was cooled to rt, sat. aq. NH_4Cl was added, and the biphasic mixture was stirred for 15 min. The phases were separated, the organic layer was washed with sat. aq. NH_4Cl (3x) and the combined aqueous phases were extracted with MTBE (3x). The combined organic phases were dried over Na_2SO_4, concentrated *in vacuo* and the crude material was purified by a short flash column chromatography (to remove TIBOH).
The crude product was dissolved in Et_2O (0.2 M), treated with $ClCH_2I$ (4.0 equiv) and cooled to –95 °C. *n*BuLi (1.6 M in hexanes, 3.3 equiv) was added dropwise (0.05 mL/min) to the reaction mixture which was then held for 5 min at this temperature. The vessel containing the reaction mixture was then transferred to a –78 °C cooling bath. The reaction mixture was stirred for 3 h at –78 °C, before being warmed to room temperature and stirred overnight. The obtained reaction suspension was filtered over a plug of silica (Et_2O) and the filtrate was concentrated under reduced pressure.
The residue was dissolved in THF (0.2 M) and cooled to −20 °C. A premixed, ice-cooled solution of NaOH (2.0 M, 26.4 equiv)/H_2O_2 (30%, 15.5 equiv) was added dropwise. The reaction mixture was stirred at rt before being quenched by the slow addition of sat. aq. $Na_2S_2O_3$ at 0 °C after TLC showed full conversion (mostly 0.5 h). The solution was diluted with MTBE, the phases were separated, and the aqueous phase was extracted with MTBE (3x). The combined organic layers were dried over Na_2SO_4 and concentrated *in vacuo*. The crude product was purified by flash column chromatography to afford β-chiral homoallylic alcohol.

The absolute stereochemistry is adopted by determined configuration of secondary alcohols as Matteson homologation has no effect on the previously built stereocenter.[1]

General Procedure 2 (GP2): Carbamates

To a stirred solution of carbamate (1.5 equiv) and TMEDA (1.5 equiv) in Et_2O (0.2 M) at −78 °C was added *s*BuLi (1.3 M in hexanes, 1.4 equiv). The reaction mixture was stirred for 5 h at that temperature before a solution of vinyl boronic ester (1.0 equiv) in Et_2O (0.5 M) was added. The reaction mixture was stirred for 3 h at −78 °C.
In parallel, magnesium turnings were activated (2x 1 M HCl, 2x H_2O, 2x acetone, drying under high vacuum). The required amount (2.0 equiv) was dissolved in Et_2O (0.8 M) and 1,2-dibromoethane (2.0 equiv) was added under water bath cooling. The reaction mixture was stirred for 2 h at this temperature.
The biphasic $MgBr_2·OEt_2$ solution was added dropwise to the main reaction mixture, which was then stirred for another 30 min at −78 °C before being warmed to 45 °C and stirred overnight. The reaction mixture was cooled to rt, sat. aq. NH_4Cl was added, and the biphasic

mixture was stirred for 15 min. The phases were separated, the organic layer was washed with sat. aq. NH_4Cl (3x) and the combined aqueous phases were extracted with MTBE (3x). The combined organic phases were dried over Na_2SO_4 and concentrated *in vacuo* and the crude material was purified by a short flash column chromatography (to remove excess of the carbamate).
The crude product was dissolved in Et_2O (0.2 M), treated with $ClCH_2I$ (4.0 equiv) and cooled to –95 °C. *n*BuLi (1.6 M in hexanes, 3.3 equiv) was added dropwise (0.05 mL/min) to the reaction mixture which was then held for 5 min at this temperature. The vessel containing the reaction mixture was then transferred to a –78 °C cooling bath. The reaction mixture was stirred for 3 h at –78 °C, before being warmed to room temperature and stirred overnight. The obtained reaction suspension was filtered over a plug of silica (Et_2O) and the filtrate was concentrated under reduced pressure.
The residue was dissolved in THF (0.2 M) and cooled to −20 °C. A premixed, ice-cooled solution of NaOH (2.0 M, 26.4 equiv)/H_2O_2 (30%, 15.5 equiv) was added dropwise. The reaction mixture was stirred at rt before being quenched by the slow addition of sat. aq. $Na_2S_2O_3$ at 0 °C after TLC showed full conversion (mostly 0.5 h). The solution was diluted with MTBE, the phases were separated, and the aqueous phase was extracted with MTBE (3x). The combined organic layers were dried over Na_2SO_4 and concentrated *in vacuo*. The crude product was purified by flash column chromatography to afford β-chiral homoallylic alcohol.

The absolute stereochemistry is adopted by determined configuration of secondary alcohols as Matteson homologation has no effect on the previously built stereocenter.[1]

β-Chiral homoallylic alcohol 9a – 1.0 mmol scale (According to GP1)

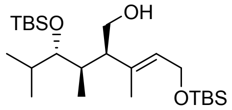

9a
$C_{24}H_{52}O_3Si_2$
444.85

To a stirred solution of literature known TIB ester **4** (715 mg, 1.50 mmol, 1.5 equiv)[1] and TMEDA (0.23 mL, 1.50 mmol, 1.5 equiv) in Et_2O (7.5 mL, 0.2 M) at −78 °C was added *s*BuLi (1.3 M in hexanes, 1.1 mL, 1.40 mmol, 1.4 equiv). The reaction mixture was stirred for 5 h at that temperature before a solution of literature known vinyl boronic ester **7** (312 mg, 1.00 mmol, 1.0 equiv)[1, 2] in Et_2O (2.0 mL, 0.5 M) was added. After stirring for further 3 h at −78 °C, the reaction mixture was warmed to 45 °C and stirred overnight. The reaction mixture was cooled to rt, sat. aq. NH_4Cl was added, and the biphasic mixture was stirred for 15 min. The phases were separated, the organic layer was washed with sat. aq. NH_4Cl (3x) and the combined aqueous phases were extracted with MTBE (3x). The combined organic phases were dried over Na_2SO_4, concentrated *in vacuo* and the crude material was purified by a short flash column chromatography (PE:MTBE 95:5) to remove TIBOH.
The crude product was dissolved in Et_2O (5.0 mL, 0.2 M), treated with $ClCH_2I$ (0.29 mL, 4.00 mmol, 4.0 equiv) and cooled to –95 °C. *n*BuLi (1.6 M in hexanes, 2.1 mL, 3.30 mmol, 3.3 equiv) was added dropwise (0.05 mL/min) to the reaction mixture which was then held for

5 min at this temperature. The vessel containing the reaction mixture was then transferred to a –78 °C cooling bath. The reaction mixture was stirred for 3 h at –78 °C, before being warmed to room temperature and stirred overnight. The obtained reaction suspension was filtered over a plug of silica (Et_2O) and the filtrate was concentrated under reduced pressure.
The residue was dissolved in THF (5.0 mL, 0.2 M) and cooled to −20 °C. A premixed, ice-cooled solution of NaOH (2.0 M, 5.2 mL, 26.4 equiv)/H_2O_2 (30%, 2.7 mL, 15.5 equiv) was added dropwise. The reaction mixture was stirred for 0.5 h at rt before being quenched by the slow addition of sat. aq. $Na_2S_2O_3$ at 0 °C. The solution was diluted with MTBE, the phases were separated, and the aqueous phase was extracted with MTBE (3x). The combined organic layers were dried over Na_2SO_4 and concentrated *in vacuo*. The crude product was purified by flash column chromatography (PE:MTBE 95:5 → 5:1) to afford β-chiral homoallylic alcohol **9a** (325 mg, 0.73 mmol, 73% o3s, dr ≥ 19:1) as a colorless oil.

^{1}H-NMR (400 MHz, C_6D_6): δ = 5.47 (m_c, 1H), 4.15 (m_c, 2H), 3.66–3.62 (m, 1H), 3.46–3.41 (m, 2H), 2.15 (m_c, 1H), 1.85–1.76 (m, 2H), 1.53 (s, 3H), 1.50 (brs, 1H), 1.02 (s, 9H), 1.00 (s, 9H), 0.97–0.94 (m, 6H), 0.90 (d, 3H, J = 7.0 Hz), 0.10–0.08 (m, 12H) ppm;

^{13}C{^{1}H}-NMR (101 MHz, C_6D_6): δ = 136.8, 128.9, 78.2, 61.9, 59.9, 52.9, 39.2, 29.5, 26.3, 26.2, 22.8, 18.6, 18.5, 18.2, 14.2, 12.6, –3.6, –4.0, –5.0, –5.0 ppm;

HRMS (ESI) m/z: calcd for $C_{24}H_{52}O_3Si_2Na$ $[M+Na]^+$ 467.3353, found: 467.3340;

R$_f$ = 0.2 (PE:MTBE 9:1, vanillin);

$[\alpha]_D^{20}$ = −2.2 (c 0.8, $CHCl_3$).

β-Chiral homoallylic alcohol 9b

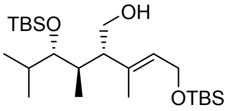

9b
$C_{24}H_{52}O_3Si_2$
444.85

According to GP2, literature known carbamate **6** (168 mg, 0.45 mmol, 1.5 equiv),[1] literature known vinyl boronic ester **7** (94.0 mg, 0.30 mmol, 1.0 equiv),[1, 2] *s*BuLi (0.32 mL, 0.42 mmol, 1.4 equiv), TMEDA (0.07 mL, 0.45 mmol, 1.5 equiv), magnesium turnings (14.6 mg, 0.60 mmol, 2.0 equiv), dibromoethane (0.05 mL, 0.60 mmol, 2.0 equiv), $ClCH_2I$ (0.09 mL, 1.20 mmol, 4.0 equiv) and *n*BuLi (0.62 mL, 0.99 mmol, 3.3 equiv) gave β-chiral homoallylic alcohol **9b** (50.4 mg, 0.11 mmol, 37% o3s, dr 3:1) after purification by flash column chromatography (PE:MTBE 95:5 → 5:1) as a colorless oil.

^{1}H-NMR (400 MHz, C_6D_6): δ = 5.59 (m_c, 1H), 4.20 (m_c, 2H), 3.70–3.61 (m, 1H), 3.52 (t, 1H, J = 3.8 Hz), 3.47–3.41 (m, 1H), 2.18–2.12 (m, 1H), 1.89–1.71 (m, 2H), 1.47 (s, 3H), 1.15 (brs, 1H), 1.02–0.99 (m, 18H), 0.98–0.95 (m, 6H), 0.90 (d, 3H, J = 6.8 Hz), 0.11–0.08 (m, 12H) ppm;

$^{13}C\{^1H\}$-NMR (101 MHz, C_6D_6): δ = 136.5, 129.7, 78.0, 62.8, 60.0, 54.7, 39.3, 30.4, 26.3, 26.2, 22.5, 18.9, 18.5, 18.5, 14.6, 13.2, –3.8, –3.4, –4.9, –4.9 ppm;

HRMS (ESI) m/z: calcd for $C_{24}H_{52}O_3Si_2Na$ $[M+Na]^+$ 467.3353, found: 467.3345;

$\mathbf{R}_f$ = 0.2 (PE:MTBE 9:1, vanillin);

$[\alpha]_D^{20}$ = −16.7 (*c* 0.3, $CHCl_3$).

β-Chiral homoallylic alcohol 10a

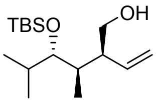

10a
$C_{16}H_{34}O_2Si$
286.53

According to GP1, literature known TIB ester **4** (296 mg, 0.62 mmol, 1.5 equiv),[1] vinyl boronic ester **8** (60.0 mg, 0.41 mmol, 1.0 equiv), *s*BuLi (0.44 mL, 0.57 mmol, 1.4 equiv), TMEDA (0.09 mL, 0.62 mmol, 1.5 equiv), $ClCH_2I$ (0.12 mL, 1.64 mmol, 4.0 equiv) and *n*BuLi (0.84 mL, 1.35 mmol, 3.3 equiv) gave β-chiral homoallylic alcohol **10a** (51.6 mg, 0.18 mmol, 44% o3s, dr 4:1) after purification by flash column chromatography (PE:MTBE 95:5 → 5:1) as a colorless oil.

^1H-NMR (400 MHz, C_6D_6): δ = 5.57 (m_c, 1H), 4.99–4.93 (m, 2H), 3.68–3.62 (m, 1H), 3.41–3.33 (m, 2H), 2.41–2.31 (m, 1H), 1.98 (m_c, 1H), 1.88–1.71 (m, 2H), 1.01 (s, 9H), 0.93 (d, 3H, *J* = 6.7 Hz), 0.86 (d, 3H, *J* = 7.2 Hz), 0.86 (d, 3H, *J* = 6.9 Hz), 0.09 (s, 3H), 0.06 (s, 3H) ppm;

$^{13}C\{^1H\}$-NMR (101 MHz, C_6D_6): δ = 140.5, 116.4, 79.7, 62.4, 47.7, 40.5, 30.4, 26.4, 21.5, 19.0, 18.7, 12.5, –3.5, –3.8 ppm;

HRMS (ESI) m/z: calcd for $C_{16}H_{34}O_2SiNa$ $[M+Na]^+$ 309.2226, found: 309.2216;

$\mathbf{R}_f$ = 0.2 (PE:MTBE 9:1, vanillin);

$[\alpha]_D^{20}$ = −2.0 (*c* 0.5, $CHCl_3$).

β-Chiral homoallylic alcohol 10b

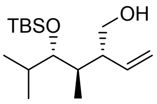

10b
$C_{16}H_{34}O_2Si$
286.53

According to GP2, literature known carbamate **6** (168 mg, 0.45 mmol, 1.5 equiv),[1] vinyl boronic ester **8** (46.2 mg, 0.30 mmol, 1.0 equiv), *s*BuLi (0.32 mL, 0.42 mmol, 1.4 equiv), TMEDA (0.07 mL, 0.45 mmol, 1.5 equiv), magnesium turnings (14.6 mg, 0.60 mmol, 2.0 equiv), dibromoethane (0.05 mL, 0.60 mmol, 2.0 equiv), $ClCH_2I$ (0.09 mL, 1.20 mmol,

4.0 equiv) and *n*BuLi (0.62 mL, 0.99 mmol, 3.3 equiv) gave β-chiral homoallylic alcohol **10b** (5.9 mg, 0.02 mmol, 7% o3s, dr 2:1) after purification by flash column chromatography (PE:MTBE 95:5 → 5:1) as a colorless oil.

^{1}H-NMR (400 MHz, C_6D_6): δ = 5.56 (m$_c$, 1H), 5.05–4.93 (m, 2H), 3.51–3.44 (m, 1H), 3.41–3.33 (m, 2H), 2.42–2.31 (m, 1H), 1.88–1.72 (m, 2H), 1.36 (brs, 1H), 1.02 (s, 9H), 0.94 (d, 3H, J = 6.8 Hz), 0.91 (d, 3H, J = 6.9 Hz), 0.83 (d, 3H, J = 7.2 Hz), 0.12 (s, 3H), 0.09 (s, 3H) ppm;

^{13}C{^{1}H}-NMR (101 MHz, C_6D_6): δ = 138.8, 117.0, 79.6, 64.3, 48.0, 38.7, 30.9, 26.5, 21.5, 18.8, 17.9, 13.5, –3.4, –3.8 ppm;

HRMS (ESI) m/z: calcd for $C_{16}H_{34}O_2SiNa$ [M+Na]$^+$ 309.2226, found: 309.2217;

R$_f$ = 0.2 (PE:MTBE 9:1, vanillin);

$[\alpha]_D^{20}$ = −1.4 (*c* 0.7, $CHCl_3$).

β-Chiral homoallylic alcohol 11a

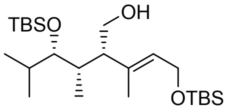

11a
$C_{24}H_{52}O_3Si_2$
444.85

According to GP1, literature known TIB ester **3** (215 mg, 0.45 mmol, 1.5 equiv),[1] literature known vinyl boronic ester **7** (94.0 mg, 0.30 mmol, 1.0 equiv),[1, 2] *s*BuLi (0.32 mL, 0.42 mmol, 1.4 equiv), TMEDA (0.07 mL, 0.45 mmol, 1.5 equiv), $ClCH_2I$ (0.09 mL, 1.20 mmol, 4.0 equiv) and *n*BuLi (0.62 mL, 0.99 mmol, 3.3 equiv) gave β-chiral homoallylic alcohol **11a** (72.2 mg, 0.16 mmol, 53% o3s, dr ≥ 19:1) after purification by flash column chromatography (PE:MTBE 95:5 → 5:1) as a colorless oil.

^{1}H-NMR (400 MHz, C_6D_6): δ = 5.63 (m$_c$, 1H), 4.17 (m$_c$, 2H), 3.66–3.61 (m, 1H), 3.54 (dd, 1H, J = 5.4, 1.5 Hz), 3.41 (m$_c$, 1H), 2.27 (m$_c$, 1H), 1.74–1.67 (m, 2H), 1.46 (s, 3H), 1.02 (s, 9H), 1.00 (brs, 10H), 0.92–0.87 (m, 9H), 0.16 (s, 3H), 0.12 (s, 3H), 0.10 (s, 6H) ppm;

^{13}C{^{1}H}-NMR (101 MHz, C_6D_6): δ = 136.9, 130.2, 78.0, 61.8, 59.9, 55.0, 34.5, 34.2, 26.5, 26.2, 19.4, 19.4, 18.9, 18.5, 13.8, 12.6, –3.1, –3.5, –5.0, –5.0 ppm;

HRMS (ESI) m/z: calcd for $C_{24}H_{52}O_3Si_2Na$ [M+Na]$^+$ 467.3353, found: 467.3355;

R$_f$ = 0.2 (PE:MTBE 9:1, vanillin);

$[\alpha]_D^{20}$ = +6.7 (*c* 0.6, $CHCl_3$).

β-Chiral homoallylic alcohol 11b

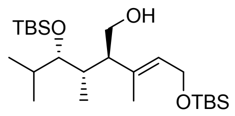

11b
$C_{24}H_{52}O_3Si_2$
444.85

According to GP2, literature known carbamate **5** (168 mg, 0.45 mmol, 1.5 equiv),[1] literature known vinyl boronic ester **7** (94.0 mg, 0.30 mmol, 1.0 equiv),[1, 2] *s*BuLi (0.32 mL, 0.42 mmol, 1.4 equiv), TMEDA (0.07 mL, 0.45 mmol, 1.5 equiv), magnesium turnings (14.6 mg, 0.60 mmol, 2.0 equiv), dibromoethane (0.05 mL, 0.60 mmol, 2.0 equiv), $ClCH_2I$ (0.09 mL, 1.20 mmol, 4.0 equiv) and *n*BuLi (0.62 mL, 0.99 mmol, 3.3 equiv) gave β-chiral homoallylic alcohol **11b** (51.8 mg, 0.12 mmol, 40% o3s, dr 5:1) after purification by flash column chromatography (PE:MTBE 95:5 → 5:1) as a colorless oil.

^{1}H-NMR (400 MHz, C_6D_6): δ = 5.64 (m_c, 1H), 4.20 (d, 2H, J = 6.1 Hz), 3.80–3.72 (m, 1H), 3.47–3.39 (m, 2H), 2.35 (m_c, 1H), 1.82–1.64 (m, 2H), 1.45 (s, 3H), 1.35 (brs, 1H), 1.01 (s, 9H), 0.99 (s, 9H), 0.92 (d, 3H, J = 6.8 Hz), 0.90 (d, 3H, J = 7.0 Hz), 0.81 (d, 3H, J = 6.8 Hz), 0.15 (s, 3H), 0.10 (s, 6H), 0.08 (s, 3H) ppm;

^{13}C{^{1}H}-NMR (101 MHz, C_6D_6): δ = 136.7, 130.1, 78.7, 62.8, 59.9, 54.2, 35.9, 33.2, 26.5, 26.2, 20.4, 19.9, 18.8, 18.5, 13.4, 13.0, –3.4, –3.6, –4.9, –4.9 ppm;

HRMS (ESI) m/z: calcd for $C_{24}H_{52}O_3Si_2Na$ $[M+Na]^+$ 467.3353, found: 467.3358;

R$_f$ = 0.2 (PE:MTBE 9:1, vanillin);

$[\alpha]_D^{20}$ = +8.3 (*c* 0.6, $CHCl_3$).

β-Chiral homoallylic alcohol 12a

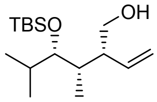

12a
$C_{16}H_{34}O_2Si$
286.53

According to GP1, literature known TIB ester **3** (215 mg, 0.45 mmol, 1.5 equiv),[1] vinyl boronic ester **8** (46.0 mg, 0.30 mmol, 1.0 equiv), *s*BuLi (0.32 mL, 0.42 mmol, 1.4 equiv), TMEDA (0.07 mL, 0.45 mmol, 1.5 equiv), $ClCH_2I$ (0.09 mL, 1.20 mmol, 4.0 equiv) and *n*BuLi (0.62 mL, 0.99 mmol, 3.3 equiv) gave β-chiral homoallylic alcohol **12a** (35.4 mg, 0.12 mmol, 40% o3s, dr 4:1) after purification by flash column chromatography (PE:MTBE 95:5 → 5:1) as a colorless oil.

^{1}H-NMR (400 MHz, C_6D_6): δ = 5.54–5.41 (m, 1H), 5.07–4.96 (m, 2H), 3.62–3.51 (m, 2H), 3.33–3.27 (m, 1H), 2.17 (m_c, 1H), 1.71 (m_c, 1H), 1.62 (m_c, 1H), 1.01 (s, 9H), 0.97 (brs, 1H), 0.92 (d, 3H, J = 6.9 Hz), 0.88–0.84 (m, 6H), 0.08 (s, 3H), 0.07 (s, 3H) ppm;

^{13}C{^{1}H}-NMR (101 MHz, C_6D_6): δ = 141.0, 117.6, 78.1, 63.3, 50.6, 36.3, 34.1, 26.5, 19.3, 19.2, 18.8, 12.5, –3.3, –3.5 ppm;

HRMS (ESI) m/z: calcd for $C_{16}H_{34}O_2SiNa$ $[M+Na]^+$ 309.2226, found: 309.2224;

R$_f$ = 0.2 (PE:MTBE 9:1, vanillin);

$[\alpha]_D^{20}$ = +10.0 (*c* 0.6, $CHCl_3$).

β-Chiral homoallylic alcohol 12b

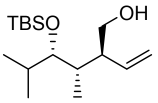

12b
$C_{16}H_{34}O_2Si$
286.53

According to GP2, literature known carbamate **5** (168 mg, 0.45 mmol, 1.5 equiv),[1] vinyl boronic ester **8** (46.3 mg, 0.30 mmol, 1.0 equiv), *s*BuLi (0.32 mL, 0.42 mmol, 1.4 equiv), TMEDA (0.07 mL, 0.45 mmol, 1.5 equiv), magnesium turnings (14.6 mg, 0.60 mmol, 2.0 equiv), dibromoethane (0.05 mL, 0.60 mmol, 2.0 equiv), $ClCH_2I$ (0.09 mL, 1.20 mmol, 4.0 equiv) and *n*BuLi (0.62 mL, 0.99 mmol, 3.3 equiv) gave β-chiral homoallylic alcohol **12b** (28.8 mg, 0.10 mmol, 33% o3s, dr 7:1) after purification by flash column chromatography (PE:MTBE 95:5 → 5:1) as a colorless oil.

^{1}H-NMR (400 MHz, C_6D_6): δ = 5.51–5.42 (m, 1H), 5.03–4.96 (m, 2H), 3.57–3.52 (m, 1H), 3.38 (t, 1H, J = 4.7 Hz), 3.33–3.29 (m, 1H), 2.24 (m_c, 1H), 1.80 (m_c, 1H), 1.72 (m_c, 1H), 1.16 (brs, 1H), 1.01 (s, 9H), 0.92 (d, 3H, J = 6.8 Hz), 0.89 (d, 3H, J = 7.0 Hz), 0.87 (d, 3H, J = 6.9 Hz), 0.10 (s, 3H), 0.07 (s, 3H) ppm;

^{13}C{^{1}H}-NMR (101 MHz, C_6D_6): δ = 138.5, 117.7, 79.0, 64.3, 49.8, 36.5, 32.3, 26.5, 20.3, 18.8, 18.2, 13.0, –3.3, –3.4 ppm;

HRMS (ESI) m/z: calcd for $C_{16}H_{34}O_2SiNa$ $[M+Na]^+$ 309.2226, found: 309.2224;

R$_f$ = 0.2 (PE:MTBE 9:1, vanillin);

$[\alpha]_D^{20}$ = +0.3 (*c* 2.2, $CHCl_3$).

4-2. Hoppe–Matteson–Aggarwal rearrangement Matteson–Aggarwal homologation sequence

General Procedure 3 (GP3): Iterative Matteson–Aggarwal homologation

To a stirred solution of TIB ester (1.5 equiv) and TMEDA (1.5 equiv) in Et_2O (0.2 M) at −78 °C was added *s*BuLi (1.3 M in hexanes, 1.4 equiv). The reaction mixture was stirred for 5 h at that temperature before a solution of vinyl boronic ester (1.0 equiv) in Et_2O (0.5 M) was added. After stirring for further 3 h at −78 °C, the reaction mixture was warmed to 45 °C and stirred overnight. The reaction mixture was cooled to rt, sat. aq. NH_4Cl was added, and the biphasic mixture was stirred for 15 min. The phases were separated, the organic layer was washed with sat. aq. NH_4Cl (3x) and the combined aqueous phases were extracted with MTBE (3x). The combined organic phases were dried over Na_2SO_4, concentrated *in vacuo* and the crude material was purified by a short flash column chromatography (to remove TIBOH).
The crude product was dissolved in Et_2O (0.2 M), treated with $ClCH_2I$ (4.0 equiv) and cooled to –95 °C. *n*BuLi (1.6 M in hexanes, 3.3 equiv) was added dropwise (0.05 mL/min) to the reaction mixture which was then held for 5 min at this temperature. The vessel containing the reaction mixture was then transferred to a –78 °C cooling bath. The reaction mixture was stirred for 3 h at –78 °C, before being warmed to room temperature and stirred overnight. The obtained reaction suspension was filtered over a plug of silica (Et_2O) and the filtrate was concentrated under reduced pressure.
To a stirred solution of the corresponding stannane (1.5 equiv) in Et_2O (0.2 M) at –78 °C was added *n*BuLi (1.6 M in hexanes, 1.65 equiv). The reaction mixture was stirred for 1 h at that temperature before a solution of the obtained β-chiral homoallylboronic ester in Et_2O (0.5 M) was added. After stirring for further 3 h at −78 °C, the reaction mixture was warmed to 45 °C and stirred overnight. The reaction mixture was cooled to rt, sat. aq. NH_4Cl was added, and the biphasic mixture was stirred for 15 min. The phases were separated, the aqueous phase was extracted with MTBE (3x). The combined organic phases were dried over Na_2SO_4 and concentrated *in vacuo*.
The residue was dissolved in THF (0.2 M) and cooled to −20 °C. A premixed, ice-cooled solution of NaOH (2.0 M, 26.4 equiv)/H_2O_2 (30%, 15.5 equiv) was added dropwise. The reaction mixture was stirred at rt before being quenched by the slow addition of sat. aq. $Na_2S_2O_3$ at 0 °C after TLC showed full conversion (mostly 0.5 h). The solution was diluted with MTBE, the phases were separated, and the aqueous phase was extracted with MTBE (3x). The combined organic layers were dried over Na_2SO_4 and concentrated *in vacuo*. The crude product was purified by flash column chromatography to afford secondary alcohol.

Stereochemistry was assigned by usual induction of (+)- and (–)-sparteine.[3, 4]

Secondary alcohol 14a

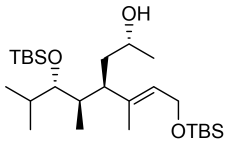

14a
$C_{26}H_{56}O_3Si_2$
472.90

According to GP3, literature known TIB ester **4** (215 mg, 0.45 mmol, 1.5 equiv),[1] literature known vinyl boronic ester **7** (94.0 mg, 0.30 mmol, 1.0 equiv),[1, 2] *s*BuLi (0.32 mL, 0.42 mmol, 1.4 equiv), TMEDA (0.07 mL, 0.45 mmol, 1.5 equiv), $ClCH_2I$ (0.09 mL, 1.20 mmol, 4.0 equiv), *n*BuLi (0.62 mL, 0.99 mmol, 3.3 equiv), literature known (–)-sp-stannane **13a** (198 mg, 0.45 mmol, 1.5 equiv)[5] and *n*BuLi (0.31 mL, 0.50 mmol, 1.65 equiv) gave secondary alcohol **14a** (37.9 mg, 0.08 mmol, 27% o4s, dr ≥ 19:1) after purification by flash column chromatography (PE:MTBE 9:1) as a colorless oil.
According to GP3, literature known TIB ester **4** (215 mg, 0.45 mmol, 1.5 equiv),[1] literature known vinyl boronic ester **7** (94.0 mg, 0.30 mmol, 1.0 equiv),[1, 2] *s*BuLi (0.32 mL, 0.42 mmol, 1.4 equiv), TMEDA (0.07 mL, 0.45 mmol, 1.5 equiv), $ClCH_2I$ (0.09 mL, 1.20 mmol, 4.0 equiv), *n*BuLi (0.62 mL, 0.99 mmol, 3.3 equiv), literature known TMEDA-stannane **13** (198 mg, 0.45 mmol, 1.5 equiv)[5] and *n*BuLi (0.31 mL, 0.50 mmol, 1.65 equiv) gave secondary alcohol **14a** (11.4 mg, 24.1 µmol, 8% o4s, dr ≥ 19:1) and secondary alcohol **14b** (5.7 mg, 12.0 µmol, 4% o4s, dr ≥ 19:1) after purification by flash column chromatography (PE:MTBE 9:1) as colorless oils.

^{1}H-NMR (400 MHz, C_6D_6): δ = 5.44 (m_c, 1H), 4.08 (d, 2H, J = 6.5 Hz), 3.64 (m_c, 1H), 3.47 (m_c, 1H), 1.91 (m_c, 1H), 1.83 (m_c, 1H), 1.75–1.66 (m, 1H), 1.57 (s, 3H), 1.54–1.41 (m, 3H), 1.13 (d, 3H, J = 6.1 Hz), 1.03 (s, 9H), 1.02–0.97 (m, 18H), 0.10 (s, 6H), 0.09 (s, 6H) ppm;

^{13}C{^{1}H}-NMR (101 MHz, C_6D_6): δ = 139.7, 127.5, 77.1, 67.4, 59.5, 50.6, 42.8, 40.2, 28.7, 26.3, 26.2, 23.5, 23.5, 18.6, 18.5, 18.1, 12.9, 12.7, –3.8, –4.2, –5.1, –5.1 ppm;

HRMS (ESI) m/z: calcd for $C_{26}H_{56}O_3Si_2Na$ $[M+Na]^+$ 495.3666, found: 495.3665;

R_f = 0.3 (PE:MTBE 95:5, vanillin);

$[\alpha]_D^{20}$ = +11.8 (*c* 0.8, $CHCl_3$).

Secondary alcohol 14b

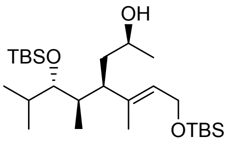

14b

$C_{26}H_{56}O_3Si_2$

472.90

According to GP3, literature known TIB ester **4** (215 mg, 0.45 mmol, 1.5 equiv),[1] literature known vinyl boronic ester **7** (94.0 mg, 0.30 mmol, 1.0 equiv),[1, 2] *s*BuLi (0.32 mL, 0.42 mmol, 1.4 equiv), TMEDA (0.07 mL, 0.45 mmol, 1.5 equiv), $ClCH_2I$ (0.09 mL, 1.20 mmol, 4.0 equiv), *n*BuLi (0.62 mL, 0.99 mmol, 3.3 equiv), literature known (+)-sp-stannane **13b** (198 mg, 0.45 mmol, 1.5 equiv)[5] and *n*BuLi (0.31 mL, 0.50 mmol, 1.65 equiv) gave secondary alcohol **14b** (14.3 mg, 0.03 mmol, 10% o4s, dr ≥ 19:1) after purification by flash column chromatography (PE:MTBE 9:1) as a colorless oil.

^{1}H-NMR (400 MHz, C_6D_6): δ = 5.49 (m_c, 1H), 4.17 (m_c, 2H), 3.64–3.56 (m, 1H), 3.52 (m_c, 1H), 2.32 (m_c, 1H), 1.89 (m_c, 1H), 1.71 (m_c, 1H), 1.59–1.52 (m, 1H), 1.48 (s, 3H), 1.23–1.10 (m, 2H), 1.08–1.00 (m, 30H), 0.13 (s, 3H), 0.12 (s, 3H), 0.11 (s, 6H) ppm;

^{13}C{^{1}H}-NMR (101 MHz, C_6D_6): δ = 138.1, 127.9 (covered by C_6D_6), 77.5, 64.9, 59.8, 48.1, 42.7, 39.7, 28.7, 26.3, 26.2, 24.7, 23.6, 18.6, 18.5, 18.0, 12.9, 12.7, –3.7, –4.2, –5.0, –5.1 ppm;

HRMS (ESI) m/z: calcd for $C_{26}H_{56}O_3Si_2Na$ $[M+Na]^+$ 495.3666, found: 495.3650;

R*f* = 0.2 (PE:MTBE 95:5, vanillin);

$[\alpha]_D^{20}$ = −0.7 (*c* 2.5, $CHCl_3$).

4-3. Introduction of various functional groups

β-Chiral homoallylic Boc-protected amine 17

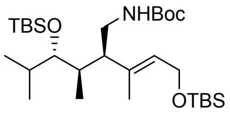

17
$C_{29}H_{61}NO_4Si_2$
543.98

To a stirred solution of literature known TIB ester **4** (143 mg, 0.30 mmol, 1.5 equiv)[1] and TMEDA (0.05 mL, 0.30 mmol, 1.5 equiv) in Et_2O (1.5 mL, 0.2 M) at −78 °C was added *s*BuLi (1.3 M in hexanes, 0.22 mL, 0.28 mmol, 1.4 equiv). The reaction mixture was stirred for 5 h at that temperature before a solution of literature known vinyl boronic ester **7** (62.2 mg, 0.20 mmol, 1.0 equiv)[1, 2] in Et_2O (0.40 mL, 0.5 M) was added. After stirring for further 3 h at −78 °C, the reaction mixture was warmed to 45 °C and stirred overnight. The reaction mixture was cooled to rt, sat. aq. NH_4Cl was added, and the biphasic mixture was stirred for 15 min. The phases were separated, the organic layer was washed with sat. aq. NH_4Cl (3x) and the combined aqueous phases were extracted with MTBE (3x). The combined organic phases were dried over Na_2SO_4, concentrated *in vacuo* and the crude material was purified by a short flash column chromatography (PE:MTBE 95:5) to remove TIBOH.
The crude product was dissolved in Et_2O (1.0 mL, 0.2 M), treated with $ClCH_2I$ (0.06 mL, 0.80 mmol, 4.0 equiv) and cooled to –95 °C. *n*BuLi (1.6 M in hexanes, 0.41 mL, 0.66 mmol, 3.3 equiv) was added dropwise (0.05 mL/min) to the reaction mixture which was then held for 5 min at this temperature. The vessel containing the reaction mixture was then transferred to a –78 °C cooling bath. The reaction mixture was stirred for 3 h at –78 °C, before being warmed to room temperature and stirred overnight. The obtained reaction suspension was filtered over a plug of silica (Et_2O) and the filtrate was concentrated under reduced pressure.
In the glovebox, a 5 mL flame-dried Schlenk tube equipped with a magnetic stirring bar was charged with literature known DABCO–NH_2 (**16**) (77.0 mg, 0.20 mmol, 1.0 equiv)[6] and KO*t*Bu (54.0 mg, 0.48 mmol, 2.4 equiv). The Schlenk tube was sealed with a rubber septum and taken out of the glovebox. Then, a solution of the obtained β-chiral homoallylboronic ester in THF (0.50 mL, 0.4 M) was added. The reaction mixture was heated to 80 °C and stirred for 2 h before being cooled to room temperature. Boc_2O (87.0 mg, 0.40 mmol, 2.0 equiv) was added and the reaction mixture was stirred for 2 h at rt. After that, the reaction mixture was diluted with MTBE and quenched by the addition of water. The phases were separated, and the aqueous phase was extracted with MTBE (3x). The combined organic layers were dried over Na_2SO_4 and concentrated *in vacuo*. The crude product was purified by flash column chromatography (PE:MTBE 98:2 → 95:5) to afford β-chiral homoallylic Boc-protected amine **17** (31.8 mg, 0.06 mmol, 30% o3s, dr ≥ 19:1) as a colorless oil.

^{1}H-NMR (400 MHz, C_6D_6): δ = 5.37 (m_c, 1H), 4.36 (brs, 1H), 4.11–4.01 (m, 2H), 3.57–3.51 (m, 1H), 3.41 (m_c, 1H), 2.85 (m_c, 1H), 2.02 (m_c, 1H), 1.73–1.62 (m, 2H), 1.49 (s, 9H), 1.42 (s, 3H), 1.01 (s, 9H), 1.00 (s, 9H), 0.94–0.91 (m, 6H), 0.88 (d, 3H, J = 7.0 Hz), 0.10 (s, 6H), 0.08 (s, 3H), 0.07 (s, 3H) ppm;

$^{13}C\{^1H\}$-NMR (101 MHz, C_6D_6): δ = 155.7, 136.4, 129.1, 78.6, 77.3, 59.7, 51.0, 40.4, 40.3, 29.1, 28.6, 26.3, 26.2, 23.1, 18.6, 18.5, 17.7, 13.1, 12.3, –3.8, –4.1, –5.0, –5.0 ppm;

HRMS (ESI) m/z: calcd for $C_{29}H_{61}NO_4Si_2Na$ $[M+Na]^+$ 566.4037, found: 566.4042;

R_f = 0.4 (PE:MTBE 98:2, vanillin);

$[\alpha]_D^{20}$ = +6.9 (*c* 1.7, $CHCl_3$).

β-Chiral γ,δ-unsaturated aldehyde 18

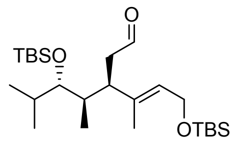

18
$C_{25}H_{52}O_3Si_2$
456.86

To a stirred solution of literature known TIB ester **4** (143 mg, 0.30 mmol, 1.5 equiv)[1] and TMEDA (0.05 mL, 0.30 mmol, 1.5 equiv) in Et_2O (1.5 mL, 0.2 M) at −78 °C was added *s*BuLi (1.3 M in hexanes, 0.22 mL, 0.28 mmol, 1.4 equiv). The reaction mixture was stirred for 5 h at that temperature before a solution of literature known vinyl boronic ester **7** (62.2 mg, 0.20 mmol, 1.0 equiv)[1, 2] in Et_2O (0.40 mL, 0.5 M) was added. After stirring for further 3 h at −78 °C, the reaction mixture was warmed to 45 °C and stirred overnight. The reaction mixture was cooled to rt, sat. aq. NH_4Cl was added, and the biphasic mixture was stirred for 15 min. The phases were separated, the organic layer was washed with sat. aq. NH_4Cl (3x) and the combined aqueous phases were extracted with MTBE (3x). The combined organic phases were dried over Na_2SO_4, concentrated *in vacuo* and the crude material was purified by a short flash column chromatography (PE:MTBE 95:5) to remove TIBOH.

The crude product was dissolved in Et_2O (1.0 mL, 0.2 M), treated with $ClCH_2I$ (0.06 mL, 0.80 mmol, 4.0 equiv) and cooled to –95 °C. *n*BuLi (1.6 M in hexanes, 0.41 mL, 0.66 mmol, 3.3 equiv) was added dropwise (0.05 mL/min) to the reaction mixture which was then held for 5 min at this temperature. The vessel containing the reaction mixture was then transferred to a –78 °C cooling bath. The reaction mixture was stirred for 3 h at –78 °C, before being warmed to room temperature and stirred overnight. The obtained reaction suspension was filtered over a plug of silica (Et_2O) and the filtrate was concentrated under reduced pressure.

To a stirred solution of the obtained β-chiral homoallylboronic ester in THF (1.7 mL, 0.12 M) and CH_2Cl_2 (0.33 mL, 0.6 M) at −78 °C was added *n*BuLi (1.6 M in hexanes, 0.25 mL, 0.40 mmol, 2.0 equiv, 0.05 mL/min). The reaction mixture was warmed to rt and stirred overnight. After that, the reaction mixture was cooled to 0 °C and sodium percarbonate (53.0 mg, 0.34 mmol, 1.7 equiv) was added. Then, the reaction mixture was warmed to rt and stirred for 2 h at that temperature before being diluted with MTBE and quenched by the addition of sat. aq. $Na_2S_2O_3$. The phases were separated, and the aqueous phase was extracted with MTBE (3x). The combined organic layers were dried over Na_2SO_4 and concentrated *in vacuo*. The crude product was purified by flash column chromatography (PE:MTBE 95:5 → 9:1) to afford β-chiral γ,δ-unsaturated

aldehyde **18** (21.3 mg, 0.05 mmol, 25% o3s, dr ≥ 19:1, unstable compound, fast decomposition) as a yellow oil.

^{1}H-NMR (400 MHz, C_6D_6): δ = 9.40 (dd, 1H, J = 3.4, 1.1 Hz), 5.38 (t, 1H, J = 6.1 Hz), 4.09 (m$_c$, 2H), 3.35 (dd, 1H, J = 5.2, 2.1 Hz), 2.55–2.49 (m, 1H), 2.12–2.00 (m, 2H), 1.74 (m$_c$, 1H), 1.68–1.60 (m, 1H), 1.47 (s, 3H), 1.00 (s, 9H), 1.00 (s, 9H), 0.96 (d, 3H, J = 6.8 Hz), 0.94 (d, 3H, J = 6.7 Hz), 0.75 (d, 3H, J = 7.1 Hz), 0.08 (s, 6H), 0.06 (s, 3H), 0.04 (s, 3H) ppm;

^{13}C{^{1}H}-NMR (101 MHz, C_6D_6): δ = 200.7, 136.9, 127.9 (covered by C_6D_6), 77.5, 59.9, 44.6, 43.9, 41.0, 29.5, 26.3, 26.1, 22.6, 18.6, 18.5, 17.6, 14.2, 12.3, –3.6, –3.9, –5.0, –5.1 ppm;

HRMS (ESI) m/z: calcd for $C_{25}H_{52}O_3Si_2Na$ $[M+Na]^+$ 479.3353, found: 479.3344;

R$_f$ = 0.5 (PE:MTBE 95:5, vanillin);

$[\alpha]_D^{20}$ = −4.4 (*c* 2.0, $CHCl_3$).

β-Chiral vinyl bromide 19

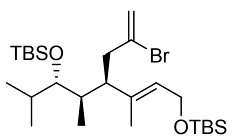

19
$C_{26}H_{53}BrO_2Si_2$
533.78

To a stirred solution of literature known TIB ester **4** (143 mg, 0.30 mmol, 1.5 equiv)[1] and TMEDA (0.05 mL, 0.30 mmol, 1.5 equiv) in Et_2O (1.5 mL, 0.2 M) at −78 °C was added *s*BuLi (1.3 M in hexanes, 0.22 mL, 0.28 mmol, 1.4 equiv). The reaction mixture was stirred for 5 h at that temperature before a solution of literature known vinyl boronic ester **7** (62.2 mg, 0.20 mmol, 1.0 equiv)[1, 2] in Et_2O (0.40 mL, 0.5 M) was added. After stirring for further 3 h at −78 °C, the reaction mixture was warmed to 45 °C and stirred overnight. The reaction mixture was cooled to rt, sat. aq. NH_4Cl was added, and the biphasic mixture was stirred for 15 min. The phases were separated, the organic layer was washed with sat. aq. NH_4Cl (3x) and the combined aqueous phases were extracted with MTBE (3x). The combined organic phases were dried over Na_2SO_4, concentrated *in vacuo* and the crude material was purified by a short flash column chromatography (PE:MTBE 95:5) to remove TIBOH.
The crude product was dissolved in Et_2O (1.0 mL, 0.2 M), treated with $ClCH_2I$ (0.06 mL, 0.80 mmol, 4.0 equiv) and cooled to –95 °C. *n*BuLi (1.6 M in hexanes, 0.41 mL, 0.66 mmol, 3.3 equiv) was added dropwise (0.05 mL/min) to the reaction mixture which was then held for 5 min at this temperature. The vessel containing the reaction mixture was then transferred to a –78 °C cooling bath. The reaction mixture was stirred for 3 h at –78 °C, before being warmed to room temperature and stirred overnight. The obtained reaction suspension was filtered over a plug of silica (Et_2O) and the filtrate was concentrated under reduced pressure.
Diisopropylamine (0.05 mL, 0.32 mmol, 1.6 equiv) was dissolved in THF (0.20 mL). After cooling to −78 °C *n*BuLi (1.6 M in hexanes, 0.21 mL, 0.34 mmol, 1.7 equiv) was added dropwise and the reaction mixture was stirred for 15 min at −78 °C and then for 5 min at rt.

Simultaneously the obtained β-chiral homoallylboronic ester was dissolved in THF (1.0 mL, 0.2 M) and cooled to −95 °C. Vinyl bromide (1.0 M in THF, 0.40 mL, 0.40 mmol, 2.0 equiv) was added and then the freshly prepared LDA solution was added (60 µL/min, rinsed once with 0.15 mL THF). After stirring for 1 h at this temperature iodine (203 mg, 0.80 mmol, 4.0 equiv) was added in two portions over a period of 10 min. The reaction mixture was then warmed to −78 °C and MeOH (2.7 mL, 1.60 mmol, 8.0 equiv, 0.15 mL/min) was added to the dark red solution and the reaction mixture was stirred for 30 min. Then a suspension of NaOMe (86.0 mg, 1.60 mmol, 8.0 equiv, 0.5 mL/min) in MeOH (3.2 mL) was added and the reaction mixture was warmed to rt and stirred for 1 h at this temperature. MTBE and sat. aq. $Na_2S_2O_3$ were added. The phases were separated, and the aqueous phase was extracted with MTBE (3x). The combined organic phases were washed with sat. aq. NaCl and dried over Na_2SO_4, concentrated *in vacuo* and the crude material was purified by flash column chromatography (PE:MTBE 95:5) to afford β-chiral vinyl bromide **19** (17.8 mg, 0.03 mmol, 15% o3s, dr ≥ 19:1) as a pale yellow oil.

^{1}H-NMR (400 MHz, C_6D_6): δ = 5.57 (m_c, 1H), 5.30 (s, 1H), 5.21 (s, 1H), 4.16 (m_c, 2H), 3.50 (dd, 1H, J = 4.2, 2.2 Hz), 2.57–2.46 (m, 2H), 2.06 (dd, 1H, J = 14.3, 11.1 Hz), 1.89 (m_c, 1H), 1.70 (m_c, 1H), 1.44 (s, 3H), 1.03 (s, 9H), 1.01–0.99 (m, 12H), 0.98 (d, 3H, J = 6.7 Hz), 0.93 (d, 3H, J = 7.1 Hz), 0.11–0.10 (m, 12H) ppm;

^{13}C{^{1}H}-NMR (101 MHz, C_6D_6): δ = 135.3, 134.1, 129.4, 117.7, 77.6, 59.9, 49.7, 42.4, 41.9, 29.0, 26.3, 26.2, 23.3, 18.6, 18.6, 18.1, 12.9, 12.6, –3.8, –4.1, –5.0, –5.1 ppm;

HRMS (ESI) m/z: calcd for $C_{26}H_{53}BrO_2Si_2Na$ $[M+Na]^+$ 555.2665, found: 555.2654;

R_f = 0.8 (PE:MTBE 100:1, vanillin);

$[\alpha]_D^{20}$ = +10.9 (*c* 1.9, $CHCl_3$).

β-Chiral 1,5-diene 20

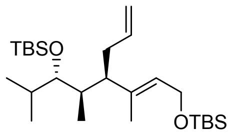

20
$C_{26}H_{54}O_2Si_2$
454.89

To a stirred solution of literature known TIB ester **4** (143 mg, 0.30 mmol, 1.5 equiv)[1] and TMEDA (0.05 mL, 0.30 mmol, 1.5 equiv) in Et_2O (1.5 mL, 0.2 M) at −78 °C was added *s*BuLi (1.3 M in hexanes, 0.22 mL, 0.28 mmol, 1.4 equiv). The reaction mixture was stirred for 5 h at that temperature before a solution of literature known vinyl boronic ester **7** (62.2 mg, 0.20 mmol, 1.0 equiv)[1, 2] in Et_2O (0.40 mL, 0.5 M) was added. After stirring for further 3 h at −78 °C, the reaction mixture was warmed to 45 °C and stirred overnight. The reaction mixture was cooled to rt, sat. aq. NH_4Cl was added, and the biphasic mixture was stirred for 15 min. The phases were separated, the organic layer was washed with sat. aq. NH_4Cl (3x) and the combined aqueous phases were extracted with MTBE (3x). The combined organic phases were

dried over Na_2SO_4, concentrated *in vacuo* and the crude material was purified by a short flash column chromatography (PE:MTBE 95:5) to remove TIBOH.

The crude product was dissolved in Et_2O (1.0 mL, 0.2 M), treated with $ClCH_2I$ (0.06 mL, 0.80 mmol, 4.0 equiv) and cooled to –95 °C. *n*BuLi (1.6 M in hexanes, 0.41 mL, 0.66 mmol, 3.3 equiv) was added dropwise (0.05 mL/min) to the reaction mixture which was then held for 5 min at this temperature. The vessel containing the reaction mixture was then transferred to a –78 °C cooling bath. The reaction mixture was stirred for 3 h at –78 °C, before being warmed to room temperature and stirred overnight. The obtained reaction suspension was filtered over a plug of silica (Et_2O) and the filtrate was concentrated under reduced pressure.

To a stirred solution of the obtained β-chiral homoallylboronic ester in THF (2.0 mL, 0.1 M) at rt was added vinylmagnesium bromide (1.0 M in THF, 0.80 mL, 0.80 mmol, 4.0 equiv). The reaction mixture was stirred for 30 min at rt before being cooled to −78 °C. Then, iodine (203 mg, 0.80 mmol, 4.0 equiv) was added in two portions over a period of 10 min followed by the dropwise addition of MeOH (2.7 mL, 1.60 mmol, 8.0 equiv, 0.15 mL/min). The reaction mixture was then stirred for 30 min at –78 °C before a suspension of NaOMe (86.0 mg, 1.60 mmol, 8.0 equiv, 0.5 mL/min) in MeOH (3.2 mL) was added. The resulting reaction mixture was warmed to rt and stirred for 1 h at this temperature. MTBE and sat. aq. $Na_2S_2O_3$ were added. The phases were separated, and the aqueous phase was extracted with MTBE (3x). The combined organic phases were washed with sat. aq. NaCl and dried over Na_2SO_4, concentrated *in vacuo* and the crude material was purified by flash column chromatography (PE:MTBE 95:5) to afford β-chiral 1,5-diene **20** (28.1 mg, 0.06 mmol, 30% o3s, dr ≥ 19:1) as a pale yellow oil.

^{1}H-NMR (400 MHz, C_6D_6): δ = 5.78–5.68 (m, 1H), 5.42 (m_c, 1H), 5.04–4.99 (m, 2H), 4.19 (m_c, 2H), 3.52 (dd, 1H, J = 4.6, 1.8 Hz), 2.31–2.24 (m, 1H), 2.05–1.99 (m, 1H), 1.92–1.69 (m, 3H), 1.49 (s, 3H), 1.04 (s, 9H), 1.02–0.99 (m, 15H), 0.95 (d, 3H, J = 7.0 Hz), 0.11 (s, 3H), 0.11 (s, 3H), 0.10 (s, 6H) ppm;

^{13}C{^{1}H}-NMR (101 MHz, C_6D_6): δ = 137.9, 137.0, 127.9 (covered by C_6D_6), 115.1, 77.4, 60.0, 51.1, 42.3, 34.8, 29.0, 26.3, 26.2, 23.3, 18.6, 18.6, 17.8, 13.3, 12.5, –3.7, –4.1, –5.0, –5.0 ppm;

HRMS (ESI) m/z: calcd for $C_{26}H_{53}BrO_2Si_2Na$ $[M+Na]^+$ 555.2665, found: 555.2654;

R_f = 0.8 (PE:MTBE 100:1, vanillin);

$[\alpha]_D^{20}$ = +10.9 (*c* 1.9, $CHCl_3$).

β-Chiral vinyl carbamate 21

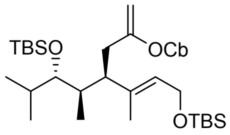

21
$C_{33}H_{67}NO_4Si_2$
598.07

To a stirred solution of literature known TIB ester **4** (143 mg, 0.30 mmol, 1.5 equiv)[1] and TMEDA (0.05 mL, 0.30 mmol, 1.5 equiv) in Et_2O (1.5 mL, 0.2 M) at −78 °C was added *s*BuLi (1.3 M in hexanes, 0.22 mL, 0.28 mmol, 1.4 equiv). The reaction mixture was stirred for 5 h at that temperature before a solution of literature known vinyl boronic ester **7** (62.2 mg, 0.20 mmol, 1.0 equiv)[1, 2] in Et_2O (0.40 mL, 0.5 M) was added. After stirring for further 3 h at −78 °C, the reaction mixture was warmed to 45 °C and stirred overnight. The reaction mixture was cooled to rt, sat. aq. NH_4Cl was added, and the biphasic mixture was stirred for 15 min. The phases were separated, the organic layer was washed with sat. aq. NH_4Cl (3x) and the combined aqueous phases were extracted with MTBE (3x). The combined organic phases were dried over Na_2SO_4, concentrated *in vacuo* and the crude material was purified by a short flash column chromatography (PE:MTBE 95:5) to remove TIBOH.

The crude product was dissolved in Et_2O (1.0 mL, 0.2 M), treated with $ClCH_2I$ (0.06 mL, 0.80 mmol, 4.0 equiv) and cooled to –95 °C. *n*BuLi (1.6 M in hexanes, 0.41 mL, 0.66 mmol, 3.3 equiv) was added dropwise (0.05 mL/min) to the reaction mixture which was then held for 5 min at this temperature. The vessel containing the reaction mixture was then transferred to a –78 °C cooling bath. The reaction mixture was stirred for 3 h at –78 °C, before being warmed to room temperature and stirred overnight. The obtained reaction suspension was filtered over a plug of silica (Et_2O) and the filtrate was concentrated under reduced pressure.

Diisopropylamine (0.05 mL, 0.32 mmol, 1.6 equiv) was dissolved in THF (0.20 mL). After cooling to −78 °C *n*BuLi (1.6 M in hexanes, 0.21 mL, 0.34 mmol, 1.7 equiv) was added dropwise and the reaction mixture was stirred for 15 min at −78 °C and then for 5 min at rt. The freshly prepared LDA solution was then added to a stirred solution of vinyl carbamate (55.0 mg, 0.32 mmol, 1.6 equiv)[7] in THF (2.0 mL) at –78 °C. After stirring for 10 min at this temperature a solution of the obtained β-chiral homoallylboronic ester in THF (1.0 mL, 0.2 M) was added. The reaction mixture was stirred for 30 min at –78 °C before being warmed to 0 °C and stirring was continued for 30 min at this temperature. After cooling the reaction mixture back to –78 °C, iodine (81.0 mg, 0.32 mmol, 1.6 equiv) was added in two portions over a period of 5 min. MeOH (2.7 mL, 1.60 mmol, 8.0 equiv, 0.15 mL/min) was added dropwise and the reaction mixture was warmed to rt and stirred for 30 min at that temperature. MTBE and sat. aq. $Na_2S_2O_3$ were added. The phases were separated, and the aqueous phase was extracted with MTBE (3x). The combined organic phases were washed with sat. aq. NaCl and dried over Na_2SO_4, concentrated *in vacuo* and the crude material was purified by flash column chromatography (PE:MTBE 95:5) to afford β-chiral vinyl carbamate **21** (74.5 mg, 0.12 mmol, 60% o3s, dr ≥ 19:1) as a colorless oil.

^{1}H-NMR (400 MHz, C_6D_6): δ = 5.57 (t, 1H, J = 6.1 Hz), 4.89 (s, 1H), 4.54 (s, 1H), 4.23 (m$_c$, 2H), 3.79 (brs, 2H), 3.57 (dd, 1H, J = 4.3, 2.0 Hz), 2.95 (dd, 1H, J = 14.9, 3.1 Hz), 2.43–2.37

(m, 1H), 2.15–2.08 (m, 1H), 1.97 (m_c, 1H), 1.77 (m_c, 1H), 1.56 (s, 3H), 1.14 (brs, 12H), 1.06–1.03 (m, 18H), 1.01 (s, 9H), 0.13 (s, 3H), 0.12 (s, 3H), 0.10 (s, 6H) ppm;

^{13}C{^{1}H}-NMR (101 MHz, C_6D_6): δ = 156.1, 152.6, 136.5, 128.7, 101.1, 77.5, 60.1, 49.0, 46.3 (brs), 42.2, 35.2, 29.1, 26.3, 26.2, 23.3, 21.0 (brs), 18.6, 18.6, 18.0, 13.0, 12.7, –3.7, –4.1, –5.0, –5.0 ppm;

HRMS (ESI) m/z: calcd for $C_{33}H_{67}NO_4Si_2Na$ [M+Na]$^+$ 620.4506, found: 620.4490;

R$_f$ = 0.4 (PE:MTBE 95:5, vanillin);

$[\alpha]_D^{20}$ = +3.3 (*c* 1.3, $CHCl_3$).

β-Chiral 1,5-enyne 22

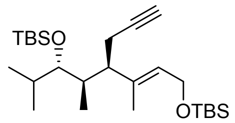

22
$C_{26}H_{52}O_2Si_2$
452.87

To a stirred solution of β-chiral vinyl carbamate **21** (30.0 mg, 50.1 µmol, 1.0 equiv) in Et_2O (0.50 mL, 0.1 M) was added *t*BuLi (1.7 M in pentane, 32 µL, 55.2 µmol, 1.1 equiv) dropwise at −78 °C. After warming to 0 °C the reaction mixture was stirred for 1 h at this temperature. Sat. aq. NH_4Cl and Et_2O were added, and the organic layer was separated. The aqueous layer was extracted with Et_2O (3x). The combined organic layers were washed with sat. aq. NaCl and dried over Na_2SO_4. The solvent was removed *in vacuo* and the crude material was purified by flash column chromatography (PE:MTBE 100:1) to afford β-chiral 1,5-enyne **22** (13.7 mg, 30.3 µmol, 60%, dr ≥ 19:1) as a colorless oil.

^{1}H-NMR (400 MHz, C_6D_6): δ = 5.54 (t, 1H, J = 6.1 Hz), 4.25 (m_c, 2H), 3.48 (dd, 1H, J = 4.9, 2.1 Hz), 2.27–2.12 (m, 2H), 2.08–2.01 (m, 1H), 1.84–1.75 (m, 2H), 1.72 (m_c, 1H), 1.60 (s, 3H), 1.02 (s, 9H), 1.02 (s, 9H), 0.97 (d, 3H, J = 6.9 Hz), 0.94 (d, 3H, J = 6.7 Hz), 0.83 (d, 3H, J = 7.1 Hz), 0.13 (s, 3H), 0.12 (s, 6H), 0.10 (s, 3H) ppm;

^{13}C{^{1}H}-NMR (101 MHz, C_6D_6): δ = 136.8, 127.9 (covered by C_6D_6), 83.2, 77.4, 70.0, 60.2, 49.2, 41.3, 29.3, 26.3, 26.2, 23.0, 20.5, 18.6, 18.6, 17.6, 14.1, 12.1, –3.7, –4.0, –4.9, –5.0 ppm;

HRMS (ESI) m/z: calcd for $C_{26}H_{52}O_2Si_2Na$ [M+Na]$^+$ 475.3404, found: 475.3405;

R$_f$ = 0.5 (PE:MTBE 100:1, vanillin);

$[\alpha]_D^{20}$ = −5.0 (*c* 1.0, $CHCl_3$).

5 References

[1] Linne, Y.; Birkner, M.; Flormann, J.; Lücke, D.; Becker, J. A.; Kalesse, M. Sparteine-Free, Highly Stereoselective Construction of Complex Allylic Alcohols Using 1,2-Metallate Rearrangements. *JACS Au* **2023**, *3*, 1695−1710.

[2] Hesse, M. J.; Butts, C. P.; Willis, C. L.; Aggarwal, V. K. Diastereodivergent Synthesis of Trisubstituted Alkenes through Protodeboronation of Allylic Boronic Esters: Application to the Synthesis of the Californian Red Scale Beetle Pheromone. *Angew. Chem. Int. Ed.* **2012**, *51*, 12444–12448.

[3] Hoppe, D.; Hintze, F.; Tebben, P.; Paetow, M.; Ahrens, H.; Schwerdtfeger, J.; Sommerfeld, P.; Haller, J.; Guarnieri, W.; Kolczewski, S.; Hense, T.; Hoppe, I. Enantioselective synthesis via sparteine-induced asymmetric deprotonation. *Pure & Appl. Chem.* **1994**, *66*, 1479–1486.

[4] Würthwein, E.-U.; Hoppe, D. Enantioselective Lithiation of O-Alkyl and O-Alk-2-enyl Carbamates in the Presence of (−)-Sparteine and (−)-α-Isosparteine. A Theoretical Study. *J. Org. Chem.* ***2005**, 70,* 4443–4451.

[5] (a) Blair, D. J.; Tanini, D.; Bateman, J. M.; Scott, H. K.; Myers, E. L.; Aggarwal, V. K. Selective uni- and bidirectional homologation of diborylmethane. *Chem. Sci.* **2017**, *8*, 2898–2903; (b) Burns, M.; Essafi, S.; Bame, J. R.; Bull, S. P.; Webster, M. P.; Balieu, S.; Dale, J. W.; Butts, C. P.; Harvey, J. N.; Aggarwal, V. K. Assembly-line synthesis of organic molecules with tailored shapes. *Nature* **2014**, *513*, 183−188; (c) Linne, Y.; Schönwald, A.; Weißbach, S.; Kalesse, M. Desymmetrization of C_2-Symmetric Bis(Boronic Esters) by Zweifel Olefinations. *Chem. Eur. J.* **2020**, *26*, 7998–8002.

[6] Liu, X.; Zhu, Q.; Chen, D.; Wang, L.; Jin, L.; Liu, C. Aminoazanium of DABCO: An Amination Reagent for Alkyl and Aryl Pinacol Boronates. *Angew. Chem. Int. Ed.* **2020**, *59*, 2745–2749.

[7] Prepared on multigram scale according to (a) Fournier, A. M.; Clayden, J. Tertiary Alcohols by Tandem β-Carbolithiation and N→C Aryl Migration in Enol Carbamates. *Org. Lett.* **2012**, *14*, 142–145; (b) Webb, N. J.; Marsden, S. P.; Raw, S. A. Rhodium(III)-Catalyzed C–H Activation/Annulation with Vinyl Esters as an Acetylene Equivalent. *Org. Lett.* **2014**, *16*, 4718–4721.

6 Spectra

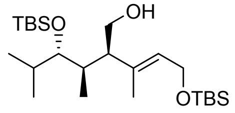

9a

^{1}H NMR (400 MHz, C_6D_6)

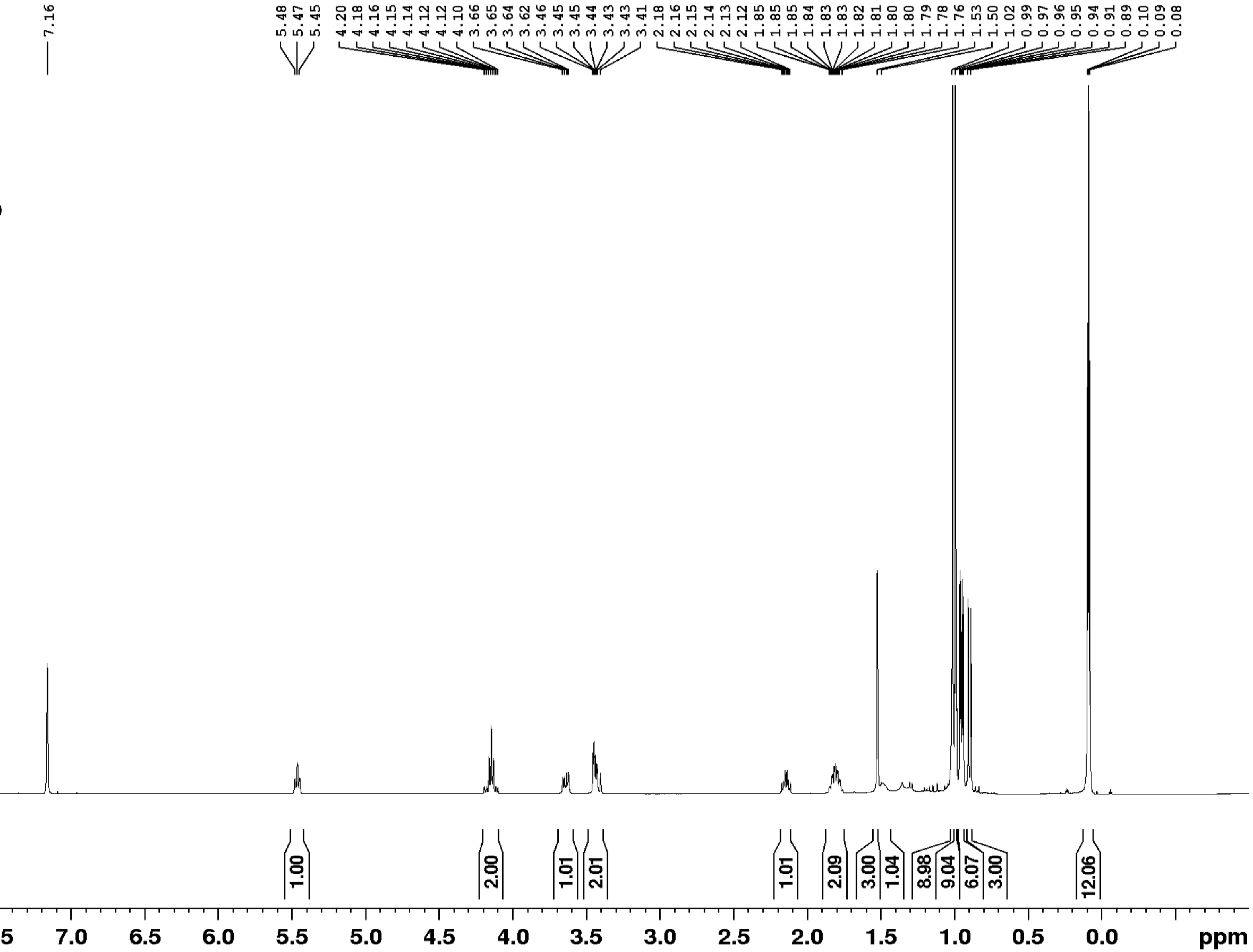

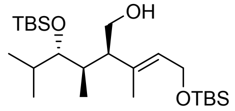

9a

$^{13}C\{^1H\}$ NMR (101 MHz, C_6D_6)

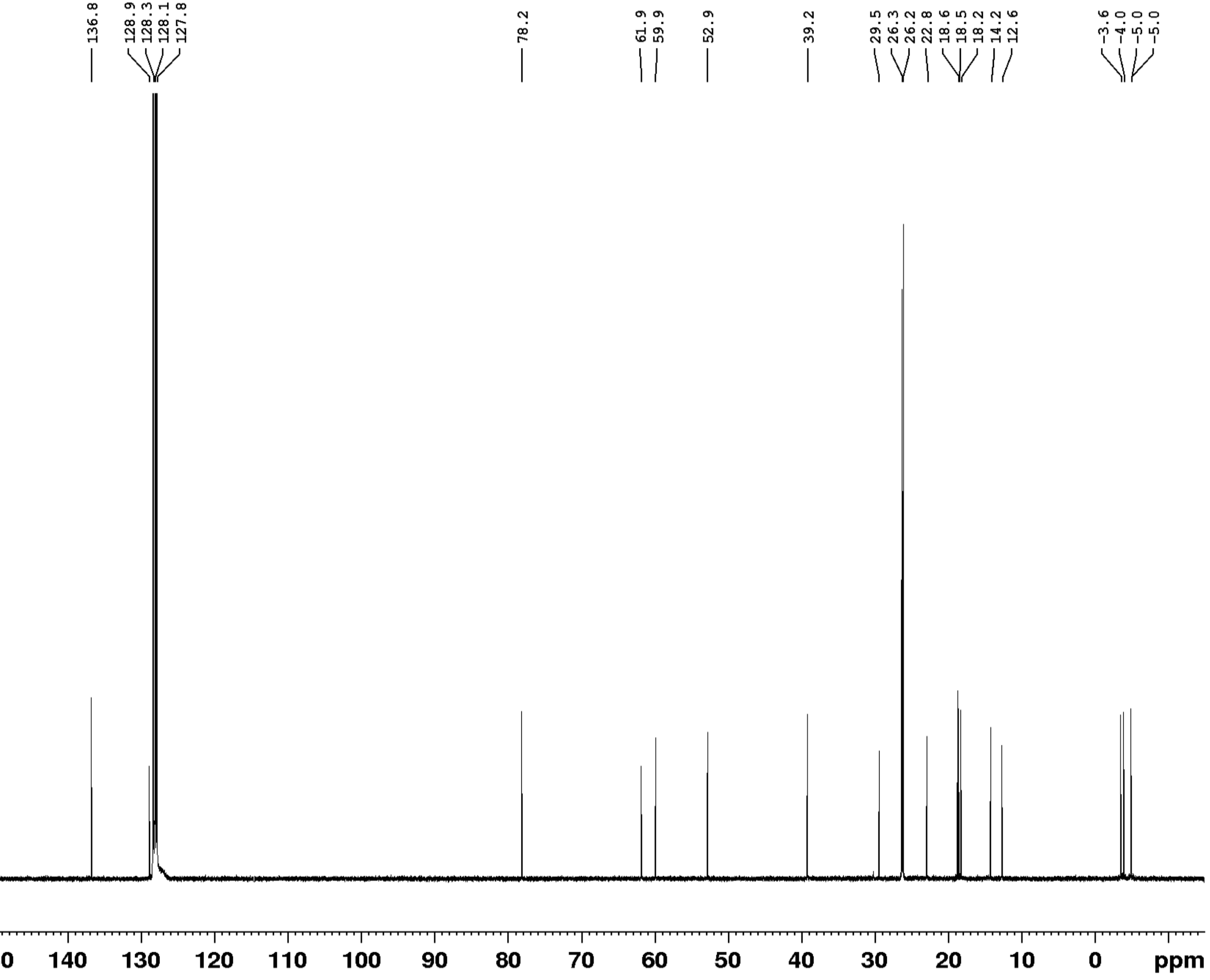

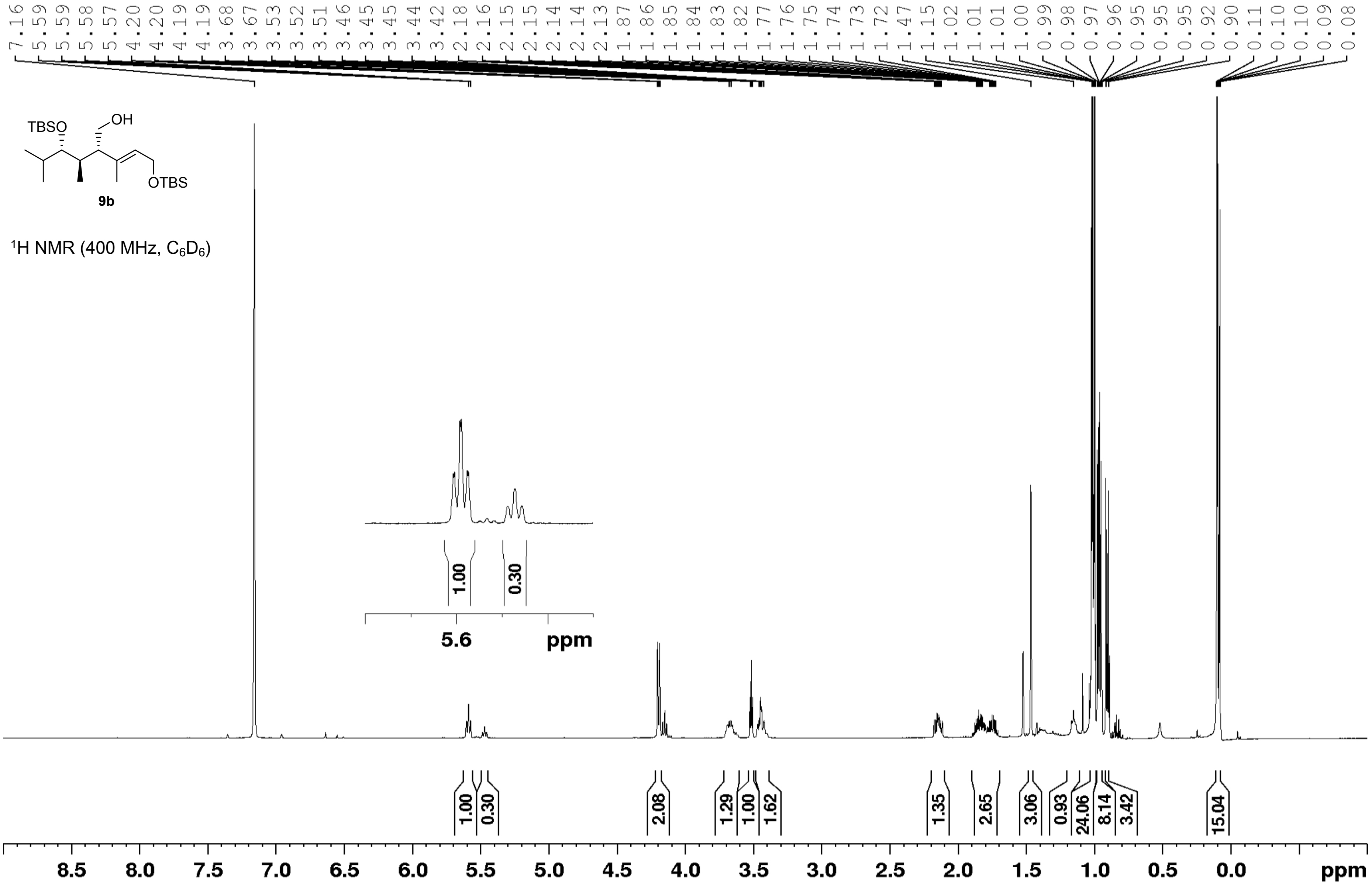
7.16 5.59 5.59 5.58 5.57 4.20 4.20 4.19 4.19 3.68 3.67 3.53 3.52 3.51 3.46 3.45 3.45 3.44 3.42 2.18 2.16 2.15 2.15 2.14 2.14 2.13 1.87 1.86 1.85 1.84 1.83 1.82 1.77 1.76 1.75 1.74 1.73 1.72 1.47 1.15 1.02 1.01 1.01 1.00 0.99 0.98 0.97 0.96 0.95 0.95 0.95 0.92 0.90 0.11 0.10 0.10 0.09 0.08
TBSO
OH
OTBS
9b
1H NMR (400 MHz, C6D6)
1.00
0.30
5.6
ppm
1.00
0.30
2.08
1.29
1.00
1.62
1.35
2.65
3.06
0.93
24.06
8.14
3.42
15.04
8.5 8.0 7.5 7.0 6.5 6.0 5.5 5.0 4.5 4.0 3.5 3.0 2.5 2.0 1.5 1.0 0.5 0.0 ppm

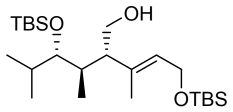

9b

$^{13}C\{^1H\}$ NMR (101 MHz, C_6D_6)

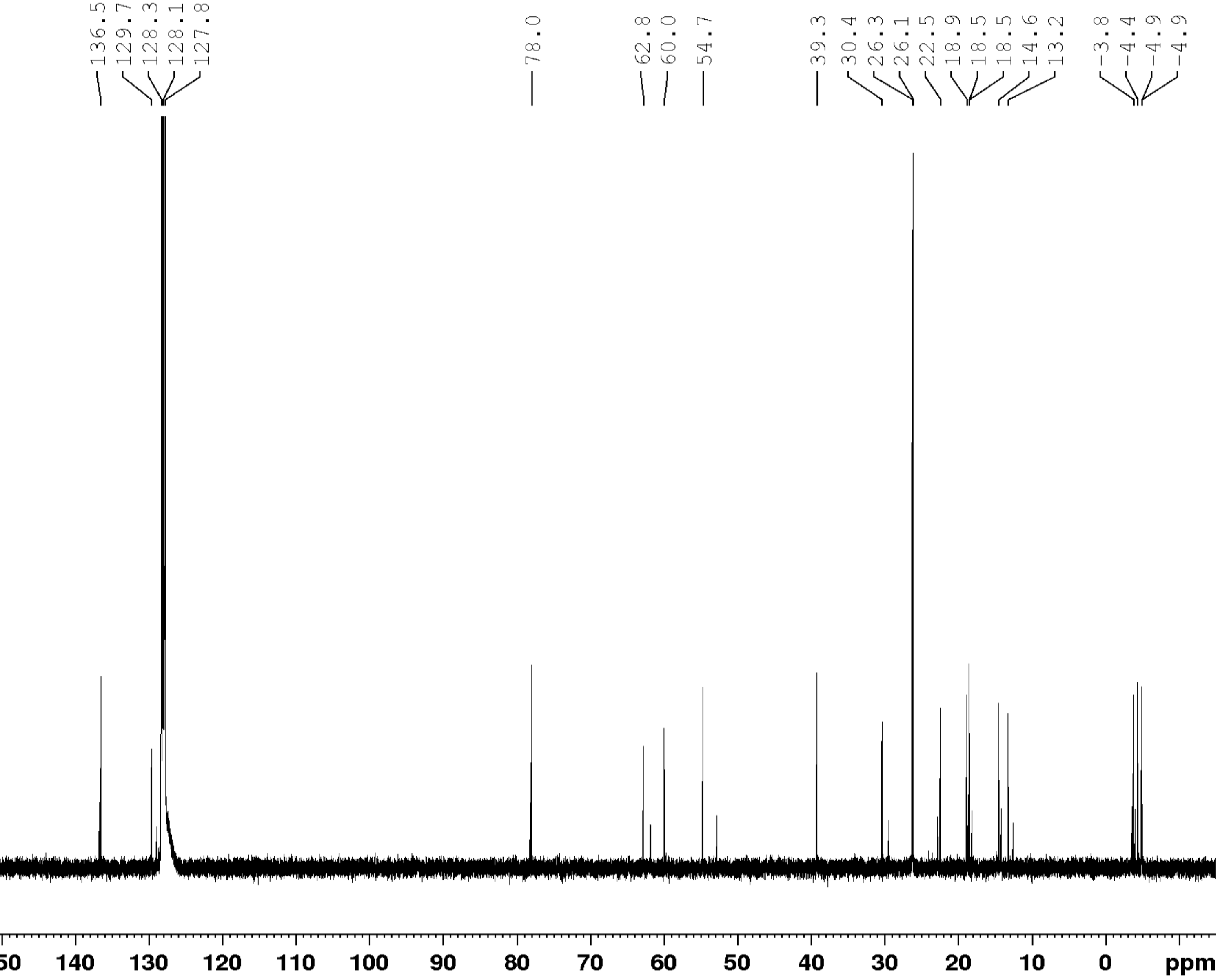

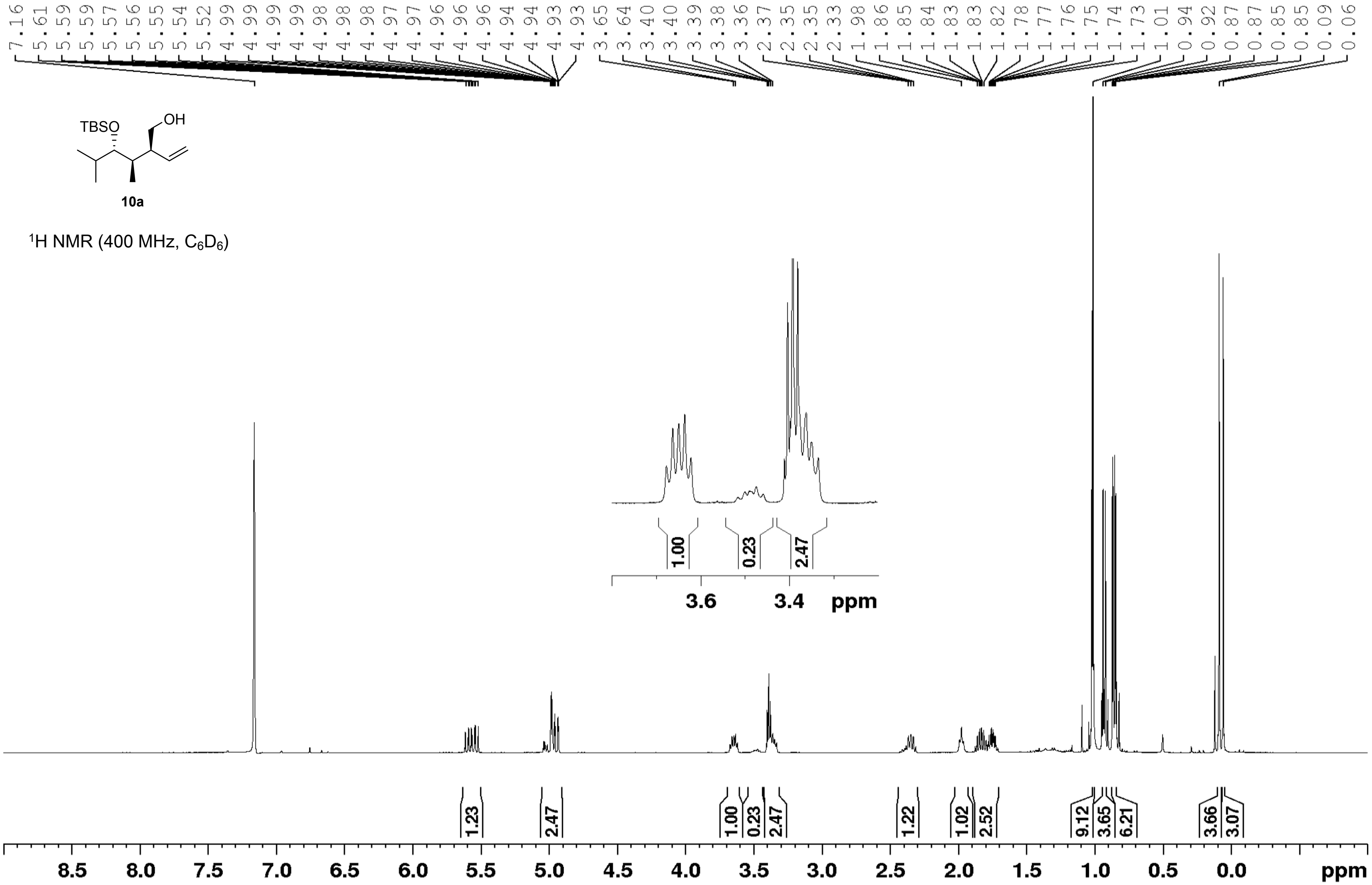

7.16 5.61 5.59 5.59 5.57 5.56 5.55 5.54 5.52 4.99 4.99 4.99 4.99 4.98 4.98 4.98 4.97 4.97 4.96 4.96 4.96 4.94 4.94 4.93 4.93 3.65 3.64 3.40 3.40 3.39 3.38 3.36 2.37 2.35 2.35 2.33 1.98 1.86 1.85 1.84 1.83 1.83 1.82 1.78 1.77 1.76 1.75 1.74 1.73 1.01 0.94 0.92 0.87 0.87 0.85 0.85 0.09 0.06
TBSO
OH
10a
¹H NMR (400 MHz, C6D6)
1.00
0.23
2.47
3.6
3.4
ppm
1.23
2.47
1.00
0.23
2.47
1.22
1.02
2.52
9.12
3.65
6.21
3.66
3.07
8.5 8.0 7.5 7.0 6.5 6.0 5.5 5.0 4.5 4.0 3.5 3.0 2.5 2.0 1.5 1.0 0.5 0.0
ppm

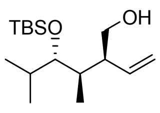

10a

$^{13}C\{^{1}H\}$ NMR (101 MHz, C_6D_6)

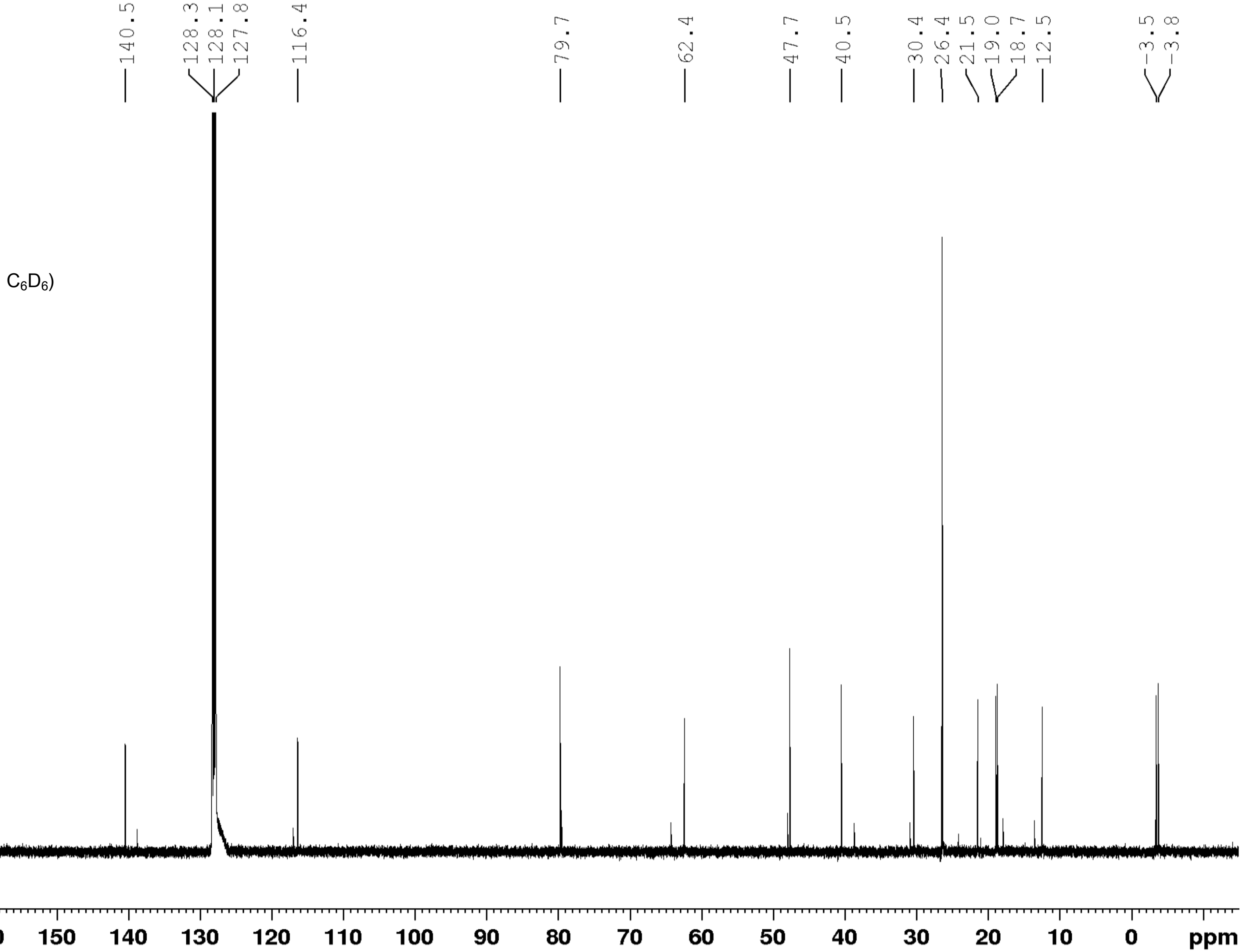

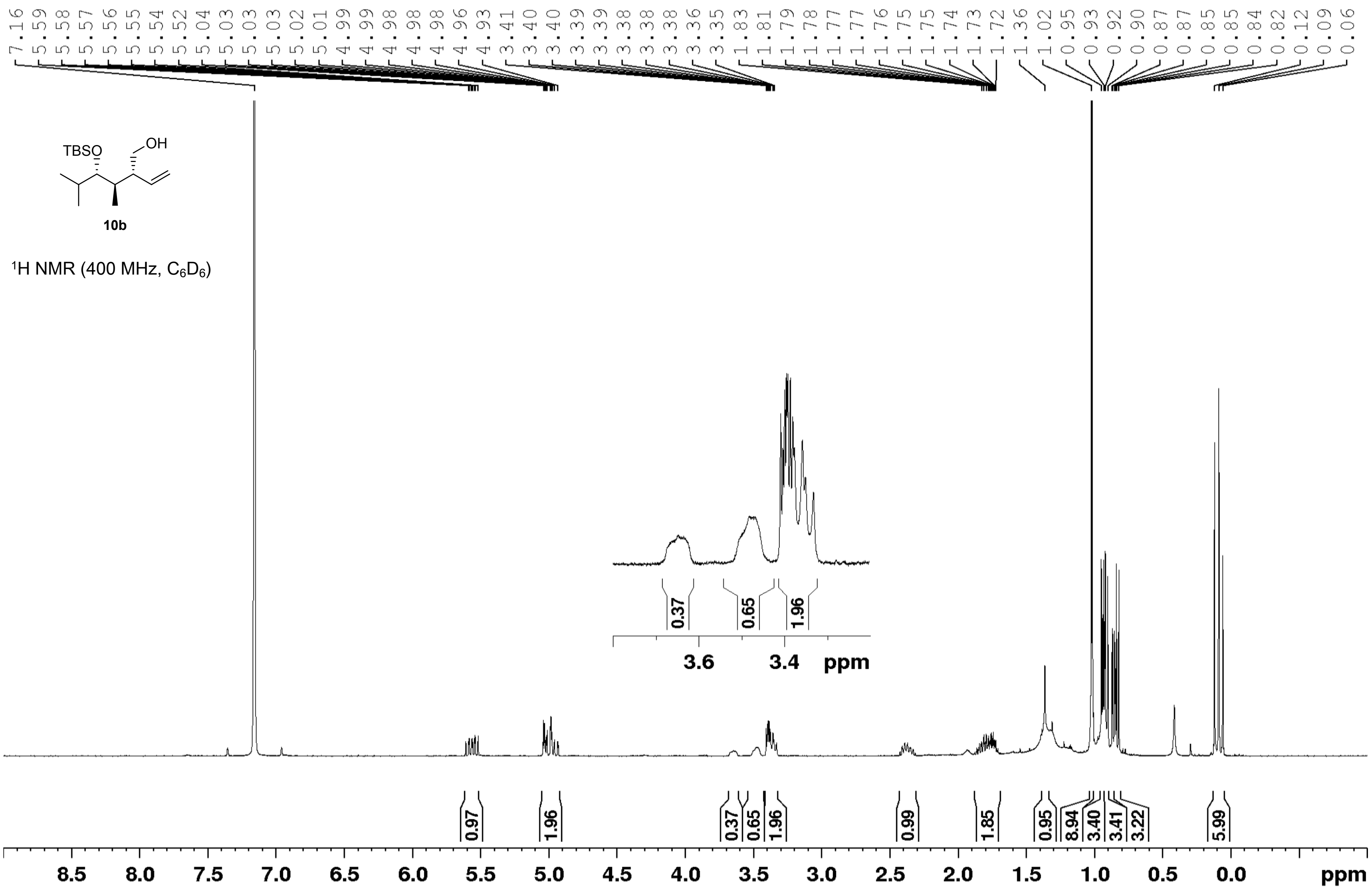

TBSO
OH
10b
¹H NMR (400 MHz, C6D6)
7.16 5.59 5.58 5.57 5.56 5.55 5.54 5.52 5.04 5.03 5.03 5.03 5.02 5.01 4.99 4.99 4.98 4.98 4.98 4.96 4.93 3.41 3.40 3.40 3.39 3.39 3.38 3.38 3.38 3.36 3.35 1.83 1.81 1.79 1.78 1.77 1.77 1.76 1.75 1.75 1.74 1.73 1.72 1.36 1.02 0.95 0.93 0.92 0.90 0.87 0.87 0.85 0.85 0.84 0.82 0.12 0.09 0.06
0.37
0.65
1.96
3.6
3.4
ppm
0.97 1.96 0.37 0.65 1.96 0.99 1.85 0.95 8.94 3.40 3.41 3.22 5.99
8.5 8.0 7.5 7.0 6.5 6.0 5.5 5.0 4.5 4.0 3.5 3.0 2.5 2.0 1.5 1.0 0.5 0.0
ppm

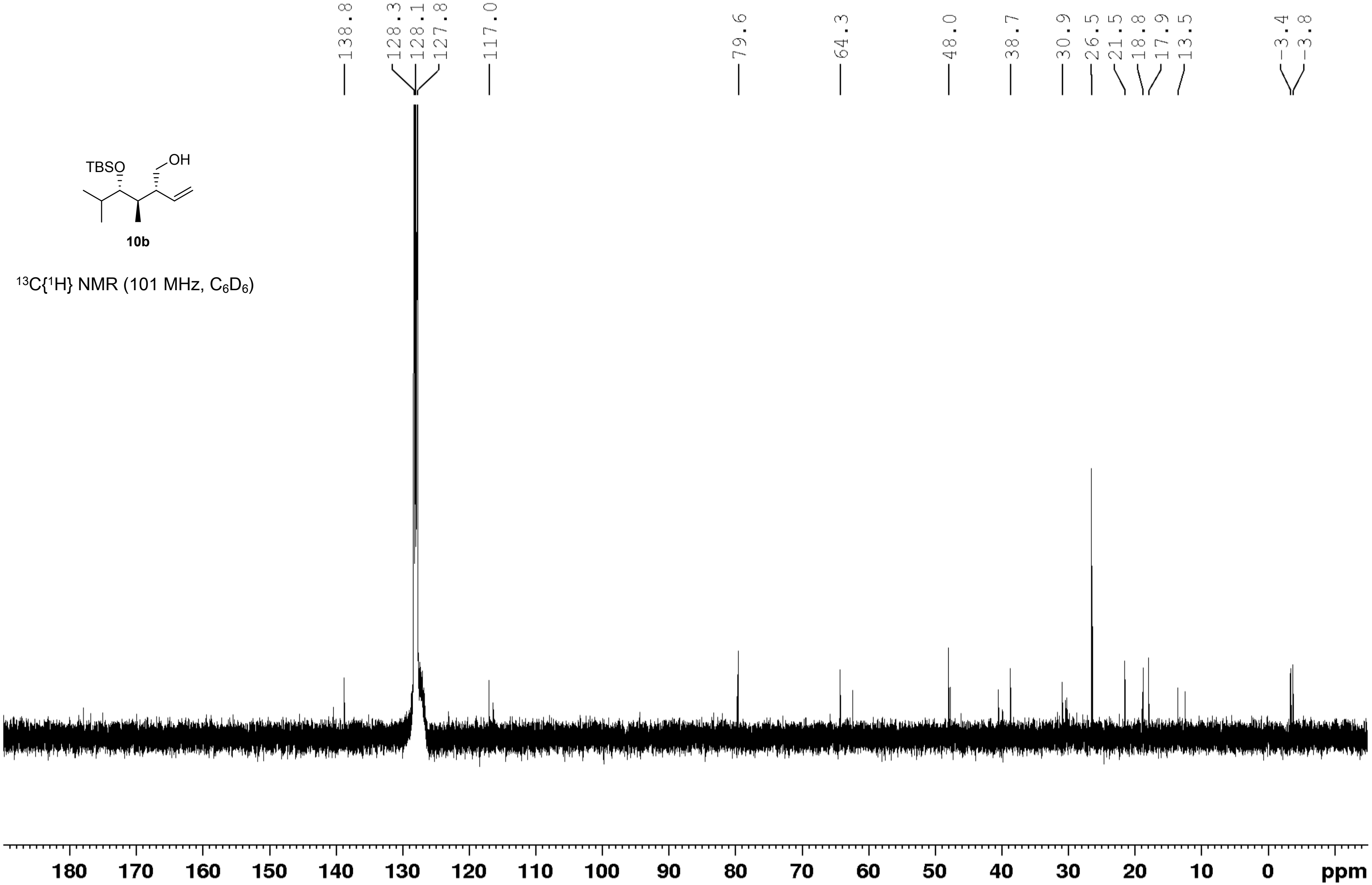
TBSO
OH
10b
$^{13}C\{^{1}H\}$ NMR (101 MHz, C_6D_6)
138.8
128.3
128.1
127.8
117.0
79.6
64.3
48.0
38.7
30.9
26.5
21.5
18.8
17.9
13.5
-3.4
-3.8
180 170 160 150 140 130 120 110 100 90 80 70 60 50 40 30 20 10 0 ppm

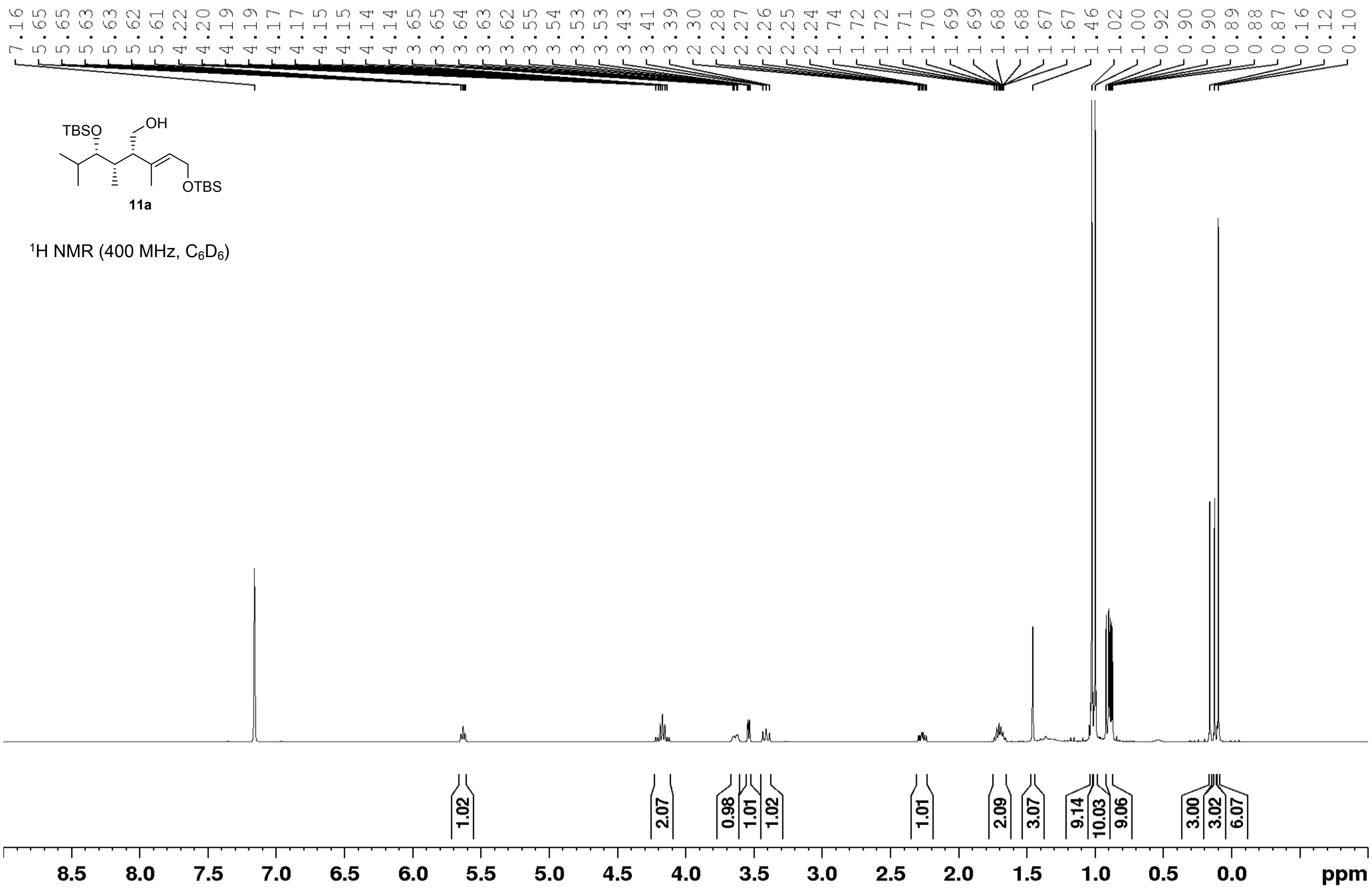
TBSO
OH
OTBS
11a
1H NMR (400 MHz, C6D6)
7.16 5.65 5.65 5.63 5.63 5.62 5.61 4.22 4.20 4.19 4.19 4.17 4.17 4.15 4.15 4.14 4.14 3.65 3.65 3.64 3.63 3.62 3.55 3.54 3.53 3.53 3.43 3.41 3.39 2.30 2.28 2.27 2.26 2.25 2.24 1.74 1.72 1.72 1.71 1.70 1.69 1.69 1.68 1.68 1.67 1.67 1.46 1.02 1.00 0.92 0.90 0.90 0.89 0.88 0.87 0.16 0.12 0.10
1.02
2.07
0.98
1.01
1.02
1.01
2.09
3.07
9.14
10.03
9.06
3.00
3.02
6.07
8.5 8.0 7.5 7.0 6.5 6.0 5.5 5.0 4.5 4.0 3.5 3.0 2.5 2.0 1.5 1.0 0.5 0.0 ppm

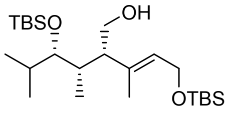

11a

$^{13}C\{^{1}H\}$ NMR (101 MHz, C_6D_6)

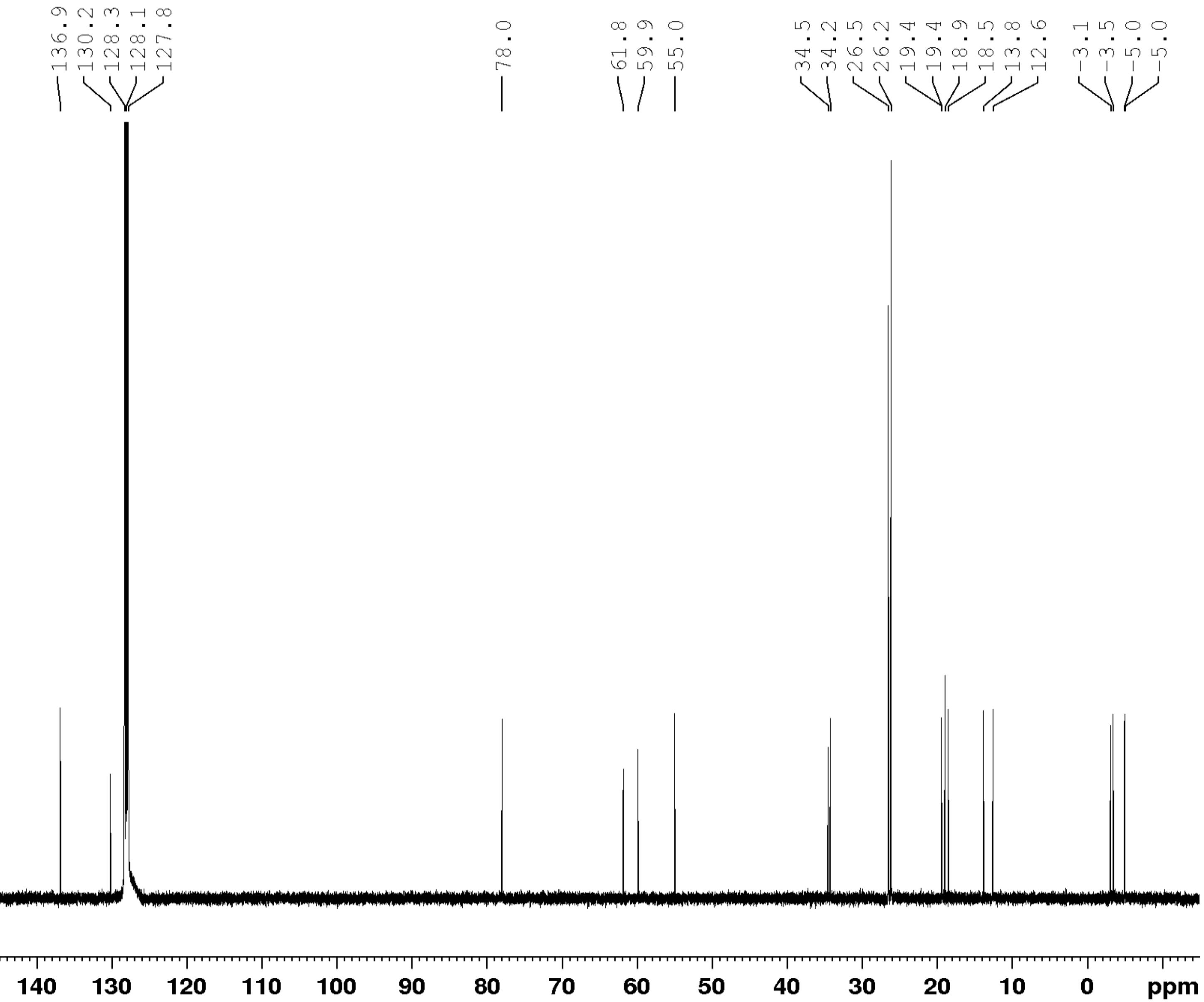

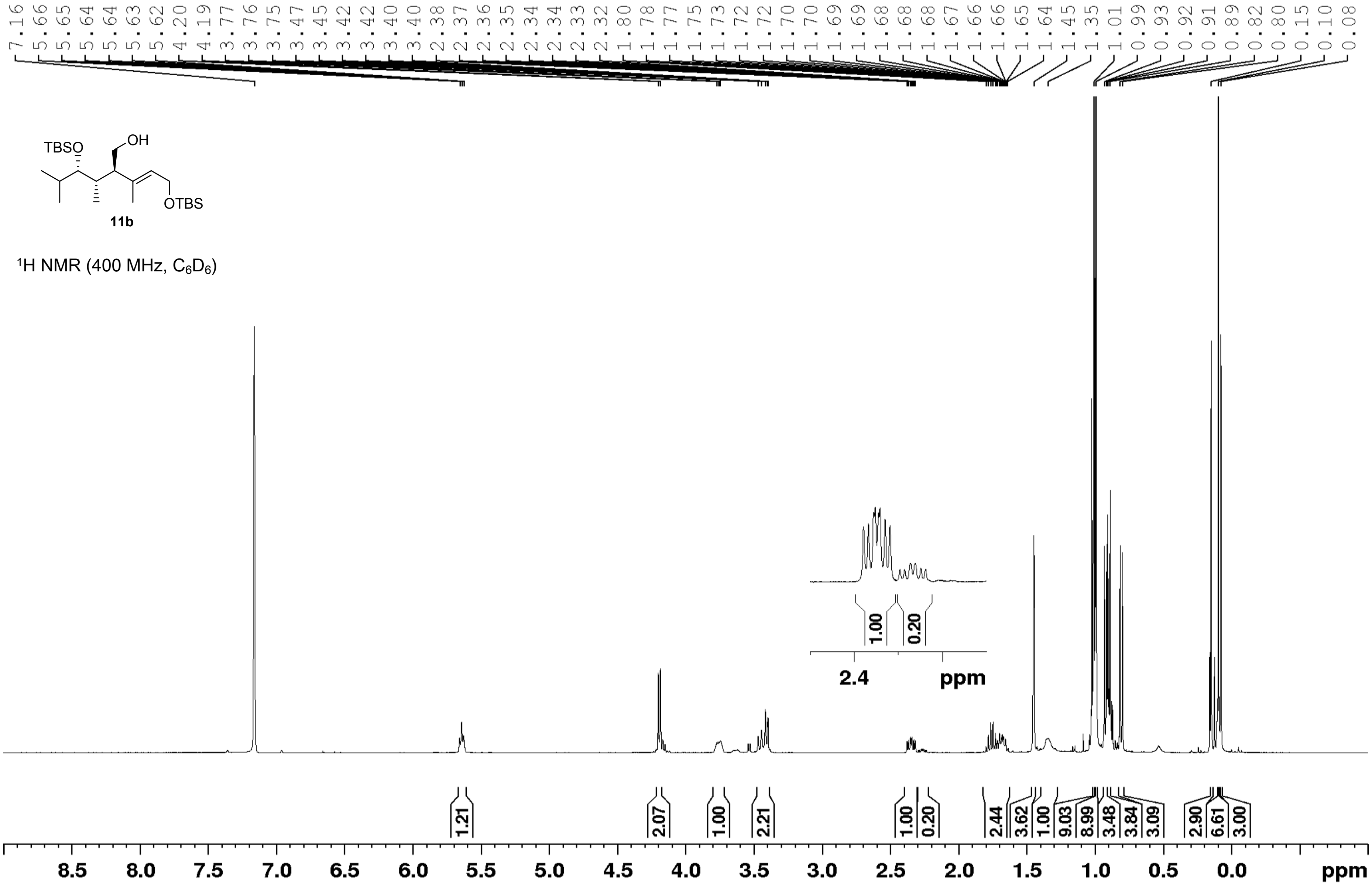
7.16 5.66 5.65 5.64 5.64 5.63 5.62 4.20 4.19 3.77 3.76 3.75 3.47 3.45 3.42 3.42 3.40 3.40 2.38 2.37 2.36 2.35 2.34 2.34 2.33 2.32 1.80 1.78 1.77 1.75 1.73 1.72 1.72 1.70 1.70 1.69 1.69 1.68 1.68 1.67 1.66 1.66 1.65 1.64 1.45 1.35 1.01 0.99 0.93 0.92 0.91 0.89 0.82 0.80 0.15 0.10 0.08
TBSO
OH
OTBS
11b
^{1}H NMR (400 MHz, C_6D_6)
1.00 0.20
2.4 ppm
1.21 2.07 1.00 2.21 1.00 0.20 2.44 3.62 1.00 9.03 8.99 3.48 3.84 3.09 2.90 6.61 3.00
8.5 8.0 7.5 7.0 6.5 6.0 5.5 5.0 4.5 4.0 3.5 3.0 2.5 2.0 1.5 1.0 0.5 0.0 ppm

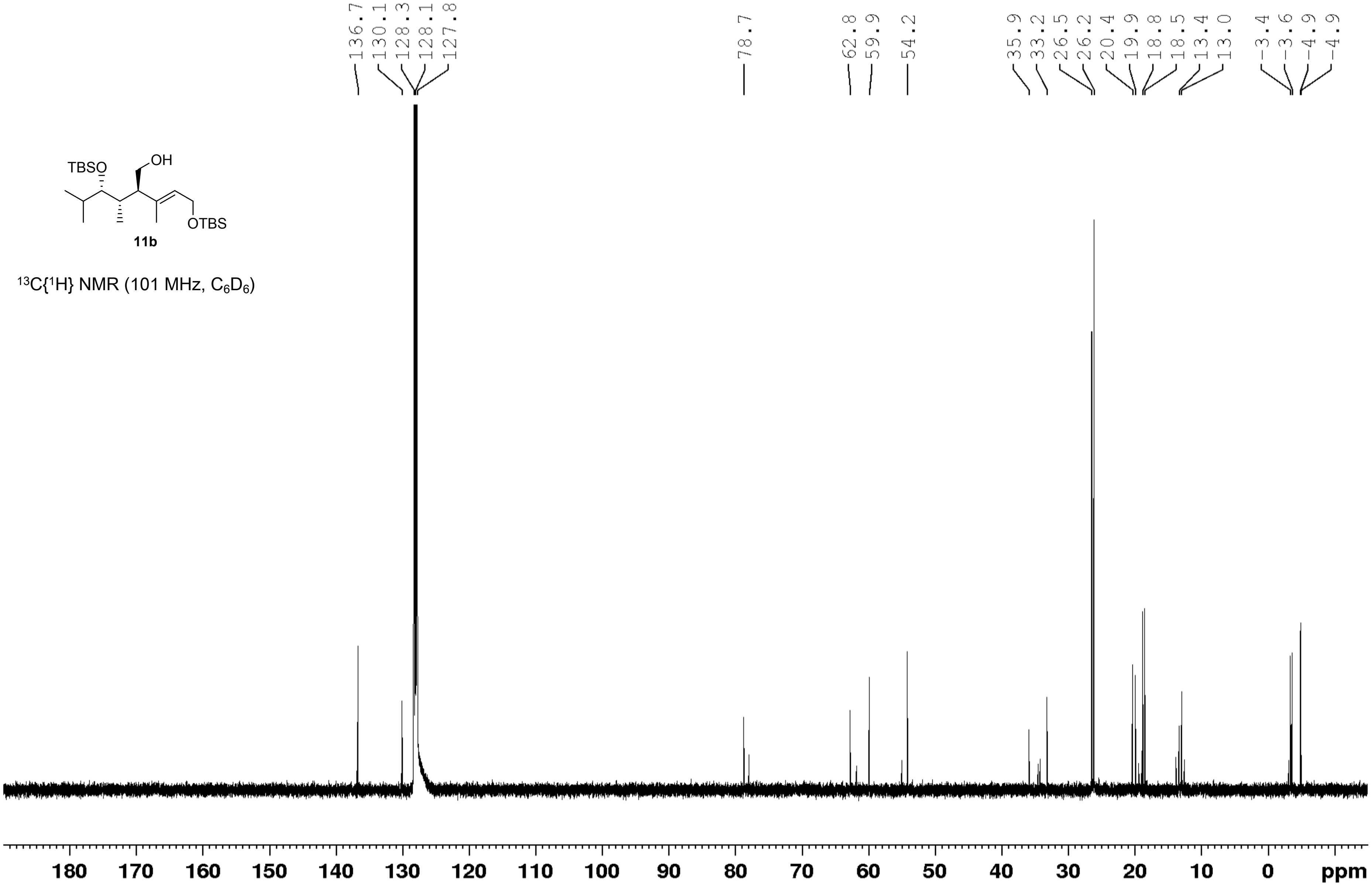

TBSO
OH
OTBS
11b
$^{13}C\{^{1}H\}$ NMR (101 MHz, C_6D_6)
136.7
130.1
128.3
128.1
127.8
78.7
62.8
59.9
54.2
35.9
33.2
26.5
26.2
20.4
19.9
18.8
18.5
13.4
13.0
-3.4
-3.6
-4.9
-4.9
180 170 160 150 140 130 120 110 100 90 80 70 60 50 40 30 20 10 0 ppm

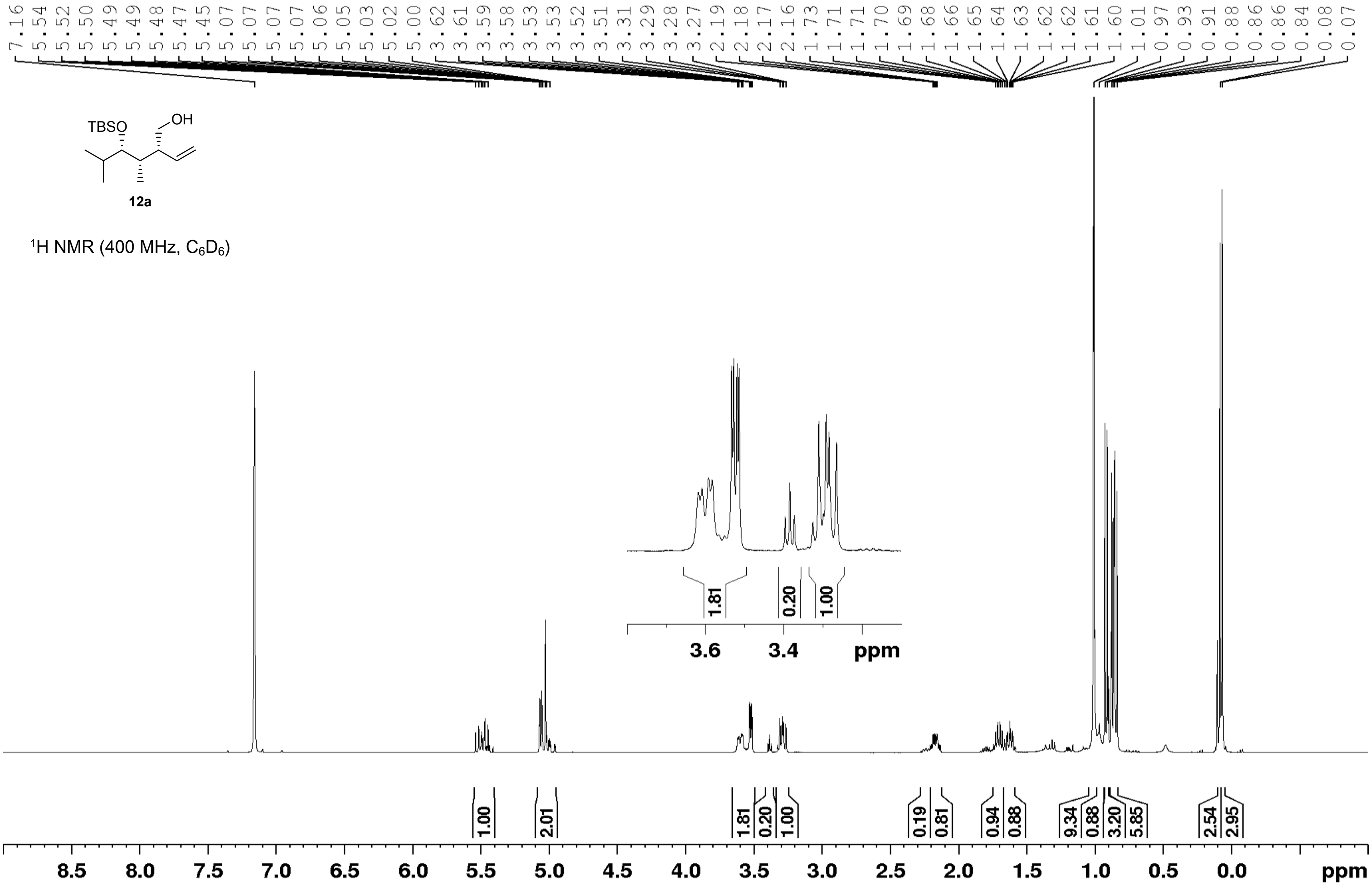
TBSO
OH
12a
1H NMR (400 MHz, C6D6)
7.16 5.54 5.52 5.50 5.49 5.49 5.48 5.47 5.45 5.07 5.07 5.07 5.07 5.06 5.05 5.03 5.02 5.00 3.62 3.61 3.59 3.58 3.53 3.53 3.52 3.51 3.31 3.29 3.28 3.27 2.19 2.18 2.17 2.16 1.73 1.71 1.71 1.70 1.69 1.68 1.66 1.65 1.64 1.63 1.62 1.62 1.61 1.60 1.01 0.97 0.93 0.91 0.88 0.86 0.86 0.84 0.08 0.07
1.81
0.20
1.00
3.6
3.4
ppm
1.00
2.01
1.81
0.20
1.00
0.19
0.81
0.94
0.88
9.34
0.88
3.20
5.85
2.54
2.95
8.5 8.0 7.5 7.0 6.5 6.0 5.5 5.0 4.5 4.0 3.5 3.0 2.5 2.0 1.5 1.0 0.5 0.0 ppm

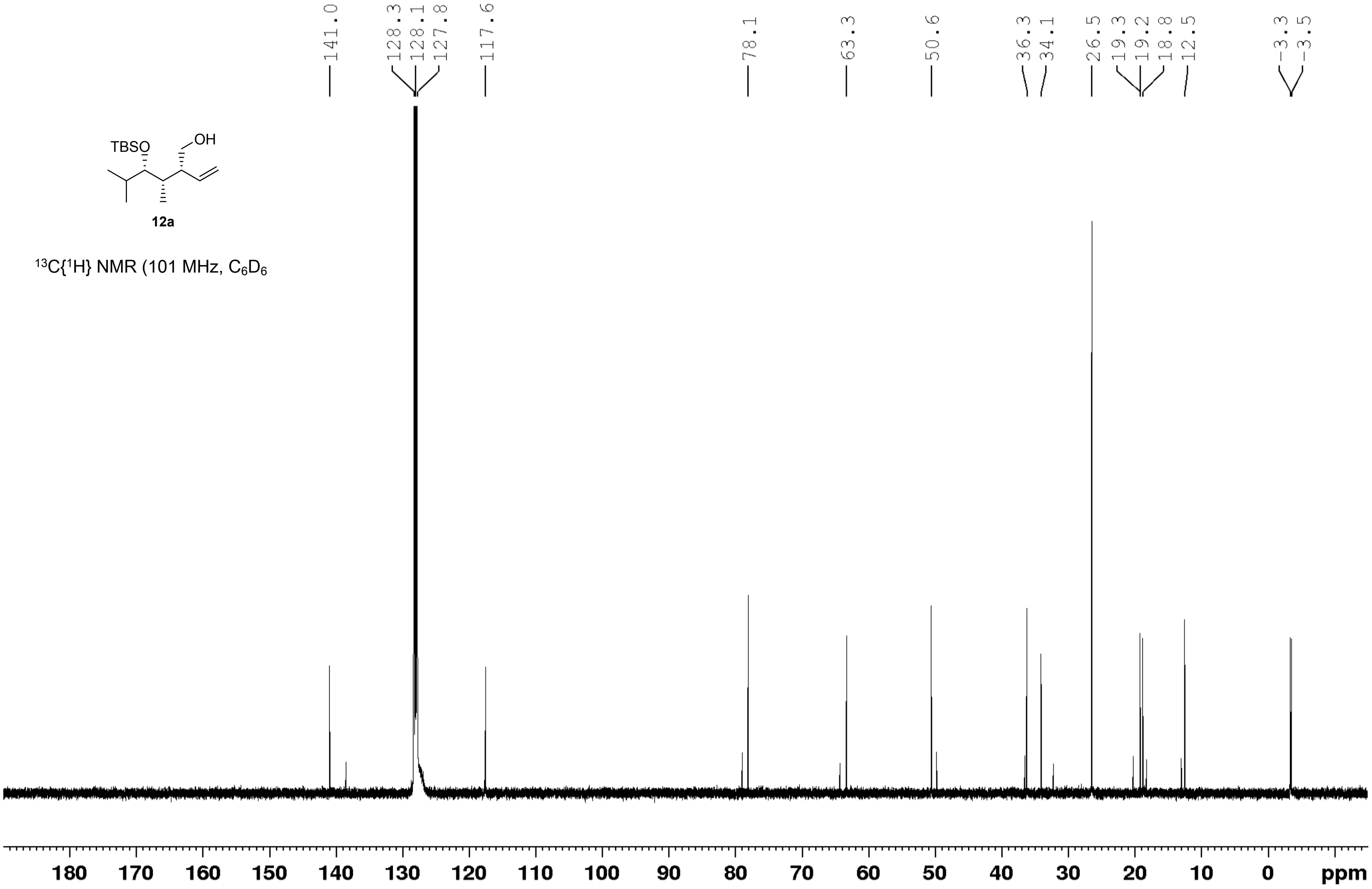
141.0
128.3
128.1
127.8
117.6
78.1
63.3
50.6
36.3
34.1
26.5
19.3
19.2
18.8
12.5
-3.3
-3.5
TBSO
OH
12a
13C{1H} NMR (101 MHz, C6D6
180 170 160 150 140 130 120 110 100 90 80 70 60 50 40 30 20 10 0 ppm

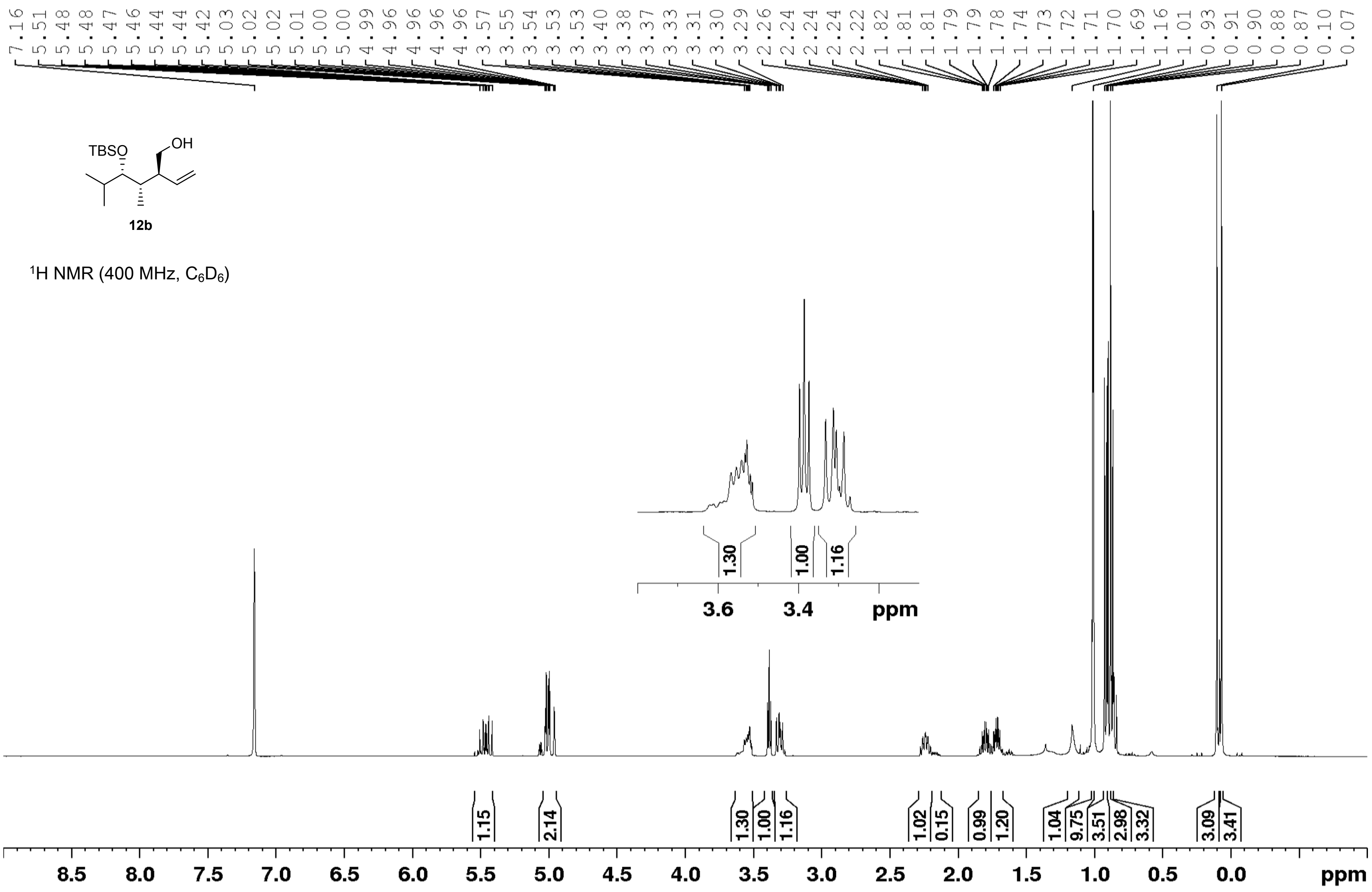
7.16 5.51 5.48 5.48 5.47 5.46 5.44 5.44 5.42 5.03 5.02 5.02 5.01 5.00 5.00 4.99 4.96 4.96 4.96 4.96 3.57 3.55 3.54 3.53 3.53 3.40 3.38 3.37 3.33 3.31 3.30 3.29 2.26 2.24 2.24 2.24 2.22 2.22 1.82 1.81 1.81 1.79 1.79 1.78 1.74 1.73 1.72 1.71 1.70 1.69 1.16 1.01 0.93 0.91 0.90 0.88 0.87 0.10 0.07
TBSO
OH
12b
1H NMR (400 MHz, C6D6)
1.30
1.00
1.16
3.6
3.4
ppm
1.15
2.14
1.30
1.00
1.16
1.02
0.15
0.99
1.20
1.04
9.75
3.51
2.98
3.32
3.09
3.41
8.5
8.0
7.5
7.0
6.5
6.0
5.5
5.0
4.5
4.0
3.5
3.0
2.5
2.0
1.5
1.0
0.5
0.0
ppm

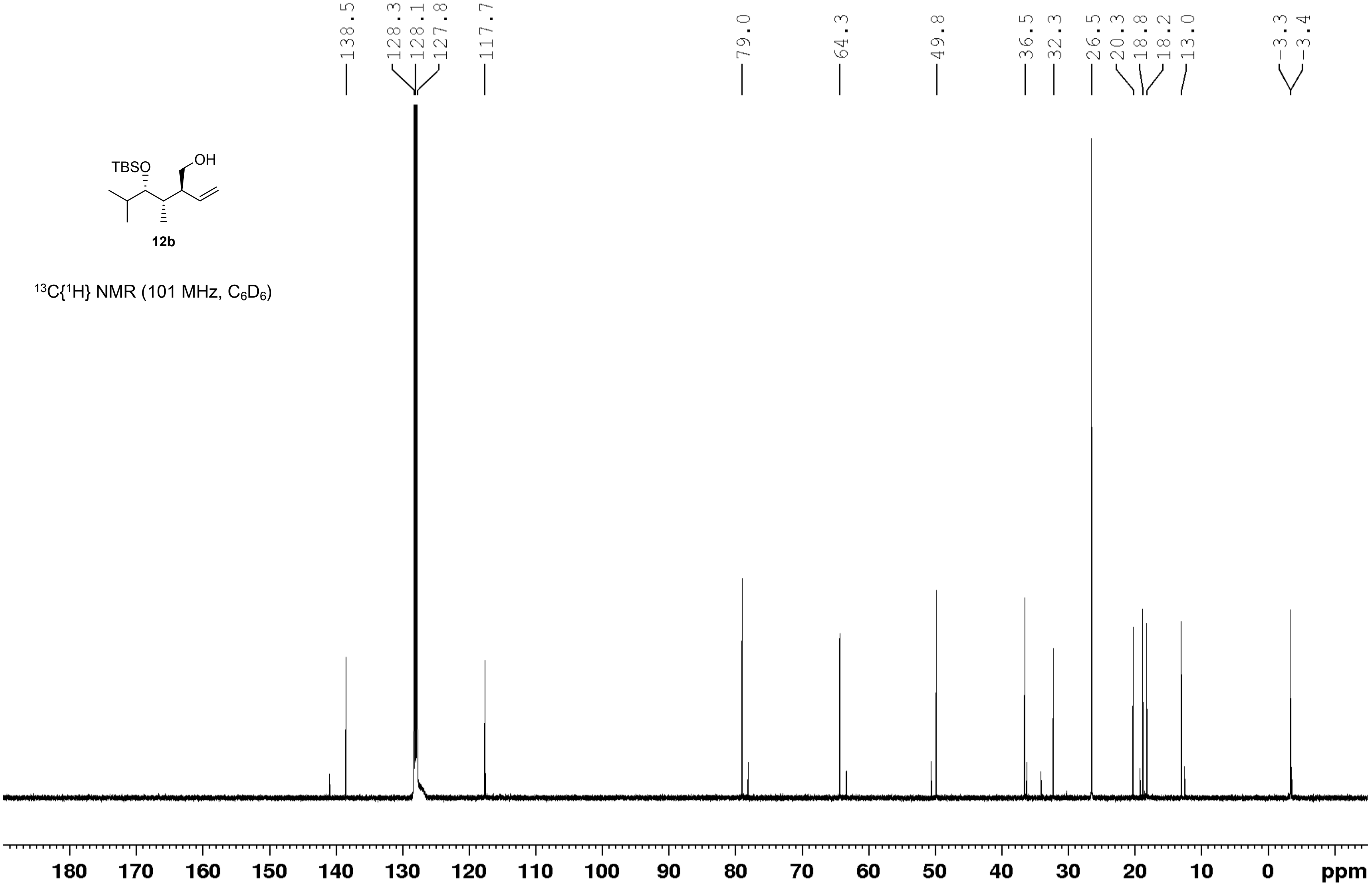
138.5
128.3
128.1
127.8
117.7
79.0
64.3
49.8
36.5
32.3
26.5
20.3
18.8
18.2
13.0
-3.3
-3.4
TBSO
OH
12b
$^{13}C\{^1H\}$ NMR (101 MHz, C_6D_6)
180
170
160
150
140
130
120
110
100
90
80
70
60
50
40
30
20
10
0
ppm

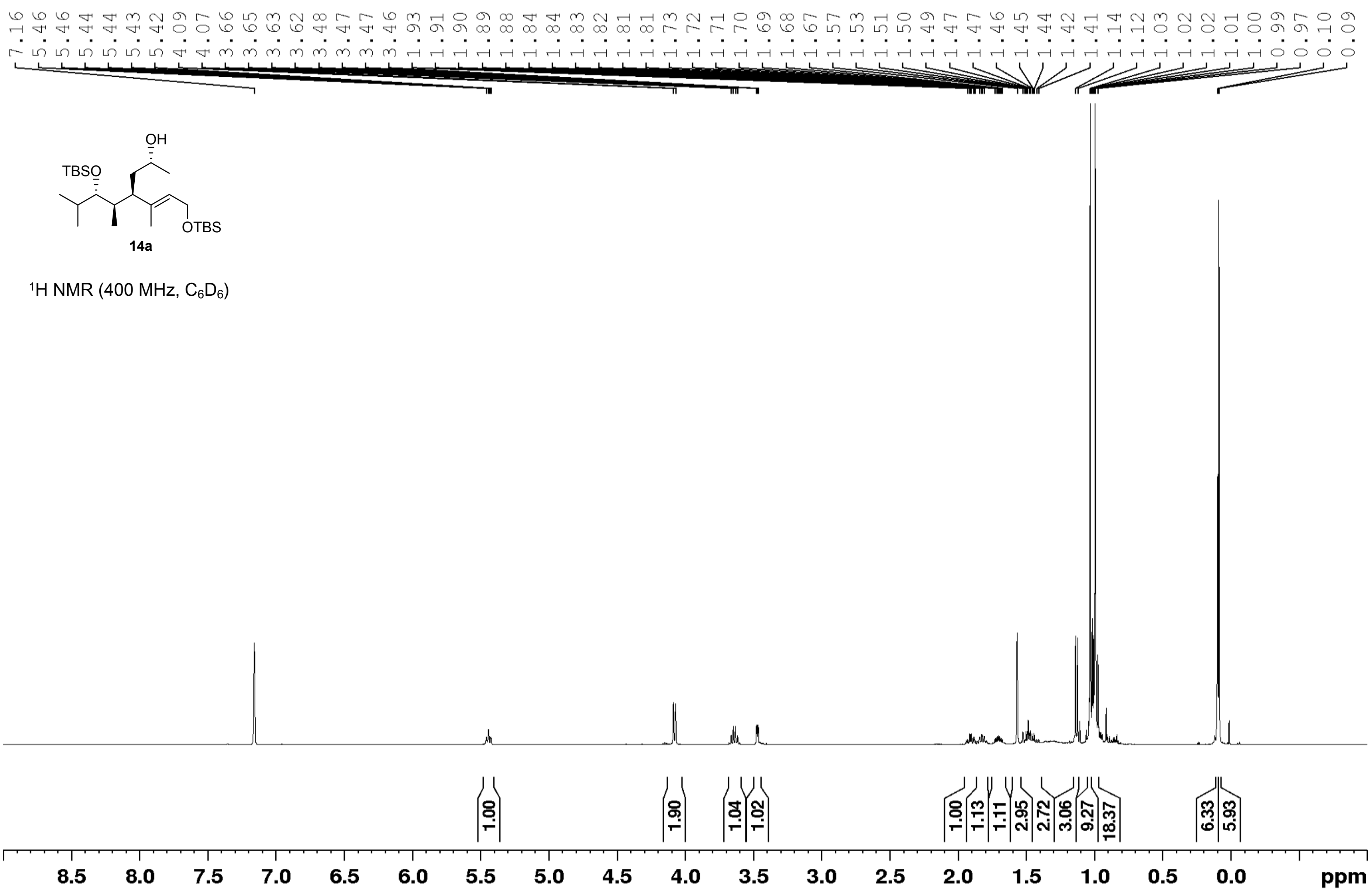
7.16 5.46 5.46 5.44 5.44 5.43 5.42 4.09 4.07 3.66 3.65 3.63 3.62 3.48 3.47 3.47 3.46 1.93 1.91 1.90 1.89 1.88 1.84 1.84 1.83 1.82 1.81 1.81 1.73 1.72 1.71 1.70 1.69 1.68 1.67 1.57 1.53 1.51 1.50 1.49 1.47 1.47 1.46 1.45 1.44 1.42 1.41 1.14 1.12 1.03 1.02 1.02 1.01 1.00 0.99 0.97 0.10 0.09
OH
TBSO
OTBS
14a
^{1}H NMR (400 MHz, C_6D_6)
1.00
1.90
1.04
1.02
1.00
1.13
1.11
2.95
2.72
3.06
9.27
18.37
6.33
5.93
8.5 8.0 7.5 7.0 6.5 6.0 5.5 5.0 4.5 4.0 3.5 3.0 2.5 2.0 1.5 1.0 0.5 0.0
ppm

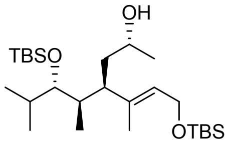

14a

$^{13}C\{^1H\}$ NMR (101 MHz, C_6D_6)

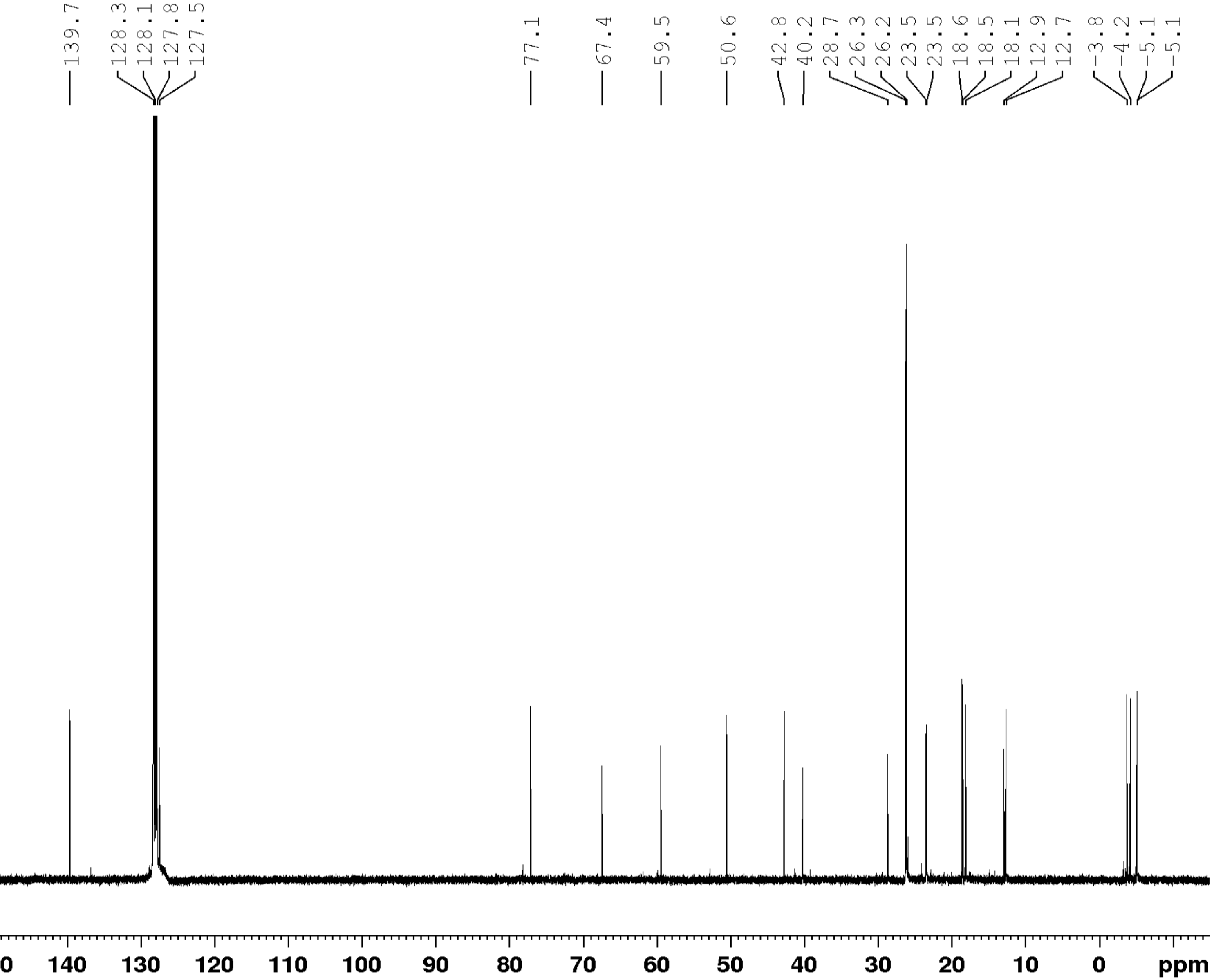

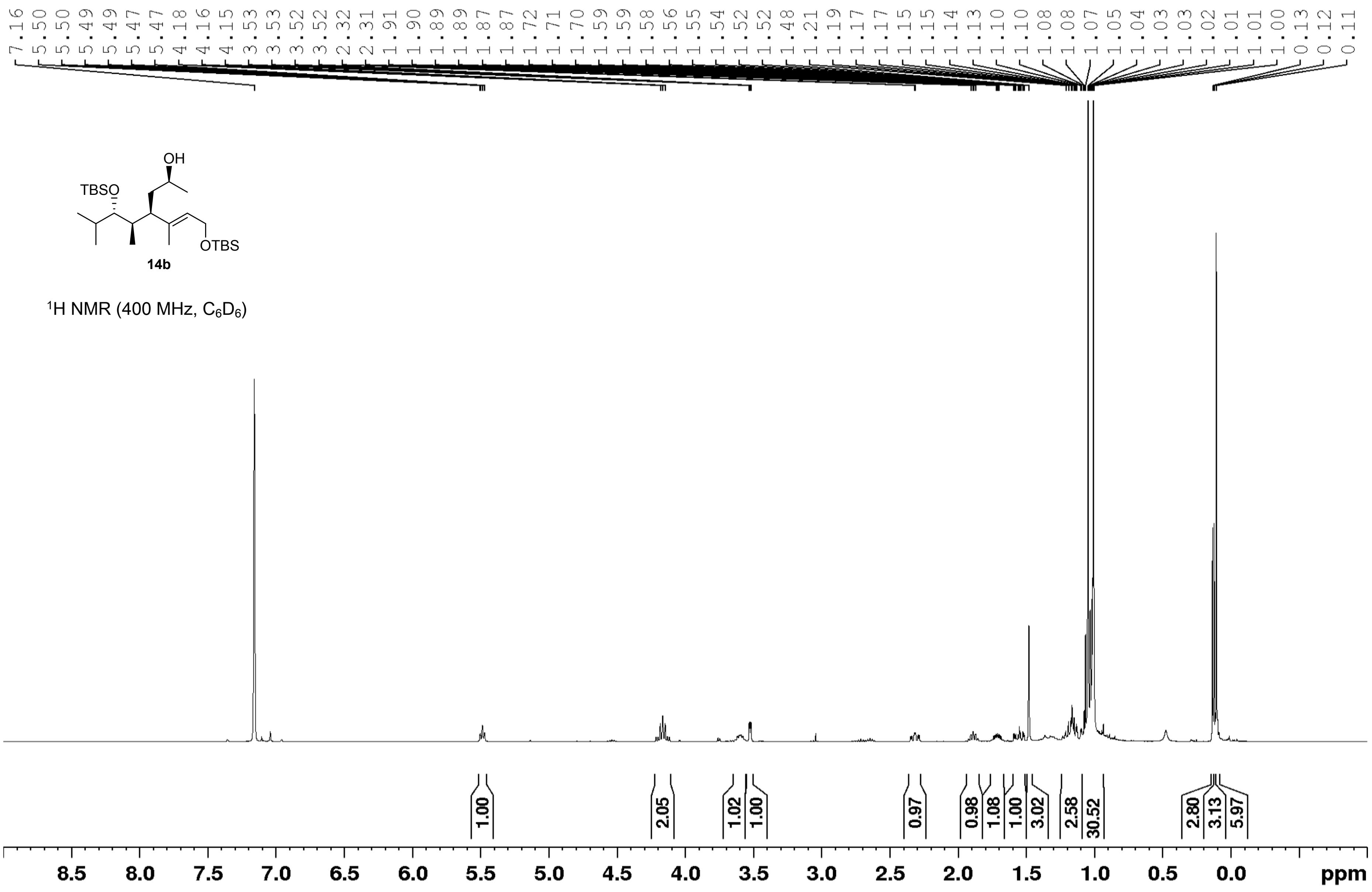
7.16 5.50 5.50 5.49 5.49 5.47 5.47 4.18 4.16 4.15 3.53 3.53 3.52 3.52 2.32 2.31 1.91 1.90 1.89 1.89 1.87 1.87 1.72 1.71 1.70 1.59 1.59 1.58 1.56 1.55 1.54 1.52 1.52 1.48 1.21 1.19 1.17 1.17 1.15 1.15 1.14 1.13 1.10 1.10 1.08 1.08 1.07 1.05 1.04 1.03 1.03 1.02 1.01 1.01 1.00 0.13 0.12 0.11
OH
TBSO
OTBS
14b
1H NMR (400 MHz, C6D6)
1.00
2.05
1.02
1.00
0.97
0.98
1.08
1.00
3.02
2.58
30.52
2.80
3.13
5.97
8.5 8.0 7.5 7.0 6.5 6.0 5.5 5.0 4.5 4.0 3.5 3.0 2.5 2.0 1.5 1.0 0.5 0.0 ppm

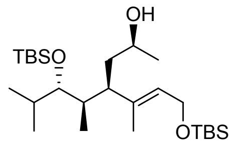

14b

$^{13}C\{^{1}H\}$ NMR (101 MHz, C_6D_6)

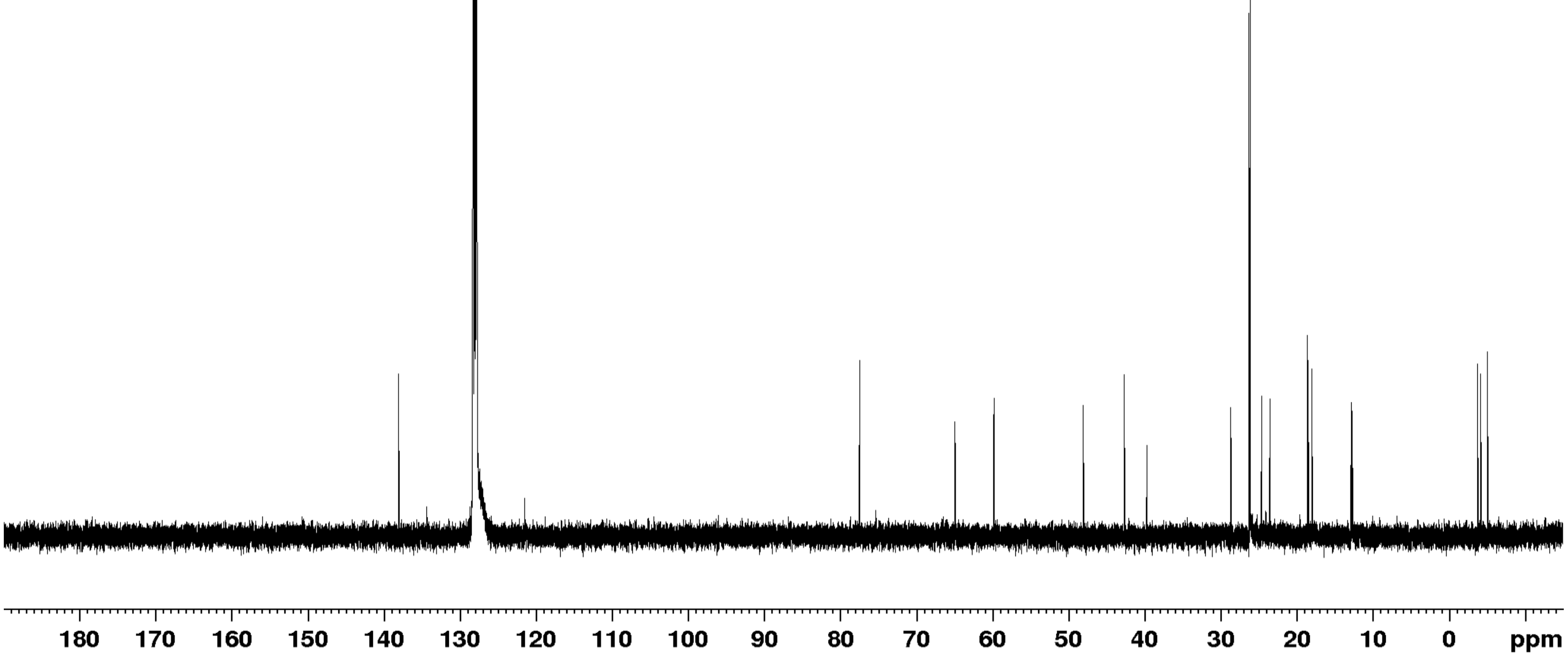

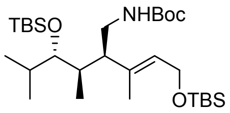

17

^1H NMR (400 MHz, C_6D_6)

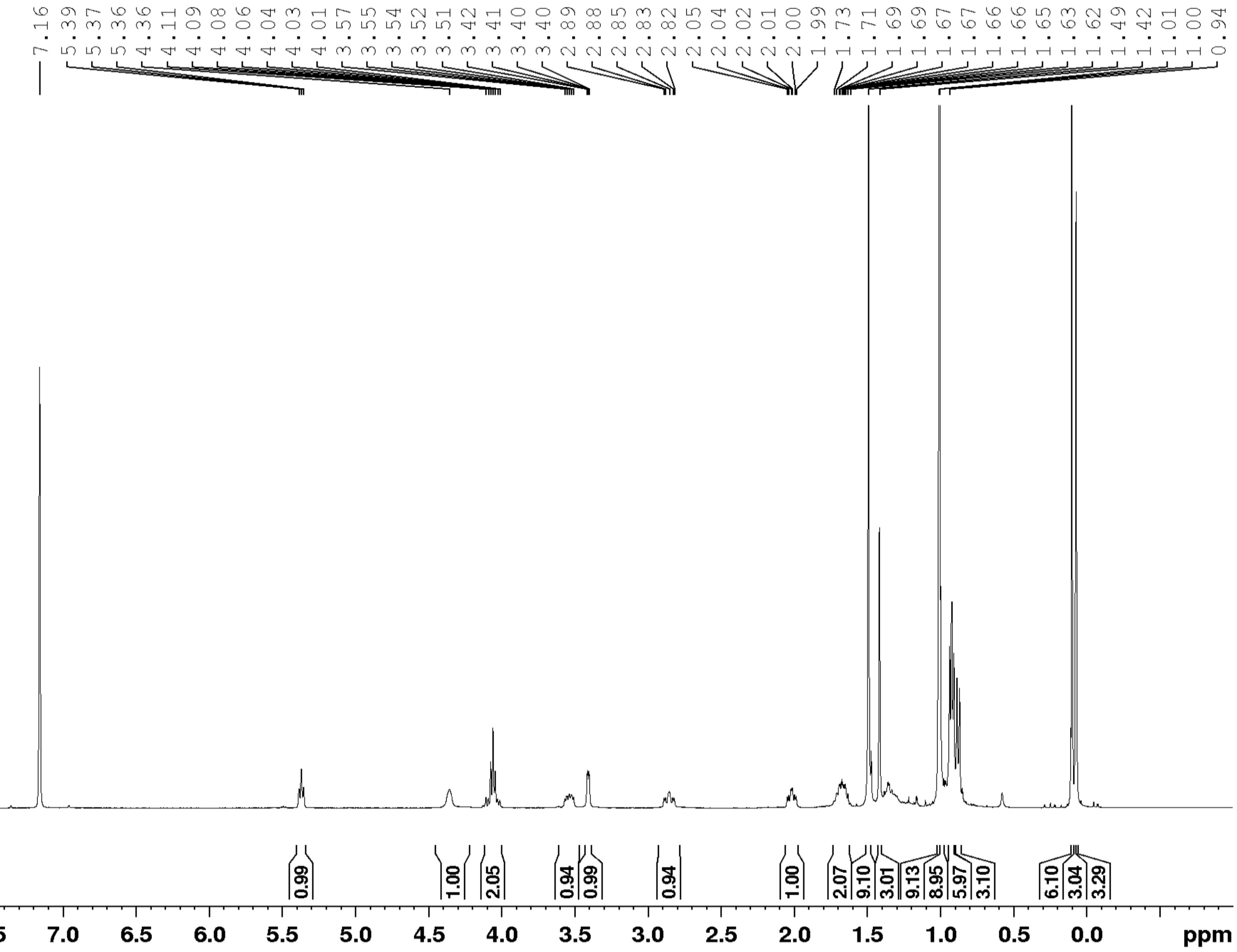

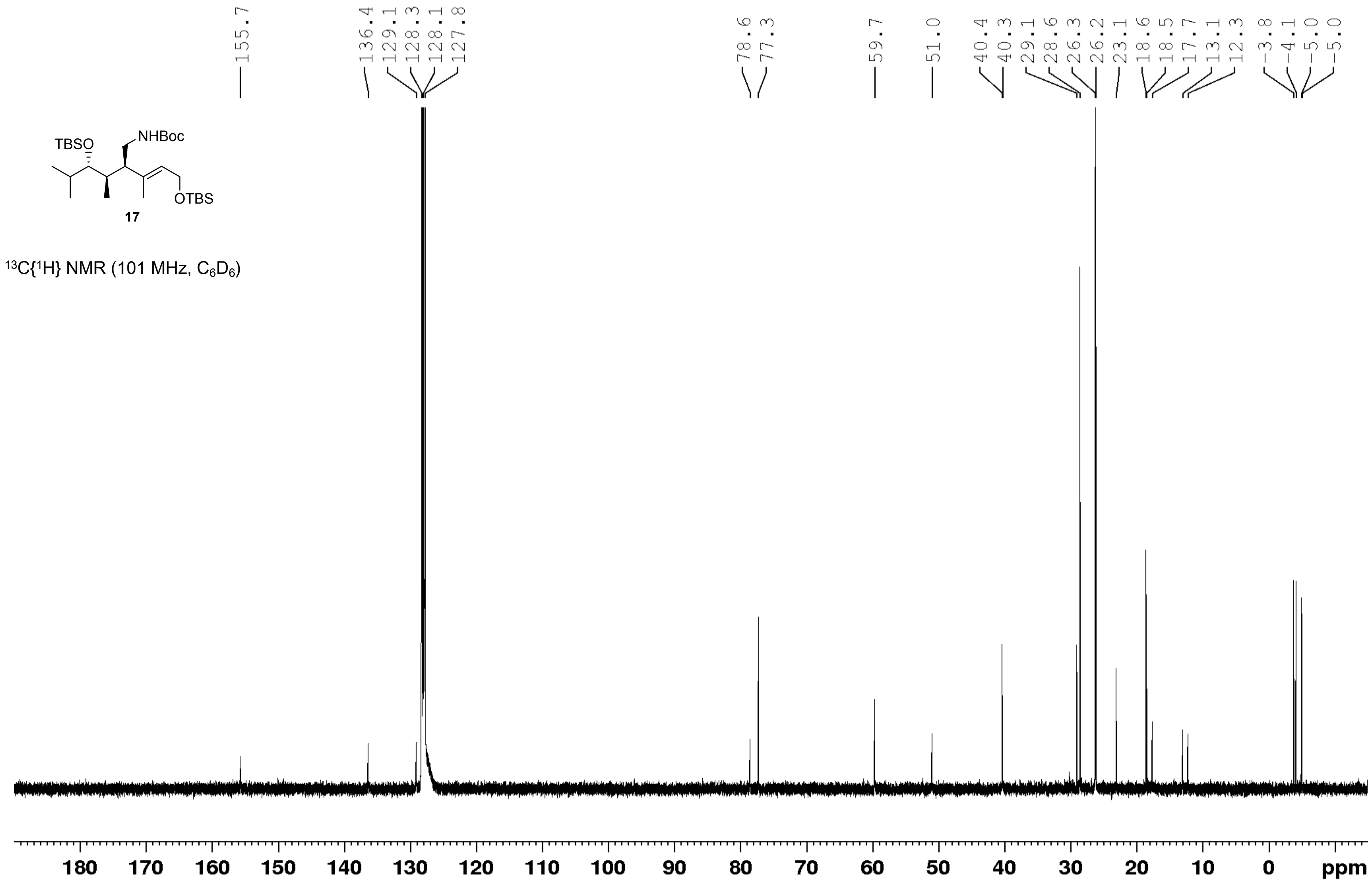
155.7
136.4
129.1
128.3
128.1
127.8
78.6
77.3
59.7
51.0
40.4
40.3
29.1
28.6
26.3
26.2
23.1
18.6
18.5
17.7
13.1
12.3
-3.8
-4.1
-5.0
-5.0
TBSO
NHBoc
OTBS
17
$^{13}C\{^{1}H\}$ NMR (101 MHz, C_6D_6)
180 170 160 150 140 130 120 110 100 90 80 70 60 50 40 30 20 10 0 ppm

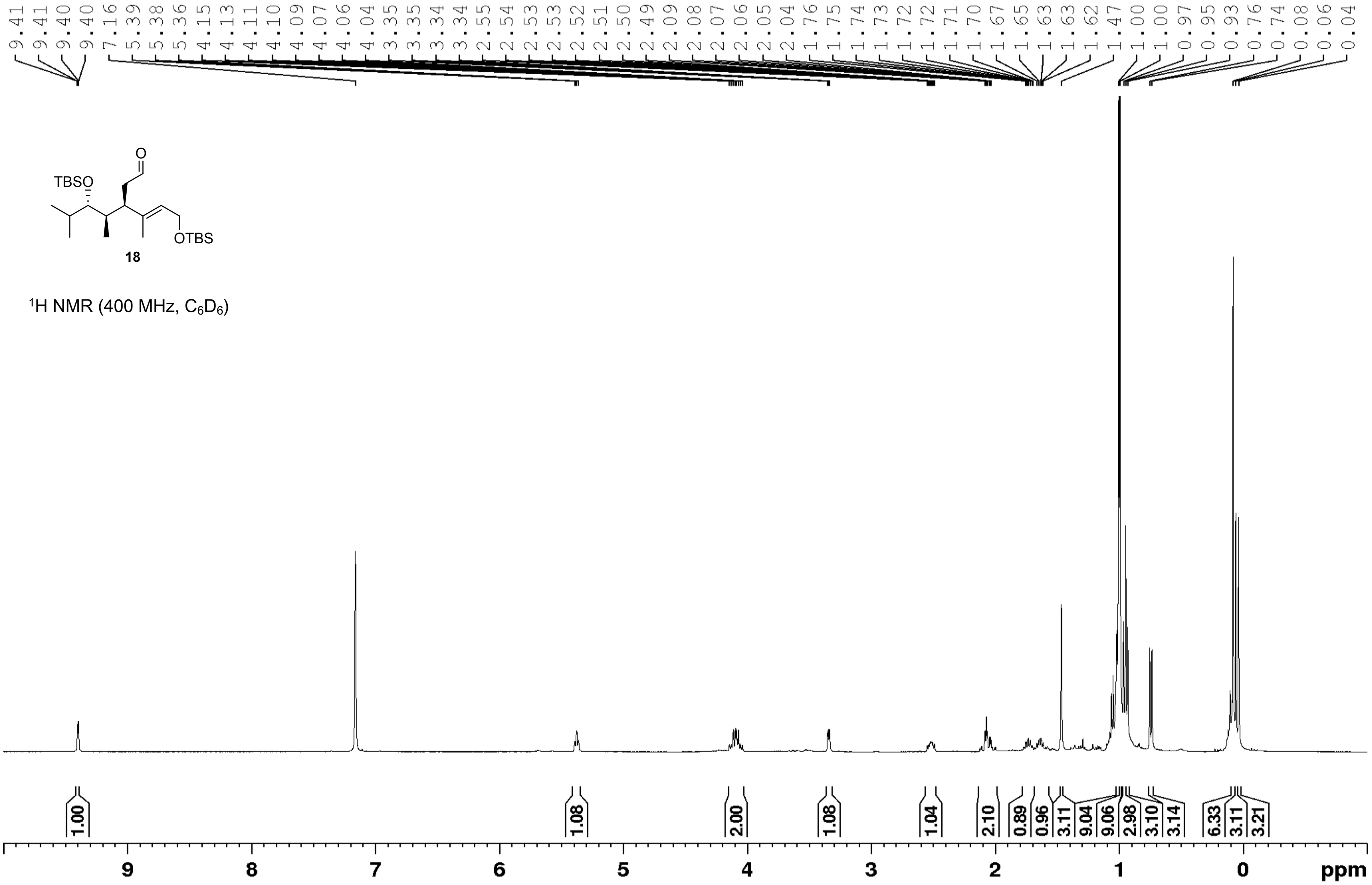
9.41 9.41 9.40 9.40 7.16 5.39 5.38 5.36 4.15 4.13 4.11 4.10 4.09 4.07 4.06 4.04 3.35 3.35 3.34 3.34 2.55 2.54 2.53 2.53 2.52 2.51 2.50 2.49 2.09 2.08 2.07 2.06 2.05 2.04 1.76 1.75 1.74 1.73 1.72 1.72 1.71 1.70 1.67 1.65 1.63 1.63 1.62 1.47 1.00 1.00 0.97 0.95 0.93 0.76 0.74 0.08 0.06 0.04
O
TBSO
OTBS
18
1H NMR (400 MHz, C6D6)
1.00 1.08 2.00 1.08 1.04 2.10 0.89 0.96 3.11 9.04 9.06 2.98 3.10 3.14 6.33 3.11 3.21
9 8 7 6 5 4 3 2 1 0 ppm

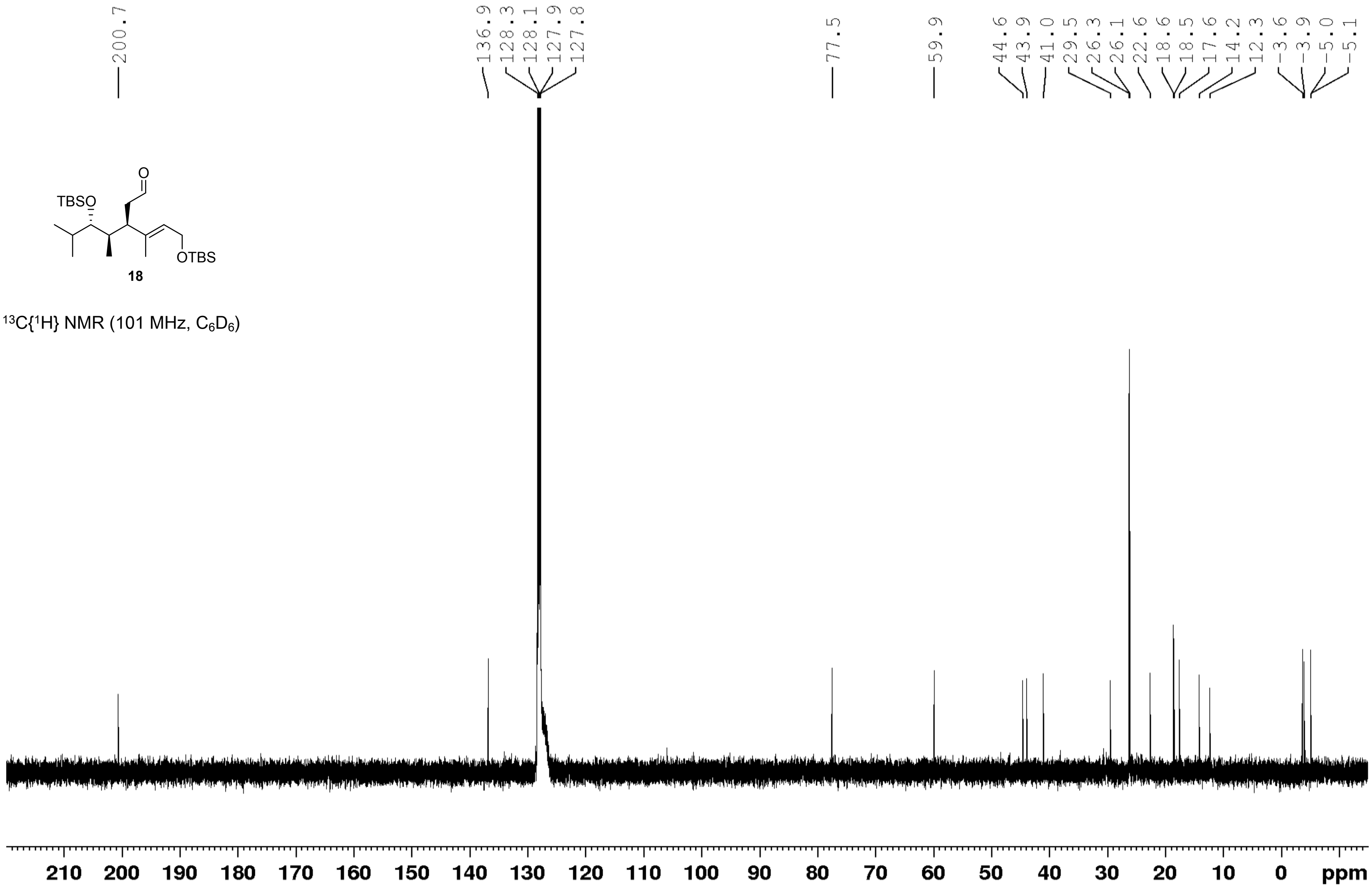

200.7
136.9
128.3
128.1
127.9
127.8
77.5
59.9
44.6
43.9
41.0
29.5
26.3
26.1
22.6
18.6
18.5
17.6
14.2
12.3
-3.6
-3.9
-5.0
-5.1
TBSO
O
OTBS
18
$^{13}C\{^1H\}$ NMR (101 MHz, C_6D_6)
210 200 190 180 170 160 150 140 130 120 110 100 90 80 70 60 50 40 30 20 10 0 ppm

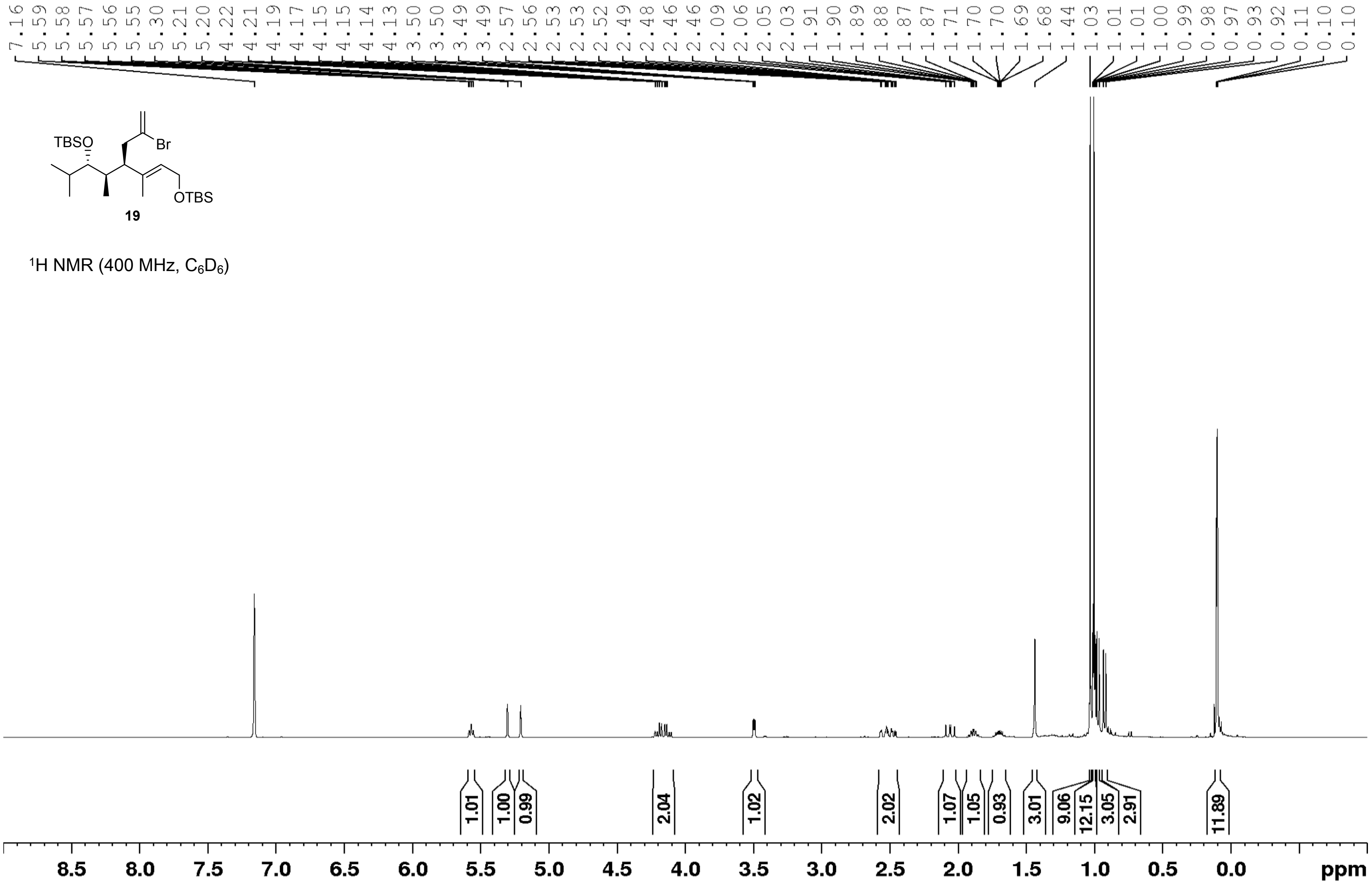
7.16 5.59 5.58 5.57 5.56 5.55 5.30 5.21 5.20 4.22 4.21 4.19 4.17 4.15 4.15 4.14 4.13 3.50 3.50 3.49 3.49 2.57 2.56 2.53 2.53 2.52 2.49 2.48 2.46 2.46 2.09 2.06 2.05 2.03 1.91 1.90 1.89 1.88 1.87 1.87 1.71 1.70 1.70 1.69 1.68 1.44 1.03 1.01 1.01 1.00 0.99 0.98 0.97 0.93 0.92 0.11 0.10 0.10
TBSO
Br
OTBS
19
1H NMR (400 MHz, C6D6)
1.01 1.00 0.99 2.04 1.02 2.02 1.07 1.05 0.93 3.01 9.06 12.15 3.05 2.91 11.89
8.5 8.0 7.5 7.0 6.5 6.0 5.5 5.0 4.5 4.0 3.5 3.0 2.5 2.0 1.5 1.0 0.5 0.0 ppm

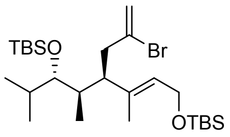

19

$^{13}C\{^{1}H\}$ NMR (101 MHz, C_6D_6)

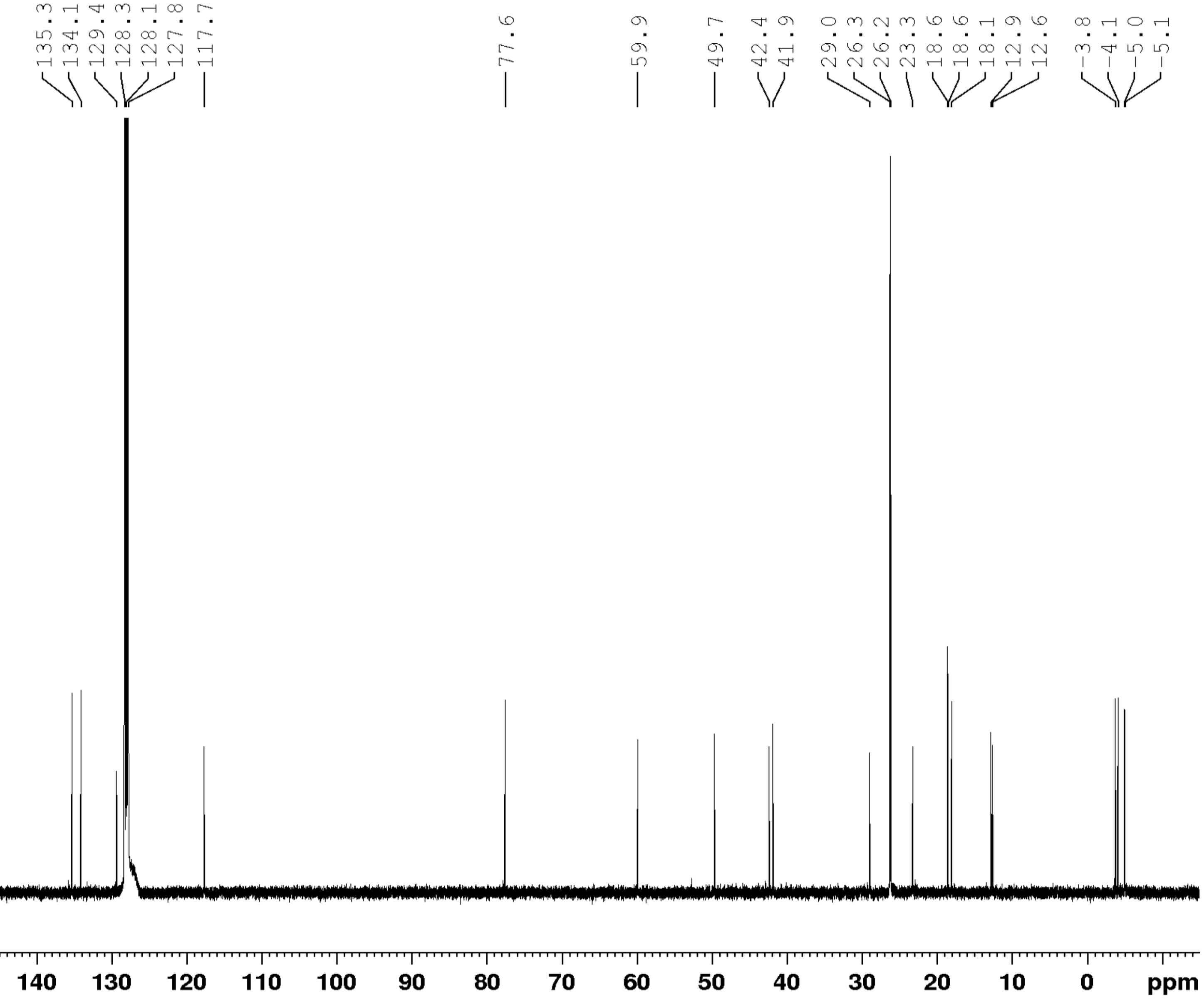

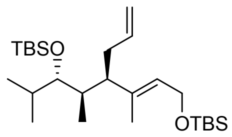

20

^{1}H NMR (400 MHz, C_6D_6)

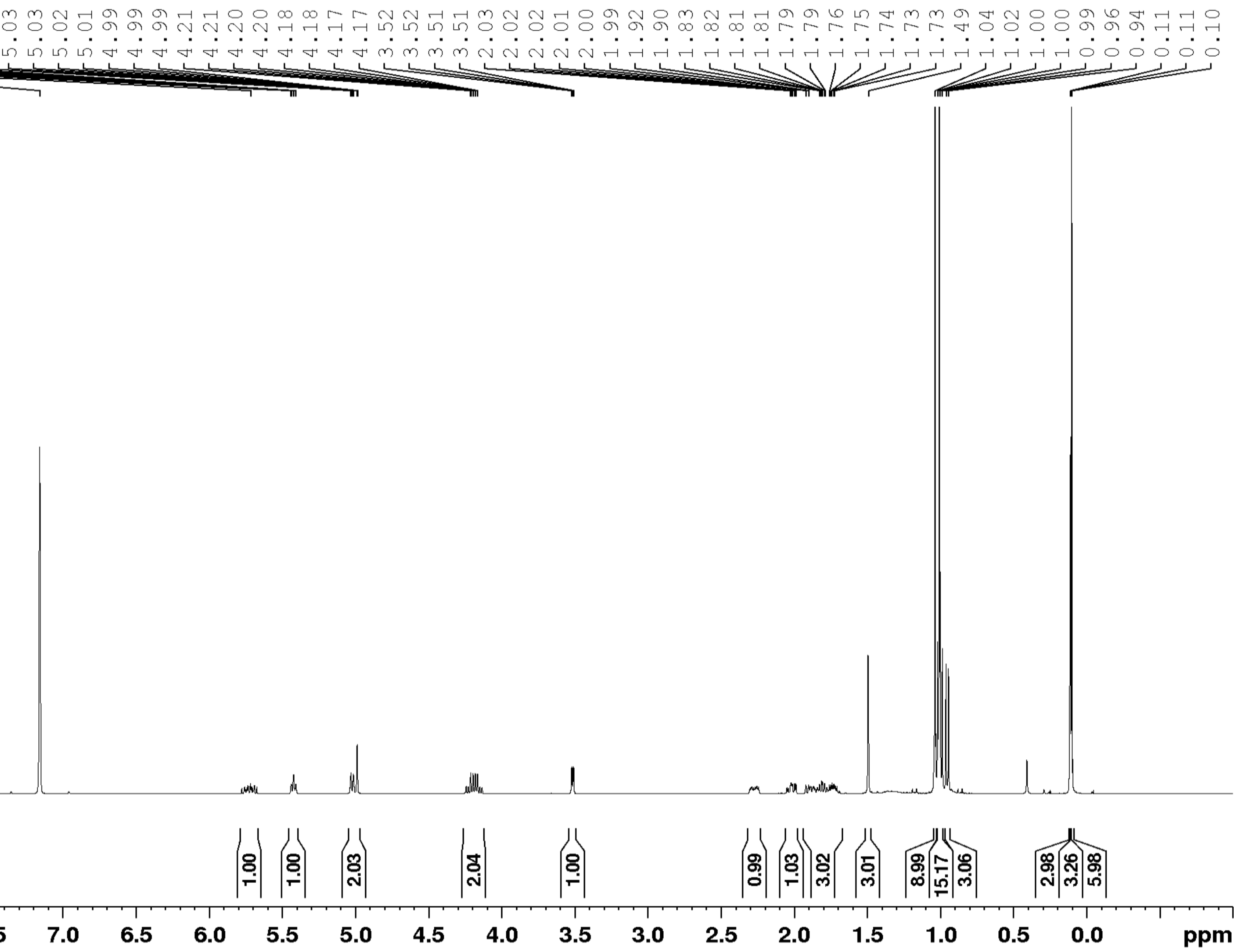

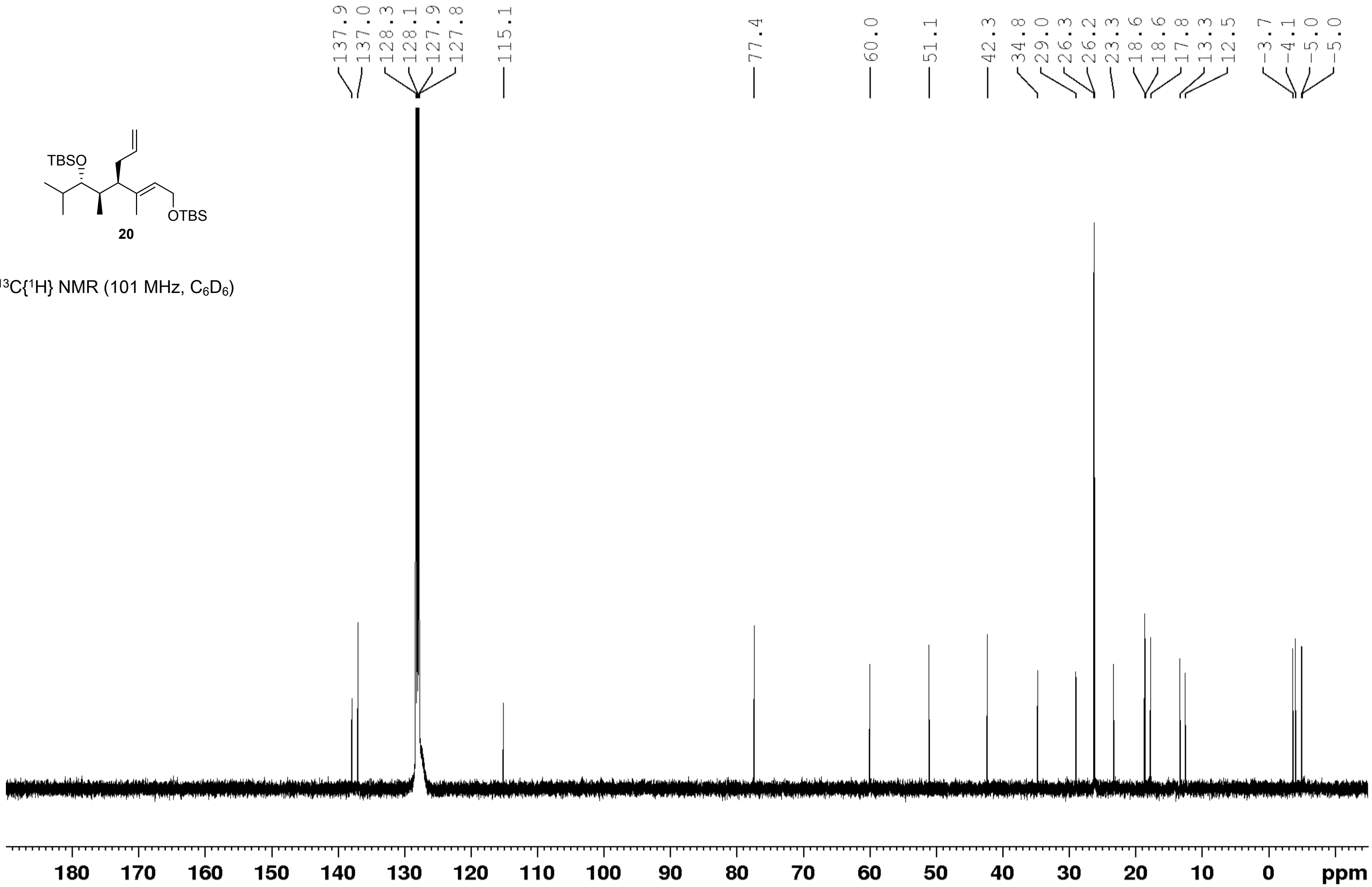

20

$^{13}C\{^{1}H\}$ NMR (101 MHz, C_6D_6)

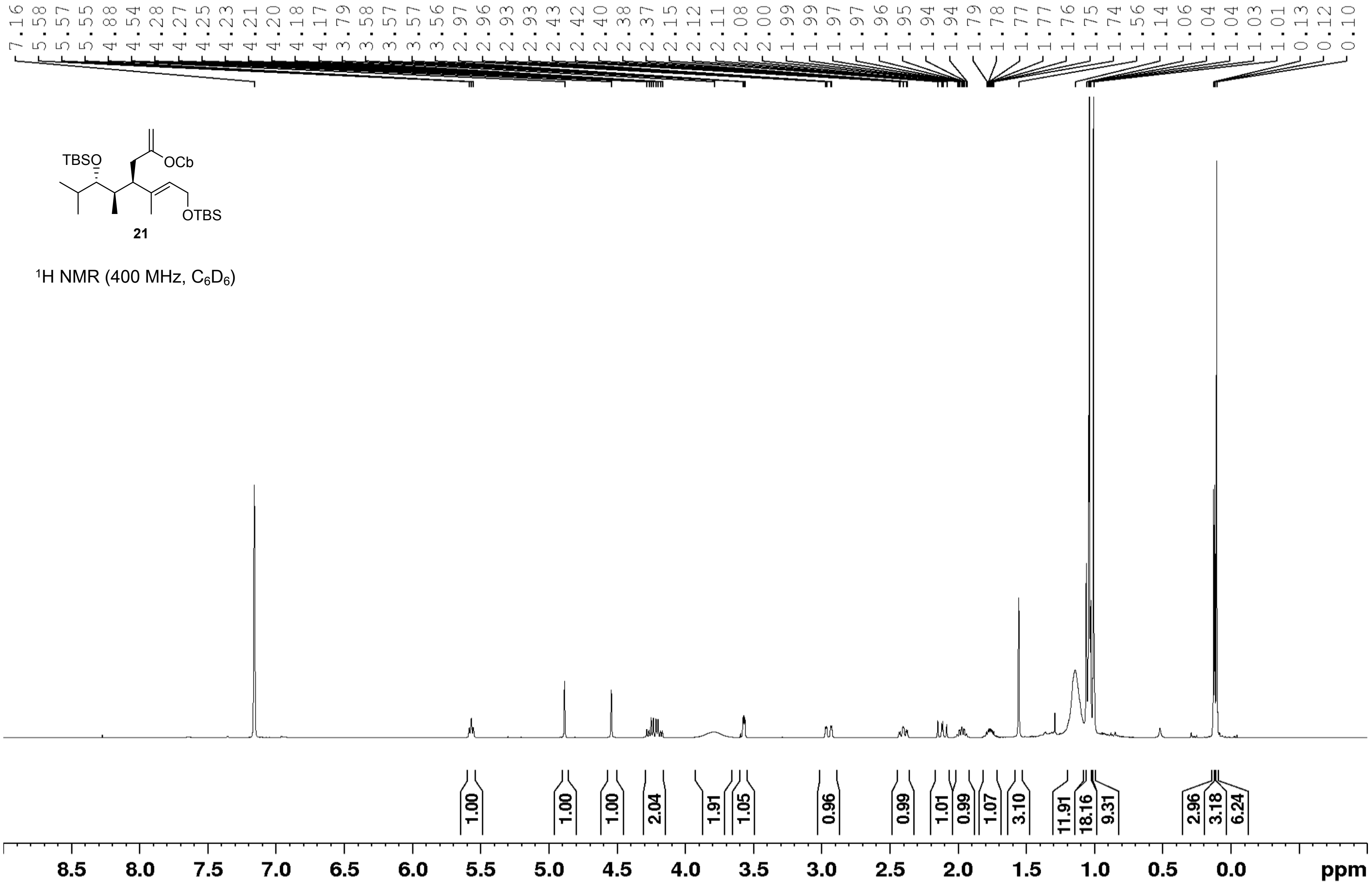
7.16 5.58 5.57 5.55 4.88 4.54 4.28 4.27 4.25 4.23 4.21 4.20 4.18 4.17 3.79 3.58 3.57 3.57 3.56 2.97 2.96 2.93 2.93 2.43 2.42 2.40 2.38 2.37 2.15 2.12 2.11 2.08 2.00 1.99 1.99 1.97 1.97 1.96 1.95 1.94 1.94 1.79 1.78 1.77 1.77 1.76 1.75 1.74 1.56 1.14 1.06 1.04 1.04 1.03 1.01 0.13 0.12 0.10
TBSO
OCb
OTBS
21
1H NMR (400 MHz, C6D6)
1.00 1.00 1.00 2.04 1.91 1.05 0.96 0.99 1.01 0.99 1.07 3.10 11.91 18.16 9.31 2.96 3.18 6.24
8.5 8.0 7.5 7.0 6.5 6.0 5.5 5.0 4.5 4.0 3.5 3.0 2.5 2.0 1.5 1.0 0.5 0.0 ppm

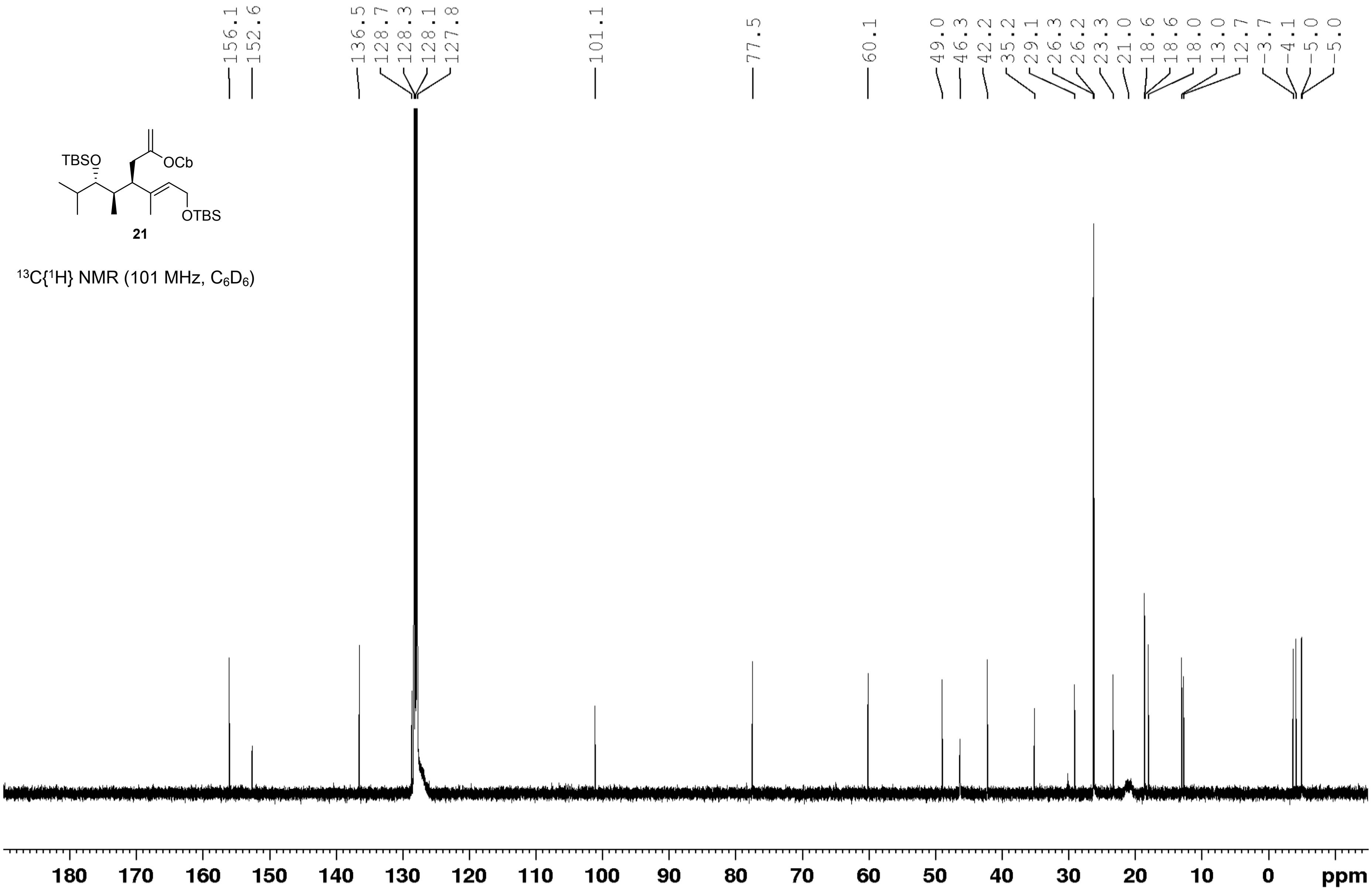
156.1
152.6
136.5
128.7
128.3
128.1
127.8
101.1
77.5
60.1
49.0
46.3
42.2
35.2
29.1
26.3
26.2
23.3
21.0
18.6
18.6
18.0
13.0
12.7
-3.7
-4.1
-5.0
-5.0
TBSO
OCb
OTBS
21
$^{13}C\{^1H\}$ NMR (101 MHz, C_6D_6)
180
170
160
150
140
130
120
110
100
90
80
70
60
50
40
30
20
10
0
ppm

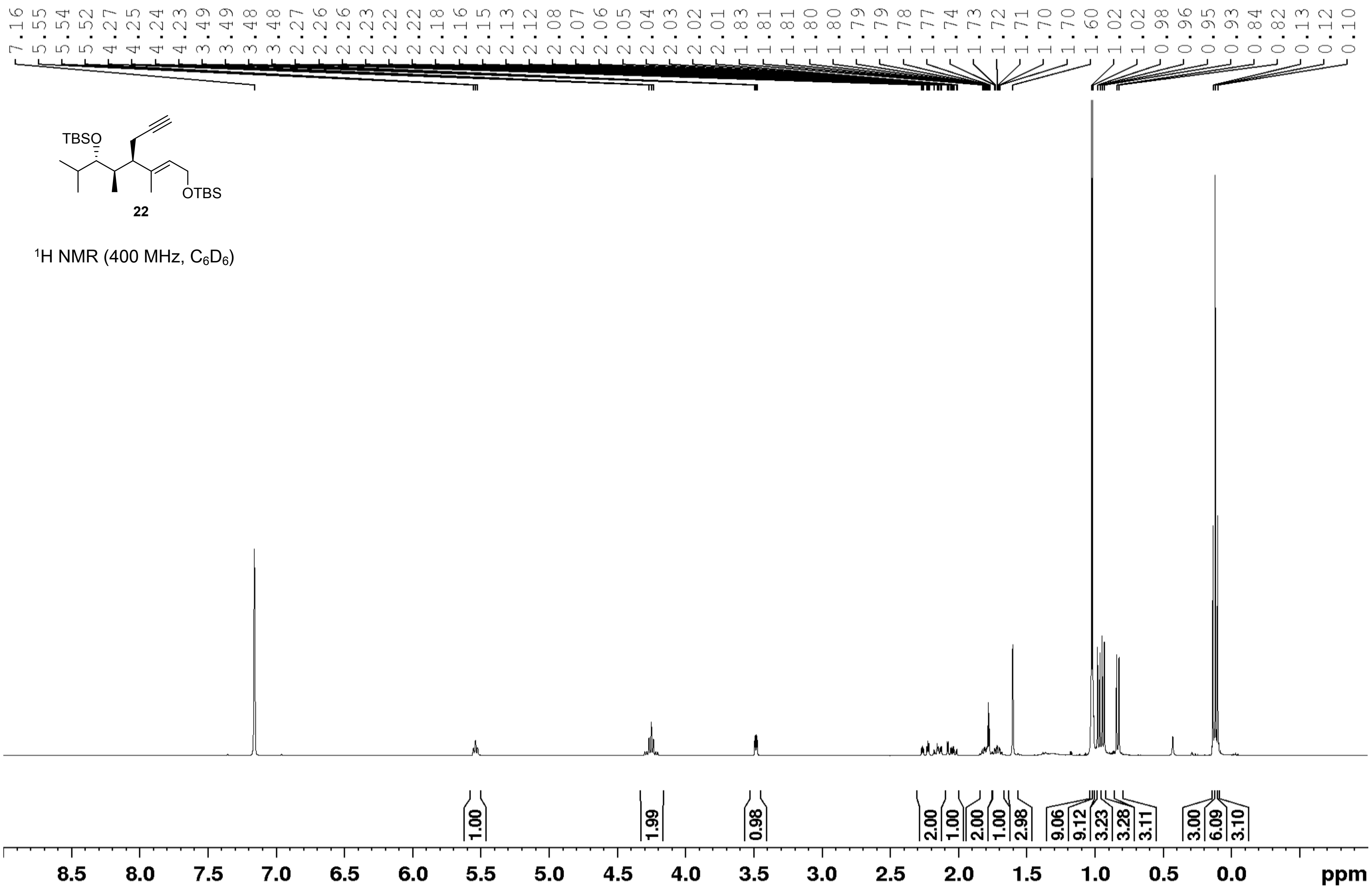
7.16 5.55 5.54 5.52 4.27 4.25 4.24 4.23 3.49 3.49 3.48 3.48 2.27 2.26 2.26 2.23 2.22 2.22 2.18 2.16 2.15 2.13 2.12 2.08 2.07 2.06 2.05 2.04 2.03 2.02 2.01 1.83 1.81 1.81 1.80 1.80 1.79 1.79 1.78 1.77 1.74 1.73 1.72 1.71 1.70 1.70 1.60 1.02 1.02 0.98 0.96 0.95 0.93 0.84 0.82 0.13 0.12 0.10
TBSO
OTBS
22
^{1}H NMR (400 MHz, C_6D_6)
1.00
1.99
0.98
2.00
1.00
2.00
1.00
2.98
9.06
9.12
3.23
3.28
3.11
3.00
6.09
3.10
8.5 8.0 7.5 7.0 6.5 6.0 5.5 5.0 4.5 4.0 3.5 3.0 2.5 2.0 1.5 1.0 0.5 0.0 ppm

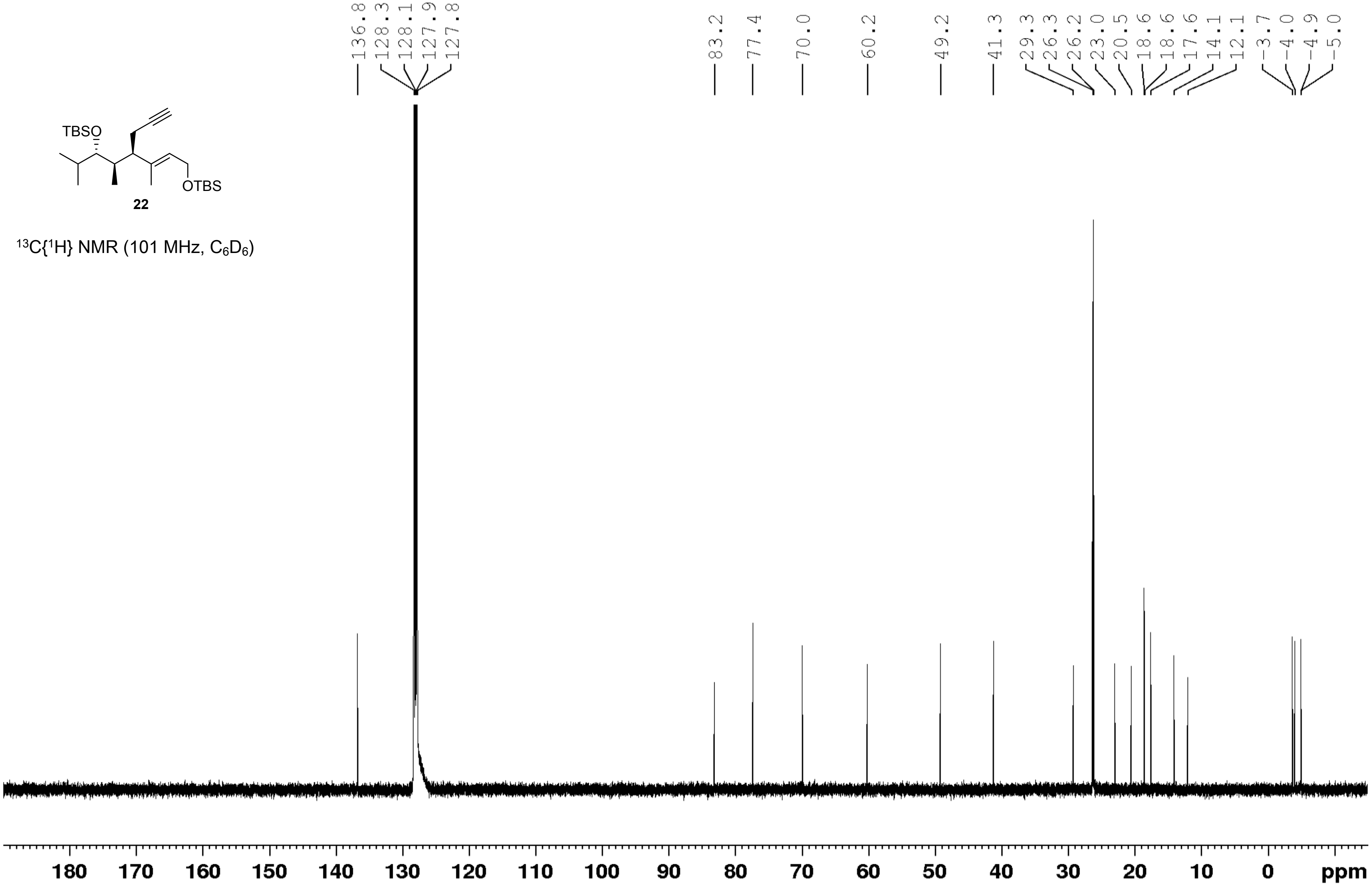

$^{13}C\{^{1}H\}$ NMR (101 MHz, C_6D_6)
